# The draft nuclear genome sequence and predicted mitochondrial proteome of *Andalucia godoyi*, a protist with the most gene-rich and bacteria-like mitochondrial genome

**DOI:** 10.1186/s12915-020-0741-6

**Published:** 2020-03-02

**Authors:** Michael W. Gray, Gertraud Burger, Romain Derelle, Vladimír Klimeš, Michelle M. Leger, Matt Sarrasin, Čestmír Vlček, Andrew J. Roger, Marek Eliáš, B. Franz Lang

**Affiliations:** 1 Department of Biochemistry and Molecular Biology and Centre for Comparative Genomics and Evolutionary Bioinformatics, Sir Charles Tupper Medical Building, Dalhousie University, 5850 College Street, Halifax, Nova Scotia B3H 4R2 Canada; 2 Département de Biochimie and Robert-Cedergren Center for Bioinformatics and Genomics, Université de Montréal, Montréal, QC Canada; 3 School of Biosciences, University of Birmingham, Edgbaston, Birmingham, B15 2TT UK; 4 Department of Biology and Ecology, Faculty of Science, University of Ostrava, Ostrava, Czech Republic; 5 Institute of Evolutionary Biology (CSIC-UPF), Barcelona, Spain; 6 Current address: Institute of Molecular Genetics, Academy of Sciences of the Czech Republic, Prague, Czech Republic

**Keywords:** Mitochondrion, Mitochondrial genome, Mitochondrial proteome, Mitochondrial evolution, Protist, Jakobids, *Andalucia godoyi*

## Abstract

**Background:**

Comparative analyses have indicated that the mitochondrion of the last eukaryotic common ancestor likely possessed all the key core structures and functions that are widely conserved throughout the domain Eucarya. To date, such studies have largely focused on animals, fungi, and land plants (primarily multicellular eukaryotes); relatively few mitochondrial proteomes from protists (primarily unicellular eukaryotic microbes) have been examined. To gauge the full extent of mitochondrial structural and functional complexity and to identify potential evolutionary trends in mitochondrial proteomes, more comprehensive explorations of phylogenetically diverse mitochondrial proteomes are required. In this regard, a key group is the jakobids, a clade of protists belonging to the eukaryotic supergroup Discoba, distinguished by having the most gene-rich and most bacteria-like mitochondrial genomes discovered to date.

**Results:**

In this study, we assembled the draft nuclear genome sequence for the jakobid *Andalucia godoyi* and used a comprehensive in silico approach to infer the nucleus-encoded portion of the mitochondrial proteome of this protist, identifying 864 candidate mitochondrial proteins. The *A. godoyi* mitochondrial proteome has a complexity that parallels that of other eukaryotes, while exhibiting an unusually large number of ancestral features that have been lost particularly in opisthokont (animal and fungal) mitochondria. Notably, we find no evidence that the *A. godoyi* nuclear genome has or had a gene encoding a single-subunit, T3/T7 bacteriophage-like RNA polymerase, which functions as the mitochondrial transcriptase in all eukaryotes except the jakobids.

**Conclusions:**

As genome and mitochondrial proteome data have become more widely available, a strikingly punctuate phylogenetic distribution of different mitochondrial components has been revealed, emphasizing that the pathways of mitochondrial proteome evolution are likely complex and lineage-specific. Unraveling this complexity will require comprehensive comparative analyses of mitochondrial proteomes from a phylogenetically broad range of eukaryotes, especially protists. The systematic in silico approach described here offers a valuable adjunct to direct proteomic analysis (e.g., via mass spectrometry), particularly in cases where the latter approach is constrained by sample limitation or other practical considerations.

## Background

In the evolutionary transition from endosymbiotic bacterium to integrated cellular organelle, the mitochondrion has undergone extraordinary changes [1]. Most of the genes encoded by the original endosymbiont genome have been lost or transferred to the host nuclear genome, with the result that contemporary mitochondrial genomes contain only a small and quite variable fraction of the endosymbiont’s genetic information. Genes encoded by mitochondrial DNA (mtDNA) are expressed via intra-organellar transcription and translation systems, some of whose components are mtDNA-encoded, but most of which are specified by the nucleus. Genes undergoing mitochondrion-to-nucleus re-location (endosymbiotic gene transfer or EGT) acquire nuclear expression signals to become active, their transcripts being translated on cytosolic ribosomes, and the resulting proteins are either targeted to and imported into the mitochondrion, or directed to and function in other parts of the cell. Coordinated expression of two different genomes is therefore required to form a functional mitochondrion.

Studies of the repertoire of proteins comprising the mitochondrion (mitochondrial proteome or mitoproteome) have revealed that this organelle is a structural and functional amalgam of proteins having different phylogenetic origins: an evolutionary mosaic [2]. Unexpectedly, only a small fraction, typically 10–20%, of mitochondrial proteins [3–5] has a clear affinity with *Alphaproteobacteria*, the bacterial class with which mitochondria share a specific common ancestor (see [6] and refs. therein). A larger fraction appears generically prokaryotic, but proteins in this category usually lack robust phylogenetic connections, and their origin remains controversial. Lateral gene transfer (LGT) from various bacterial groups to the closest relatives of mitochondria, either before or after the mitochondrial endosymbiosis, has been invoked to account for some of the non-alphaproteobacterial genes [7, 8], but the number of phylogenetically clear-cut examples of this type is limited. Another sizeable fraction of the mitoproteome is without clear phylogenetic adherence altogether, possibly due to a combination of low sequence conservation (i.e., rapid sequence evolution) and sequence changes resulting from EGT. This fraction of the mitoproteome also includes proteins that are found (i.e., are conserved) only within eukaryotes and are considered to have emerged or evolved specifically within eukaryotes, subsequent to the endosymbiosis event. Finally, in each eukaryote whose mitoproteome has been analyzed in detail to date, a significant fraction consists of species-specific proteins: proteins that in standard BLASTp searches have no significant sequence similarity to any described protein, including in close relatives (e.g., [9, 10]).

Comparative analyses of genome data have provided evidence that the last eukaryotic common ancestor (LECA) was already a complex organism, having virtually all of the defining features of a typical eukaryotic cell, including a fully functional mitochondrion [11]. However, only limited comprehensive investigation of the mitoproteome, involving direct mass spectrometric (MS) analysis of isolated mitochondria and studies of mitochondrial targeting, has so far been carried out and has until recently been restricted mostly to model (mainly multicellular) eukaryotes: animals [12–15]), fungi [16–18], and plants [19–22]. Few such studies have examined the mitoproteome in unicellular eukaryotes (protists), wherein most of the phylogenetic diversity of the domain Eucarya resides; these few include *Tetrahymena thermophila*, a ciliate protozoan [9], *Chlamydomonas reinhardtii*, a chlorophyte alga [23], *Trypanosoma brucei*, a kinetoplastid protozoan [24], and *Acanthamoeba castellanii*, an amoeboid protozoan [10, 25]. Studies that are broadly based phylogenetically are particularly important for tracing the evolutionary history of the mitochondrion and its proteome, for defining the critical conserved structures and functions, for highlighting important similarities and differences in the major eukaryotic lineages, and for ultimately trying to make sense of the substantial portion of the mitoproteome that is variable and without currently assigned function in different eukaryotes.

Here, we report a detailed and comprehensive in silico analysis of genome and transcriptome sequence data to infer candidate mitochondrial proteins for the flagellate protozoan *Andalucia godoyi*, a jakobid [26]. The jakobids, a protistan lineage belonging to the eukaryotic supergroup Discoba [27, 28], are distinguished by having the most gene-rich and most bacteria-like mitochondrial genomes discovered to date, with *A. godoyi* mtDNA encoding the most genes of any characterized mtDNA [29, 30]. Uniquely among eukaryotes, jakobid mitochondrial genomes encode subunits of a bacterial-type α_2_ββ′σ RNA polymerase (RNAP) [30, 31]; in contrast, all other eukaryotes investigated to date employ a single-subunit, nucleus-encoded, T3/T7 bacteriophage-like mitochondrial RNAP [32, 33].

These observations raise a number of questions that are addressed in the study reported here: Do the jakobids possess a nucleus-encoded phage-like RNAP in addition to the bacterial type encoded in the mitochondrion? Does the mitoproteome of *A. godoyi*, like its mtDNA, retain additional ancestral (bacteria-like) features that have been lost in other eukaryotes? Is the *A. godoyi* mitochondrion as structurally and functionally complex as the mitochondrion in multicellular eukaryotes? Here, we present an overview of the inferred *A. godoyi* mitoproteome, with particular reference to the human and yeast mitoproteomes, pointing out similarities and differences and drawing conclusions about the probable composition of various conserved complexes in the ancestral eukaryotes.

## Results

### Overview

For the current study, our choice of the jakobid protist, *A. godoyi*, was guided by several considerations. First, jakobids belong to a eukaryotic supergroup, Discoba, that is only distantly related to commonly studied eukaryotes (metazoans, fungi, plants). Within jakobids, *A. godoyi* represents an early emerging lineage. Among completely sequenced and fully characterized mitochondrial genomes, that of *A. godoyi* is the most gene-rich and bacteria-like, i.e., it appears to have changed the least from the ancestral mitochondrial genome present in LECA. It was reasonable, then, to expect that the nucleus-encoded portion of the mitochondrial proteome might display more ancestral characteristics than mitochondrial proteomes in other eukaryotes. Second, *A. godoyi* has full oxidative phosphorylation capacity, in contrast to the several anaerobic protists whose mitochondrial proteomes have been examined to date [1]. Third, the *A. godoyi* nuclear genome is surprisingly small (~ 20 Mb), displaying a compact and simple gene organization, with the vast majority of genes not interrupted by introns. From a practical perspective, these characteristics greatly aided and simplified accurate gene identification.

Any survey of this type is bound to generate both false positive and false negative results. In the removal of likely false positives, manual culling of bioinformatically predicted candidates mainly considered their predicted function: i.e., proteins were eliminated from consideration, even if they were robustly predicted to be mitochondrion-targeted, if they clearly function elsewhere in the cell. The retained predicted proteins formally remain mitochondrial candidates until their localization is verified by independent evidence (e.g., MS, in vivo targeting); nevertheless, we can be certain of the mitochondrial localization of a core sub-set of the predicted mitoproteome because the proteins in this group, e.g., components of the electron transport chain (ETC) and oxidative phosphorylation (OxPhos), only function in this organelle.

**Fig. 1:**
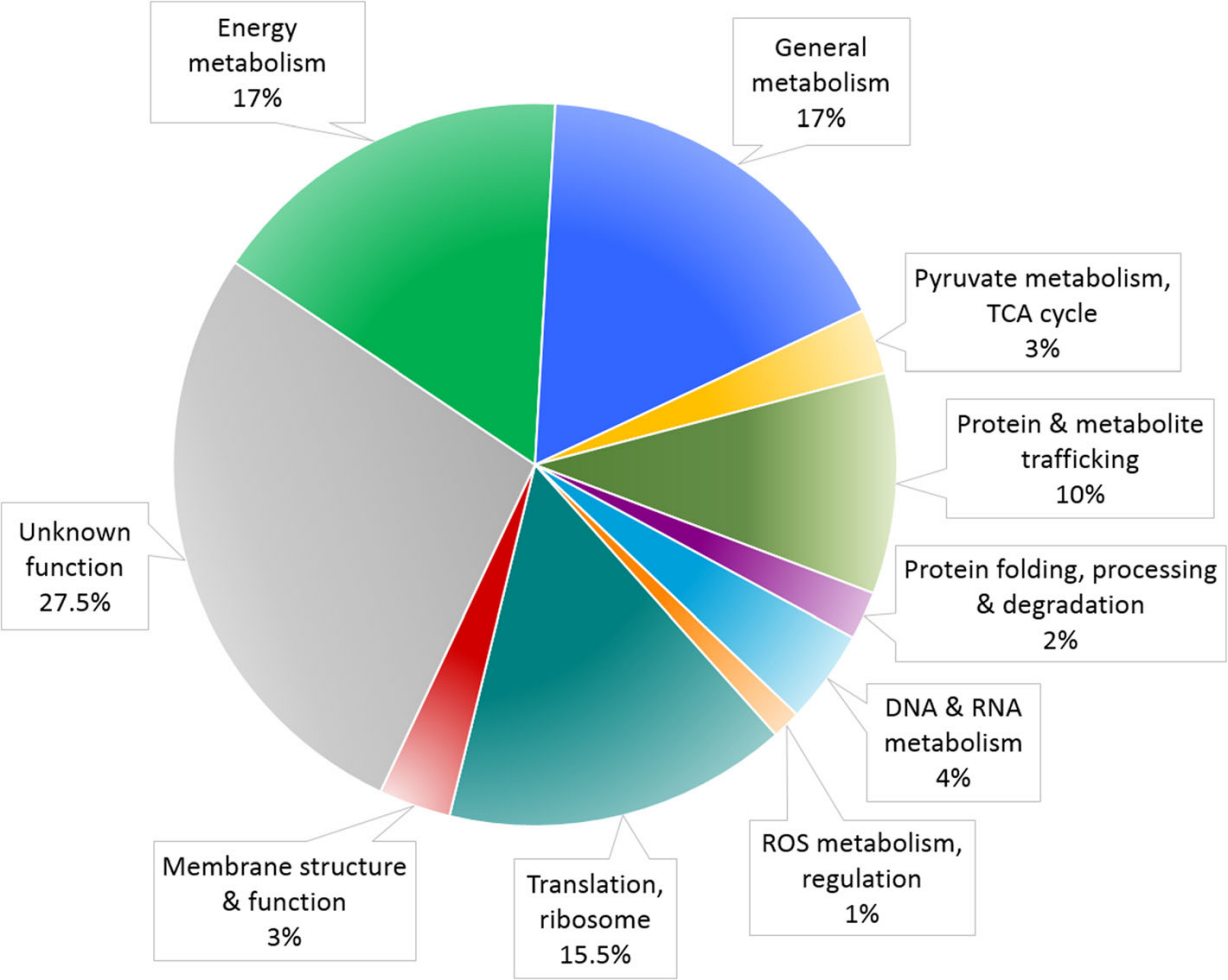
Functional categories comprising the *A. godoyi* mitoproteome

Our bioinformatic survey identified 864 nucleus-encoded candidate mitochondrial proteins, which together with 66 mtDNA-encoded proteins previously reported [30] predict a mitoproteome of 930 proteins (Additional file 1: Table S1). The largest functional categories, as expected [4, 34], are energy metabolism (17%), translation and ribosome structure and biogenesis (15.5%), and general metabolism (17%) (Fig. 1). Almost 1/3 of the predicted nucleus-encoded mitoproteome (27.5%) consists of uncharacterized proteins having no defined function; standard BLASTp against the NCBI non-redundant protein database indicated that 60% of proteins in this group have no putative conserved domain or have no significant similarity with known proteins.

Of the 864 nucleus-encoded proteins listed in Additional file 1: Table S1 (A) - (J), 61% have a strong or moderate probability of being mitochondrion-targeted according to our criteria. Some 138 proteins (16%) are predicted to lack an N-terminal mitochondrial targeting sequence (MTS); however, this group includes a substantial number that are known not to have such a targeting signal (e.g., some ETC and OxPhos components; mitochondrial carrier (MC) proteins). Notably, the mitochondrial targeting algorithms used here appear to work well with *A. godoyi* proteins, indicating an early emergence and universal application of the N-terminal mitochondrial targeting mechanism. For example, 13/13 enzymes comprising the tricarboxylic acid (TCA) cycle in *Andalucia*, 11/11 components of the iron-sulfur (Fe-S) cluster biosynthesis pathway, and 8/9 enzymes in the branched chain amino acid degradation pathway are predicted to have either a high (19) or moderate (13) probability of mitochondrial targeting, using the criteria established here (Additional file 2: Table S2).

A number of the genes characterized here were found to be sufficiently close to other genes in the genome that non-translated, overlapping portions of their transcripts caused the latter to be assembled into a single RNA-Seq contig. Such contigs faithfully mirrored the corresponding genomic sequence and organization of the genes in question. In a selection of 47 examples of this type, the intergenic spacer ranged in size from 44 to 691 nt (average 217 nt), with the transcriptional orientation of the two coding regions opposite in 38 instances, the same in 9. These examples of close gene packing are consistent with the relatively small size (~ 20 Mb) of the *A. godoyi* genome.

In *A. godoyi*, no introns are present in the vast majority of nuclear genes encoding candidate mitochondrial proteins. Of the 864 genes characterized, only 107 (12%) are inferred to have one or more introns (134 in total; all GT…AG), with most (82%) having only a single intron (Additional file 1: Table S1, Statistics). Among intron-containing genes, those encoding components of energy metabolism (B) are particularly enriched (30/122, or 25%, all single-intron). For 134 complete, verified introns, the average size is 151 bp (range 55–756).

## Functional categories (see Additional file 1: Table S1, tabs (A) to (J), for detailed entries)

### Pyruvate metabolism, TCA cycle (A)

All expected pyruvate dehydrogenase (PDH) subunits were identified (E1 α, β; E2; E3), as well as PDH-associated proteins pyruvate carboxylase and PDH kinase. Orthologs of genes encoding PDH phosphatase (PDP)—both catalytic and regulatory subunits—could not be detected (nor in the genome sequence of another discoban, *Naegleria gruberi*). However, Lander et al. [35] have recently described a Ca^2+^-stimulated mitochondrial PDP in *Trypanosoma cruzi*, and we have identified an *Andalucia* ortholog (ANDGO_04939.mRNA.1) that is very strongly predicted to be targeted to mitochondria. We suggest this protein as a possible PDP in *Andalucia*.

We identified five other pyruvate metabolism enzymes predicted to be mitochondrion-localized, including a pyruvate kinase (AgPK_1), the final enzyme in the glycolytic pathway. Ag_PK1 is bacteria-like and has a very strong MTS (all six targeting algorithms predict mitochondrial targeting with high probability). Although the predicted *A. godoyi* mitochondrial targeting could well be spurious, evidence for mitochondrial localization of pyruvate kinase has been reported for animals [36], apicomplexans [37], and stramenopiles [38–40]. *Andalucia* encodes two additional pyruvate kinases, evidently non-mitochondrial, one of which (AgPK_2) is a conventional cytosolic enzyme, the other (AgPK_3) with highest similarity to bacterial homologs. AgPK_1 and AgPK_3 affiliate with different bacterial clades (Additional file 3: Figure S1 (A)), with AgPK_1 also having a different evolutionary origin than other described mitochondrial pyruvate kinases [37, 39].

In contrast to its anaerobic jakobid relative, *Stygiella incarcerata* [41], *A. godoyi* appears to lack typical components of a hydrogen-producing fermentation system, such as pyruvate-ferredoxin oxidoreductase or [FeFe]hydrogenase and its associated maturases. However, like *Stygiella*, *Andalucia* does possess an organelle-targeted acetate:succinate CoA-transferase that might make it capable of the type of acetate-producing fermentation found in some anaerobically functioning mitochondria (reviewed in [42]).

All TCA cycle enzymes are present, with two distinct forms for isocitrate dehydrogenase, 2-oxoglutarate dehydrogenase E1, and succinyl-CoA synthetase β subunit.

### Energy metabolism (B)

#### Proteins of coupled electron transport-oxidative phosphorylation

##### Complex I (CI)

**Fig. 2:**
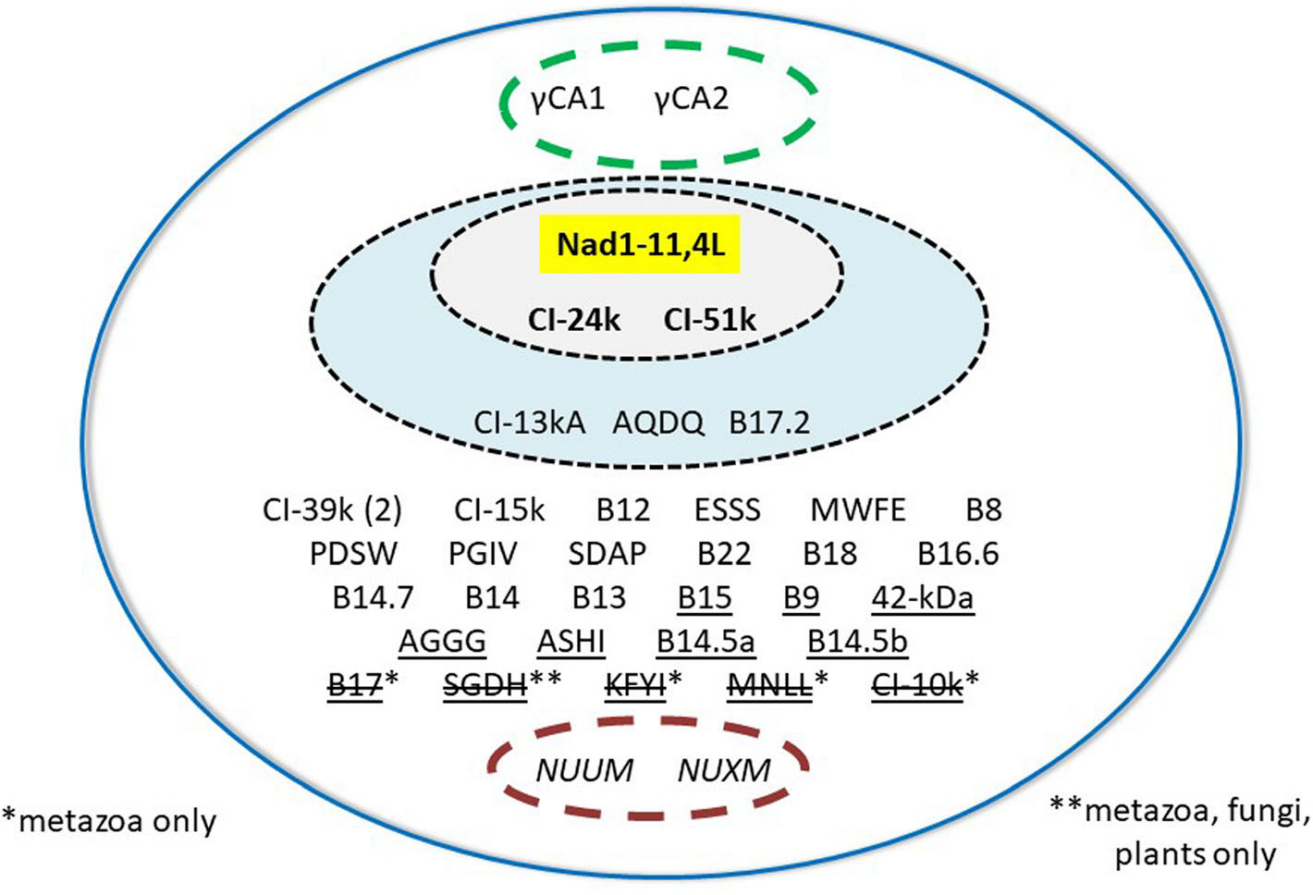
Composition of *A. godoyi* ETC complex I (CI). The figure lists the 44 CI subunits that have been identified in mammalian CI (see Table 1 and [46]), plus two γ carbonic anhydrase (γCA) subunits (dashed green oval) that are not present in mammalian or fungal CI, but are CI components in plants [53] and protists [54]. The blue-shaded oval encompasses the 17 subunits that comprise alphaproteobacterial (*Paracoccus denitrificans*) CI [52]. The smaller gray-shaded oval lists the 14 subunits that define the minimal active CI, with those subunits encoded in *A. godoyi* mtDNA highlighted in the yellow rectangle. Outside of the blue-shaded oval are listed 27 supernumerary, eukaryote-specific CI subunits, with those underlined considered by Brandt [44] to be metazoan-specific. With five exceptions (shown with strikethrough), all of these supernumerary subunits are present in *A. godoyi*, as well as the fungal-specific subunits NUUM and NUXM (dashed violet oval)

Mammalian CI was initially considered to comprise 45 subunits [43]; however, NDUFA4 (MLRQ), formerly thought to be a CI subunit [44], is actually a component of Complex IV (CIV) [45–47] (see Tables 1 and 2). Fourteen of the CI subunits are considered to represent a bacterial core, although three additional subunits previously designated as “supernumerary” (specific to eukaryotes) have since been identified in the alphaproteobacterium *Paracoccus denitrificans* [52]. These 17 subunits are all present in *A. godoyi* (Fig. 2), whose mitochondrial genome encodes almost twice as many of them as are specified by mammalian mtDNA (Table 1).

**Table 1:** Comparison of subunits in human (*Hsa*) and *A. godoyi* (*Ago*) ETC Complex I

Core subunits		“Eukaryote-specific subunits”		“Metazoan-specific subunits”	
*Hsa*	*Ago*	*Hsa*	*Ago*	*Hsa*	*Ago*
**MT-ND1**	**Nad1**	NDUFAB1	SDAP	NDUFA3	B9
**MT-ND2**	**Nad2**	NDUFA1	MWFE	NDUFA7	B14.5a
**MT-ND3**	**Nad3**	NDUFA2	B8	NDUFA10	CI-42k
**MT-ND4**	**Nad4**	NDUFA5	B13	NDUFB1	n.d.
**MT-ND4L**	**Nad4L**	NDUFA6	B14	NDUFB2	AGGG
**MT-ND5**	**Nad5**	NDUFA8	PGIV	NDUFB4	B15
**MT-ND6**	**Nad6**	NDUFA9	CI-39k^a^	NDUFB5	n.d.
NDUFS2	**Nad7**	NDUFA11	B14.7	NDUFB6	n.d.
NDUFS8	**Nad8**	NDUFA13	B16.6	NDUFB8	ASHI
NDUFS3	**Nad9**	NDUFB3	B12	NDUFC1	n.d.
NDUFS7	**Nad10**	NDUFB7	B18	NDUFC2	B14.5b
NDUFS1	**Nad11**	NDUFB9	B22	NDUFV3	n.d.
NDUFV1	CI-51k	NDUFB10	PDSW	n.d.	NUXM^b^
NDUFV2	CI-24k	NDUFB11	ESSS	n.d.	NUUM^b^
NDUFA12	B17.2	NDUFS5	CI-15k		
NDUFS4	AQDQ				
NDUFS6	CI-13kA				

Subunits shown in bold are encoded in mtDNA. n.d., not detected. *A. godoyi* nomenclature is based on that of bovine CI [44]

^a^We identified two distinct isoforms of this subunit in *Andalucia*, encoded by separate genes

^b^NUXM and NUUM (identified in fungi) were initially considered to be distant homologs of the human subunits NDUFB1 and NDUFC1, respectively [48], but more recently this conclusion has been disputed [49]

**Table 2:** Comparison of human (*Hsa*) and *A. godoyi* (*Ago*) CII, CIII, CIV, and CV subunits

Complex II		Complex III		Complex IV		Complex V	
*Hsa*	*Ago*	*Hsa*	*Ago*	*Hsa*	*Ago*^a^	*Hsa*	*Ago*
						*F*_*1*_ *subunits*	
SDHA	Sdh1	UQCRC1	Qcr1	**COX1**	**Cox1**	ATP5F1A	**Atp1 (AtpA)**
SDHB	**Sdh2**	UQCRC2	Qcr2	**COX2**	**Cox2**	ATP5F1B	**Atp2 (AtpD)**
SDHC	**Sdh3**	**MT-CYB**	**Qcr3 (Cob)**	**COX3**	**Cox3**	ATP5F1C	**Atp3 (AtpG)**
SDHD	**Sdh4**	CYC1	Qcr4 (Cyc1)	COX4	Cox5	ATP5F1D	**Atp16 (AtpC)**
n.d.	Sdh5	UQCRFS1	Qcr5	COX5A^c^	n.d. (Cox6)	ATP5F1E	Atp15
		UQCRH	Qcr6	COX5B	Cox4		
		UQCRB	Qcr7	COX6A	Cox13	*F*_*o*_ *subunits*	
		UQCRQ	Qcr8	COX6B	Cox12	**MT-ATP6**	**Atp6 (AtpB)**
		UQCR10	Qcr9	COX6C^c^	n.d. (Cox9)	ATP5PB	**Atp4 (Orf25 / Ymf39)**
		UQCR11	n.d.	COX7A	Cox7	ATP5MC1/2/3	**Atp9 (AtpE)**
		Subunit 9^b^		COX7B^d^	n.d. (—)	ATP5PD	Atp7
				COX7C	n.d. (Cox8)	ATP5ME	n.d.
				COX8	Cox8h^e^ (—)	ATP5MF	Atp17
				COXFA4^f^	CoxFA4	ATP5MG	n.d.
						ATP5PF	F6
						ATP5PO	Atp5 (AtpH)
						**MT-ATP8**	**Atp8 (OrfB / Ymf19)**
						ATP5IF1	Inh1
						ATP5S^c^	n.d.

Subunits shown in bold are encoded in mtDNA. n.d., not detected

^a^Yeast (*Saccharomyces cerevisiae*) nomenclature

^b^8-kDa cleavage product from the N-terminus of the Rieske protein (UQCRFS1)

^c^Not identified outside of opisthokonts

^d^Not identified outside of animals

^e^Retrieved with *Arabidopsis* CoxVc (At2g47380), considered to be the counterpart of mammalian CoxVIII (see [50])

^f^NDUFA4 (MLRQ), previously considered to be a Complex I subunit [51]

Initially, the supernumerary proteins found in mammalian CI were divided into two classes, “eukaryote-specific” and “metazoan-specific” [44]. We identified all 15 “eukaryote-specific” CI proteins in *A. godoyi* (Table 1). Of 12 subunits designated “metazoan-specific,” 7 were found (the same collection as in the *A. castellanii* mitochondrial proteome [25]), with two distinct copies of the CI-39k/NDUFA9 subunit identified. Cardol [48] did not report subunit B15/NDUFB4 in discobans, but we find that it is present in both *A. godoyi* and *N. gruberi*.

Four subunits (KFYI/NDUFC1, MNLL/NDUFB1, CI-10 k/NDUFV3 and B17/NDUFB6) have so far not been found outside of Holozoa (metazoans and their unicellular relatives), whereas subunit SGDH/NDUFB5 has to date been identified only in animals, fungi, and possibly plants [48]. Failure to identify these five subunits in our study is therefore not unexpected.

Two “fungal-specific” subunits (NUUM and NUXM; also present in the *A. castellanii* mitoproteome) were retrieved here. Cardol [48] suggested that these proteins are distant homologs of metazoan subunits NDUFC1 and NDUFB1, respectively; however, this conclusion has been disputed [49]. Our analysis therefore suggests that 39 of the 44 proteins comprising mammalian CI were likely present in the LECA mitoproteome, as well as orthologs of fungal NUUM and NUXM, which appear to have been lost early in metazoan evolution (Fig. 2 and Table 1).

Finally, we identified two γ carbonic anhydrase CI subunits, which are not found in opisthokont CI but are otherwise widely distributed among eukaryotes [53–55] (Fig. 2). Again, these two proteins likely represent ancestral features of CI.

Some 20 proteins have been implicated as assembly factors for mammalian CI, with 14 known to participate in the formation of different intermediate assembly modules [56–58]. Orthologs of 9 of these 20 factors were identified here (Table 3).

**Table 3:** Factors involved in the assembly of ETC Complexes I–V

Complex I		Complex II		Complex III		Complex IV		Complex V		
*Hsa*	*Ago*	*Hsa*	*Ago*	*Hsa*	*Ago*^b^	*Hsa*	*Ago*^b^	*Hsa*	*Ago*	*Sce*
NUBPL	NUBPL	SDHAF1	SdhAF1	MZM1L	LYRM7	CMC1	Cmc1	ATPAF1	Atp11	
NDUFAF2	NDUFAF2	SDHAF2	SdhAF2	BCS1L	n.d.	CMC2	Cmc2	ATPAF2	Atp12	
NDUFAF3	NDUFAF3	SDHAF3	SdhAF3	UQCC1	Cbp3	COA1	n.d.	ATP23	Atp23	
NDUFAF4	n.d.	SDHAF4	SdhAF4	UQCC2	n.d.	(COA2^c^)	n.d.	TMEM70	n.d.	
NDUFAF5	NDUFAF5			UQCC3	n.d.	COA3	n.d.			
NDUFAF6	NDUFAF6			TTC19	n.d.	COA4	Coa4		Atp10	Atp10
NDUFAF7	NDUFAF7					COA5	Coa5		Nca2	Nca2
NDUFAF8	n.d.			–	**CcmA**	COA6	Coa6			
*NDUFAF1*^*a*^	NDUFAF1			–	**CcmB**	COA7	n.d.			
*TIMMDC1*	n.d.			–	**CcmC**	COX10	Cox10			
*ECSIT*	n.d.			–	CcmD	COX11	**Cox11**			
*ACAD9*	n.d.			–	CcmE	COX14	n.d.			
*TMEM126B*	n.d.			–	**CcmF**	COX15	**Cox15**			
TMEM186	n.d.			–	CcmG	COX16	Cox16			
FOXRED1	n.d.			–	CcmH	COX17	Cox17			
ATP5SL	n.d.			–	CcmI	COX18	Cox18			
DMAC1	DMAC1					COX19	Cox19			
COA1	n.d.					COX20	Cox20			
TMEM70	n.d.					COX23	Cox23			
AIF	AIF^e^					PET100	Pet100F^d^			
						PET117	Pet117			
						TACO1	Taco1			
						FASTKD2	n.d.			
						SCO1 / 2	Sco			
						SURF1	Surf1/Shy1			

See HGNC (mitochondrial respiratory chain complex assembly factors) [59], Guerrero-Castillo et al. [56], and Signes and Fernandez-Vizarra [58]. n.d., not detected

^a^Genes set in italics are members of the Mitochondrial Complex I Assembly (MCIA) complex (see [60])

^b^Genes set in bold are encoded in the *A. godoyi* mitochondrial genome

^c^Exclusively fungal; no human ortholog

^d^Fungal version

^e^Listed under (I) ROS METABOLISM, REGULATION in Additional file 1: Table S1

We retrieved sequences of 13 proteins of unassigned function belonging to the Complex1_LYR-like superfamily (10 having a PF05347 domain, 2 having PF13223). All are predicted to be targeted to mitochondria, most with medium or high probability. Additional Complex1_LYR proteins include CI subunits NDUFA6 and NDUFB9 as well as assembly factors for Complexes II (SdhAF1, SdhAF3) and III (MZM1L/LYRM7), electron transfer flavoprotein regulatory factor 1 (ETFRF1/LYRM5), and a protein involved in Fe-S cluster biosynthesis (Isd11); see below. On this basis, some of the unassigned Complex1_LYR proteins could well have a role in the assembly or regulation of ETC-OxPhos or other mitochondrial complexes.

##### Complex II (CII)

In mammals, none of the four proteins comprising CII is encoded in the mitochondrial genome, whereas three (Sdh2-4) are mtDNA-encoded in *A. godoyi*, the fourth (Sdh1) being nucleus-encoded (Table 2). In some other discobans (trypanosomatids and euglenids), the Sdh2 gene has been transferred to the nucleus, but in two pieces [61, 62].

Complex II in plants [63, 64] and trypanosomatids [62] has been reported to contain additional lineage-specific subunits, one of which (an ortholog of plant Sdh5) we identified here (Table 2). Together with the recent identification of homologs of plant Sdh5, Sdh6, and Sdh7 outside of land plants [65], this result suggests that CII in LECA may have been more complex than its four-subunit bacterial counterpart, implying that the additional subunits represent a retained ancestral eukaryotic trait, rather than resulting from lineage-specific additions as is generally assumed. This observation would imply that the four-subunit CII in opisthokonts resulted from loss of subunits originally present in LECA CII (essentially a reversion to the primitive bacterial composition). This scenario has been argued in the case of CI, which in opisthokonts specifically lacks γ-type carbonic anhydrase subunits that are otherwise widely distributed among other eukaryotes [54].

Four CII assembly factors (SdhAF1-AF4) identified in mammals are also present in *A. godoyi* (Table 3).

##### Complex III (CIII)

Mammalian CIII comprises 10 structural proteins, with only apocytochrome *b* (Cob) encoded by mtDNA. *A. godoyi* CIII appears to contain nine subunits; only a Qcr10 homolog (human UQCR11) could not be identified (Table 2). Marx et al. [66] reported that biochemically isolated CIII of another jakobid, *Seculamonas ecuadoriensis*, is composed of at least eight subunits between 6 and 46 kDa in size, including two large core subunits (the matrix processing peptidase α and β subunits; Qcr2 and Qcr1, respectively) and the three respiratory subunits (Cob, cytochrome *c*_1_ and the Rieske Fe-S protein).

In addition to cytochrome *c*_1_ (Cyc1), we identified cytochrome *c*_2_ (Cyc7), which is also found in *A. castellanii* and *N. gruberi*, and widely distributed in animals, fungi, and plants. In yeast, transcription of CYC7 is repressed under aerobic conditions and induced at low oxygen concentrations [67, 68].

Seven proteins belonging to the Ccm cytochrome *c*-type biogenesis pathway were identified in our analysis (Table 3). The Ccm system (also called System I) is found in Gram-negative bacteria, archaea, and plant and protist mitochondria [1, 69]. In *A. godoyi*, Ccm subunits A, B, C, and F are encoded by mtDNA whereas subunits D, E, and H are nucleus-encoded. We did not identify subunits G and I, the remaining two subunits comprising the bacterial Ccm system; these particular subunits have also not been found in the plant mitochondrial Ccm system [69]. Notably, CcmD has been reported to be absent in plant mitochondria [69], but a hidden Markov model (HMM) search retrieved a putative *A. godoyi* homolog (ANDGO_06663.mRNA.1).

Five proteins conserved between mammals and fungi have been identified as assembly factors for CIII [58] (Table 3). We identified only two of these, MZM1L/LYRM7 and UQCC1/Cbp3, in *Andalucia*. Because CIII assembly factors UQCC2 and UQCC3 are poorly conserved between fungi and metazoa (homology is barely detectable, even with HHpred), our inability to identify them in *Andalucia* might be linked to a low overall level of sequence conservation. We could not retrieve an *Andalucia* BCS1 domain-containing protein, although BCS1_N and AAA domain-containing proteins were readily identified in *Stygiella incarcerata* (ANM86792.1), *N. gruberi* (XP 002672539.1, XP_002674781.1), and *A. castellanii* (XP_004336352.1, XP_004334174.1).

##### Complex IV (CIV)

Mammalian CIV is composed of 14 subunits [46], including COXFA4 [51], originally named NDUFA4 (MLRQ) and, as noted earlier, initially considered to be a CI subunit [44]. We identified 10 CIV subunits, including CoxFA4 (Table 2), with the same 3 proteins (Cox1, Cox2, Cox3) being encoded in both human and *A. godoyi* mtDNA. Of the 4 subunits that we did not identify in *Andalucia*, only COX7C (Pfam PF02935) has evident homologs outside of opisthokonts. The remaining 3 subunits may therefore represent lineage-specific components.

We identified 19 orthologs of 25 factors implicated in the assembly of CIV, two of which (Cox11 and Cox15) are encoded in *Andalucia* mtDNA (Table 3). Among jakobids, a mtDNA-encoded Cox15 (heme *a* synthase) is found only in *Andalucia*, the result of an apparent bacterial LGT event into the *Andalucia* mitochondrial genome [30, 70]. Cox15 interacts with structural protein Cox13 during the late stages of CIV assembly and has recently been shown to additionally interact with the CIII protein Cor1 (Qcr1) in higher-order supercomplexes [71]. Notably, we identified orthologs of all 7 CIV assembly factors required for Cox2 polypeptide maturation and Cu_A_ site formation [72]. One of these (Cox18) is encoded by duplicate, identical, tandemly arrayed genes in the *A. godoyi* nuclear genome.

In yeast (*S. cerevisiae*), two PF04588 HIG_1_N domain-containing proteins, Rcf1 and Rcf2, have been shown to play a role in modulation of the activity CIV, as well as its assembly [73]. We identified an ortholog of Rcf1 but not Rcf2. Although *Andalucia* Rcf1 is not predicted to be targeted to mitochondria, neither is yeast Rcf2 nor the human homolog, HIG1 domain family member 2A.

Of the seven CIV assembly factors that we did not retrieve here, only two (COA1 and COX14) have identified homologs outside of animals, fungi, and plants.

##### Complex V (CV)

Mammalian CV comprises 15 subunits, 5 F_1_ and 10 F_o_, two of which are encoded in mtDNA [74, 75]. We identified orthologs of 13 of these subunits in *A. godoyi*, with eight being encoded in mtDNA (Table 2). The ATPase inhibitor Inh1 (mammalian ATP5IF1) is also present in *A. godoyi*, but another regulatory protein, Factor B (ATP5S), was not retrieved. Notably, we did not find homologs of mammalian ATP5ME and ATP5MG (subunits e and g, respectively), even when using HMM searches. These two subunits are involved in CV dimerization: they do not form dimer contacts but enable dimer formation by inducing a curvature of ~ 100° in the mitochondrial inner membrane (MIM) [76].

Five proteins identified as CV assembly factors in mammalian or yeast mitochondria were also identified in our analysis (Table 3).

#### Other respiratory proteins

A branched mitochondrial respiratory chain appears to be present in *A. godoyi*, as indicated by our finding of two alternative oxidase (AOX) isoforms and a rotenone-insensitive NADH dehydrogenase (NDE2). The heterodimeric electron transfer flavoprotein ETF (subunits α and β) and an associated ETF-ubiquinone oxidoreductase are also present.

In mammals, mitochondrial glycerol-3-phosphate dehydrogenase (mGPDH) plays an important role in cellular bioenergetics, linking carbohydrate and lipid metabolism and constituting a major contributor of electrons to the mitochondrial electron transport chain [77]. In humans, two forms of GPDH, GPD1 (cytosolic) and GPD2 (mitochondrial), participate in a glycerophosphate shuttle between the cytosol and mitochondria, thereby maintaining an NAD^+^/NADH ratio that is optimal for metabolism. *Andalucia* does not contain a GPD1 homolog but does encode a GPD2 ortholog, although the latter is not predicted to have a MTS.

### DNA and RNA metabolism (C)

#### DNA replication and repair

**Fig. 3:**
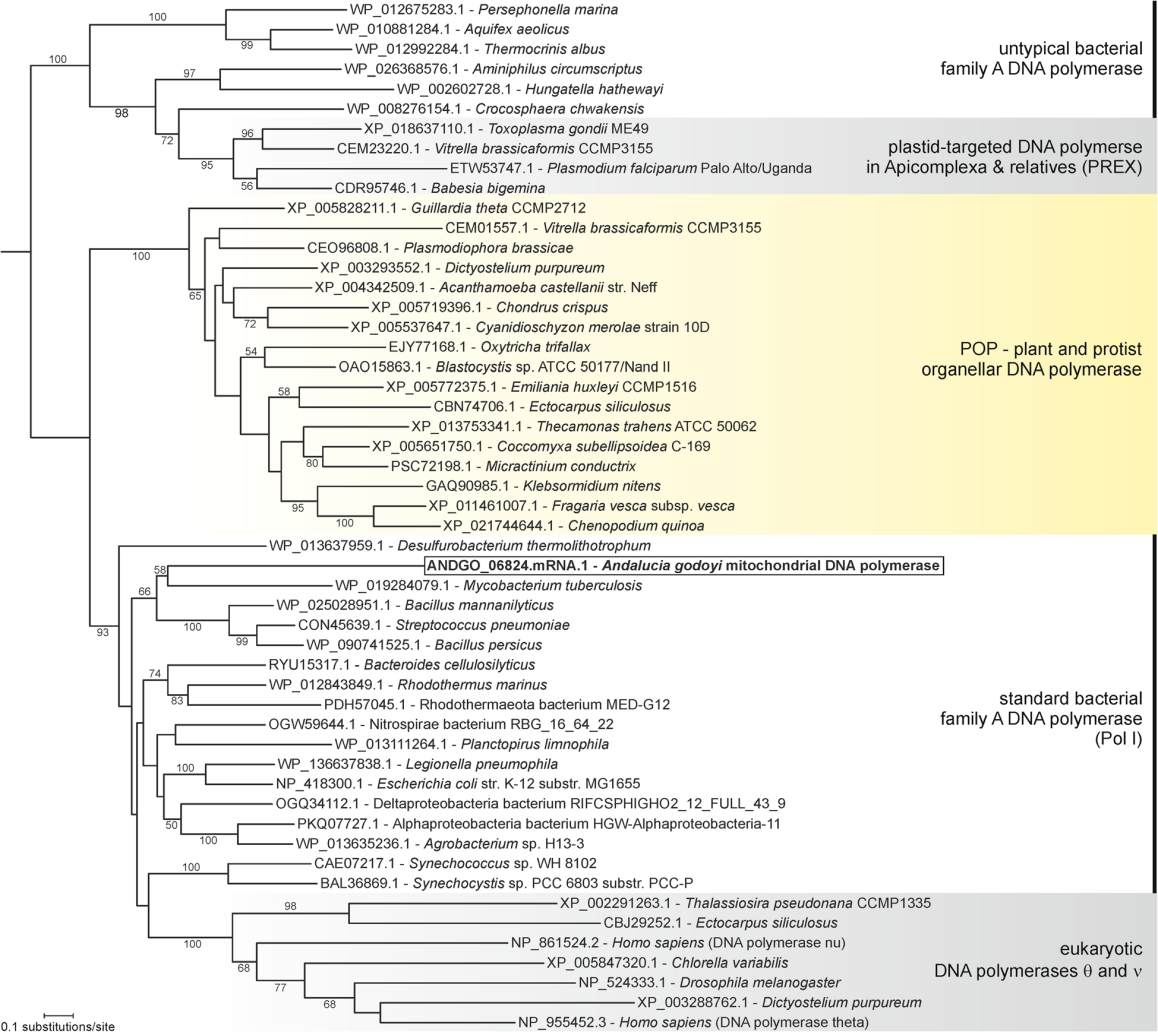
Phylogenetic analysis of family A DNA polymerases. The arbitrarily rooted ML tree was inferred using RAxML (PROTGAMMALG model) based on an alignment of 533 amino acid positions. Branch support values (rapid bootstraps) ≥ 51% are shown. Note the position of the mitochondrion-targeted *A. godoyi* DNA polymerase, branching off among standard bacterial family A DNA polymerases (Pol I) and separately from the organellar (mitochondrial or dually targeted) DNA polymerases widespread in plants and protists (the POP group)

Unlike animals and fungi, which utilize a T3/T7 bacteriophage-like DNA polymerase (DNAP) γ for mtDNA replication [33, 78], eukaryotes other than opisthokonts use a bacterial Pol A-like (Pol I) DNAP. This enzyme was given the name “plant organellar DNA polymerase” (POP) by Moriyama et al. [79, 80] but has since been identified also in protists [33, 81]. It constitutes a distinct phylogenetic group without close relatives and hence of unclear origin [33, 79–81]. In bacteria, Pol I plays an essential role in replication through its 5′-to-3′ exonuclease activity, which together with the 5′-to-3′ polymerase activity catalyzes the removal of RNA primers from replication intermediates. Additionally, this concerted activity (nick translation) functions in DNA repair. The *Andalucia* mitoproteome also contains a Pol A-like DNAP, but interestingly, it is unrelated to enzymes of the POP group and instead branches among the standard bacterial Pol I polymerases (Fig. 3). In addition, the N-terminal portion of this protein appears to have only a remnant 5′-to-3′ exonuclease domain. We did identify a separate mitochondrion-targeted 5′-to-3′ exonuclease, which we suggest is likely the functional equivalent of the covalently integrated, N-terminal 5′-to-3′ exonuclease found in a bacterial Pol I and in POP proteins.

The bacterial replisome comprises DnaA, DnaB, and DnaC replication initiation factors, a DnaG primase, and a replicative holoenzyme consisting of a family C DNAP (Pol III), assembled in a complex with nine other proteins [82]. We could not identify counterparts of any of these proteins, with the single exception of a bacterial-type DEDDh 3′-5′ exonuclease domain family protein, which is homologous to Pol III holoenzyme subunit ε (DnaQ).

Other candidate proteins implicated in *A. godoyi* mitochondrial genome replication include Twinkle, the primase-helicase that is widely distributed among eukaryotes [83], several other helicases, RNase H1, DNA topoisomerases II and III, and an ATP-dependent DNA ligase. It is evident that major re-modeling of the replication machinery in mitochondria occurred prior to the emergence of LECA, with a further major change (from a Pol A-family DNAP to a T3/T7 bacteriophage-like γ DNAP) in opisthokonts.

**Fig. 4:**
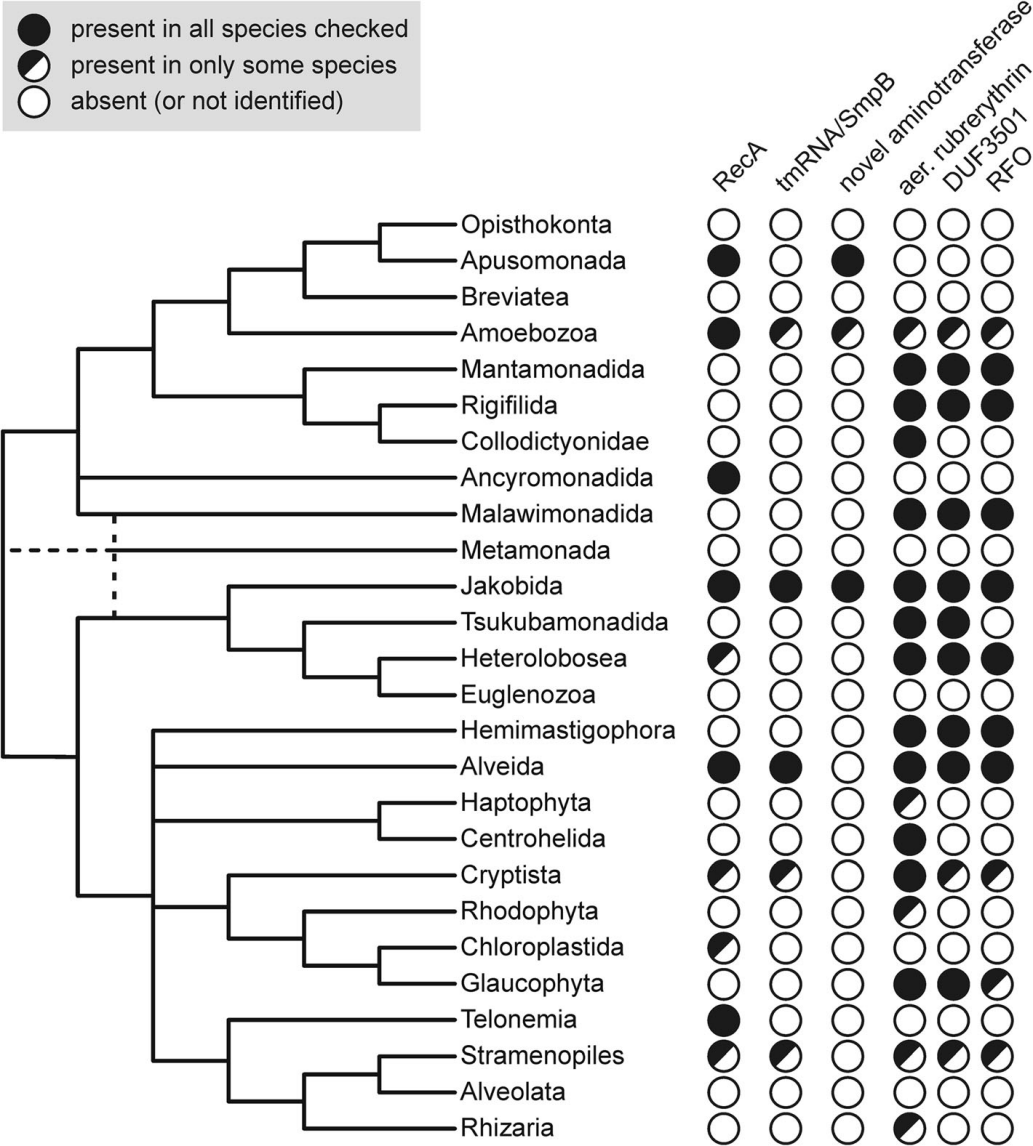
Distribution of selected mitochondrial components (proteins, non-coding RNA) in eukaryotes. The schematic tree was drawn as a consensus of recent phylogenomic analyses [28, 85–88]; the position of Metamonada remains unresolved (possible affinities indicated by dashed lines). RecA sequence IDs are provided in Additional file 4: Table S3 (A), details on tmRNA and SmpB sequences are listed in Additional file 4: Table S3 (B). “Novel aminotransferase” corresponds to a member of the DegT/DnrJ/EryC1/StrS family patchily distributed in a few eukaryotes (see text and Additional file 5: Figure S2). The three proteins constituting the aerobic-type rubrerythrin system (aer. rubrerythrin, DUF3501, RFO) exhibit a highly coherent distribution, with the latter two proteins found only in species that encode an aerobic-type rubrerythrin (see Additional file 4: Table S3 (C) for a detailed view)

We also identified at least eight proteins likely involved in mitochondrial DNA recombination and/or repair, notably including an ortholog of the previously identified alphaproteobacteria-affiliated RecA proteins occurring in mitochondria of certain protists and plants (Additional file 3: Figure S1 (B)). The phylogenetic distribution and affinity of mitochondrial RecA has previously been studied by Hofstatter et al. [84], who concluded that the protein has likely been secondarily lost in Discoba. Our identification here of mitochondrion-targeted RecA in *Andalucia* and some heteroloboseans (Additional file 4: Table S3 (A)) revises this notion. Indeed, the distribution of mitochondrial RecA in eukaryotes is broader than reported previously (Fig. 4), consistent with this protein being a primitive mitochondrial characteristic, contributed by the mitochondrial endosymbiont.

#### Transcription and RNA processing

One of the most striking features of *Andalucia* and other jakobid mitochondrial genomes is the presence of four genes (*rpoA*, *rpoB*, *rpoC*, *rpoD*) that together specify a multi-component α_2_ββ′σ bacterial-type RNAP [30]. A mtDNA-encoded RNAP of this type is so far unique to the jakobids: all other eukaryotes studied to date, including non-jakobid members of Discoba, employ a nucleus-encoded, single-subunit, bacteriophage T3/T7-like enzyme as the mitochondrial RNAP (RpoY [33]). We would expect an α_2_ββ′σ-type RNAP to have been present in the alphaproteobacterial ancestor of mitochondria (ancestral state), so the origin of the phage-type RNAP and the evolutionary transition from an ancestral (bacterial) to a derived (phage) type of mitochondrial transcription system are key unanswered questions.

We tested the possibility that the *Andalucia* nuclear genome might encode an RpoY-type RNAP that is imported into mitochondria to participate in gene expression in the organelle in conjunction with the organelle-encoded RNAP, as occurs in land plant chloroplasts [32]. Accordingly, we carried out searches of both the *Andalucia* genome and transcriptome data using various eukaryotic and phage RpoY sequences. Because the C-terminal portion, in particular, of the RpoY sequence is well conserved, and because both the *Andalucia* genome and transcriptome have been deeply sequenced, such a gene, if present, should have been retrieved readily. However, we found no evidence of an RpoY-type sequence, and the same negative result was obtained with four other sequenced jakobid genomes (unpublished observations). We conclude, therefore, that the mtDNA-encoded RNAP is likely the sole mitochondrial transcriptase in *Andalucia*. Notably, the transcription apparatus functioning in the *Andalucia* mitochondrion may have retained additional eubacterial traits, as suggested by our identification here of a divergent mitochondrion-targeted GreA/GreB transcription elongation homolog. In bacteria, GreA/GreB cooperates with RNA polymerase to enhance the enzyme’s intrinsic transcript cleavage activity [89], thereby helping to rescue stalled transcription complexes.

We also searched for homologs of mitochondrial transcription factors (mtTF) A and B, which function together with the phage-like RNAP in mitochondrial transcription in opisthokonts [90]. We found no evidence of a mtTFA homolog, whereas a mtTFB query retrieved two *Andalucia* homologs, AgDMT1 and AgDMT2, both of which have a dimethyltransferase (DMT) domain, as do both yeast [91] and human [92] mitochondrial mtTFBs. AgDMT1 is (weakly) predicted to be targeted to mitochondria, and phylogenetic analysis shows that this protein is part of a broad clade of mtTFB1 and alphaproteobacterial DMT sequences identified by Harris and Goldman [93]. The presence of a characteristic motif found in conventional DMTs (PPPKV, positions 189-193 in the *Andalucia* protein) strongly suggests that the *Andalucia* protein functions as a DMT, as do some mtTFB1s [93]. In other ribosomal systems, both prokaryotic and eukaryotic cytosol as well as organelles, this protein is responsible for dimethylation of adjacent A residues in the 3′-terminal helix of small subunit (SSU) rRNA, corresponding to positions A1457 and A1458 in the *Andalucia* mitochondrial counterpart.

We found no evidence of a mitochondrial ρ-type transcription termination factor, although this protein has recently been identified in a plastid system [94], which also employs a multi-subunit bacterial-type RNAP. Conversely, we found a clear mitochondrial transcription termination factor (mTERF) ortholog in *Andalucia*, as well as an additional protein having an mTERF domain. We also identified several proteins likely involved in various aspects of mitochondrial RNA metabolism, including orthologs of ribonuclease III (rRNA maturation), endonuclease LCL3, RNA helicase SUPV3L1 (a component of mtEXO, the mitochondrial degradosome complex), protein PET127 (5′-end mRNA-processing), ribonuclease Z (tRNA 3′-end maturation), and tRNA CCA nucleotidyltransferase. The latter sequence displays characteristics of the ancestral eCCA enzyme, as defined by Betat et al. [95], rather than the animal-specific aCCA version that these authors infer was acquired more recently by LGT from *Alphaproteobacteria* at the origin of Holozoa.

*Andalucia* mtDNA encodes a bacterial-type RNase P RNA [30], and we retrieved a mitochondrion-targeted ortholog of RnpA, the protein component of bacterial RNase P. The putative *Andalucia* protein is highly diverged compared to its bacterial counterparts but it does display the specific RnpA domain (COG0594) that contains most of the conserved residues that have been implicated in RnpA function [96]. Bacterial-type RnpA orthologs have previously been reported in several prasinophyte algae [97], although their subcellular localization (mitochondrion or plastid) has not been established.

#### Pentatricopeptide repeat (PPR) proteins

Proteins having a PPR motif [98] have been implicated in various aspects of organelle RNA metabolism, particularly C-to-U RNA editing in land plant mitochondria and chloroplasts [99]. We identified six PPR proteins in *Andalucia*; all of them are predicted to be targeted to mitochondria, five of them strongly so. None of these proteins contains the DYW (cytidine deaminase) domain characteristic of PPR proteins implicated in organellar RNA editing [100], in agreement with the lack of evidence of RNA editing in *Andalucia* mitochondria. We assume these PPR proteins are involved in other RNA processing pathways (see [101]).

### Translation, ribosome (D)

#### Translation factors

As expected, the mitochondrial translation system of *Andalucia* appears to utilize bacterial-type initiation factors IF2 and IF3 (an IF1 homolog was not retrieved); elongation factors EF-G1, EF-Tu (mtDNA-encoded), EF-Ts, EF-P, and GUF1/LepA/EF4; and six peptide chain release/ribosome recycling factors: EF-G2, mtRF1, mtRF2, human C12orf65-like peptide release factor, ribosome silencing factor RsfS, and ribosome recycling factor (RRF). Mitochondrial EF-G1 and EF-G2 separately carry out the two functions of bacterial EF-G, namely translocation (EF-G1) and ribosome recycling in conjunction with mitochondrial RRF (EF-G2) [102].

Peptidyl-tRNA hydrolases (PTHs) play a critical role in translation systems by salvaging tRNAs from peptidyl-tRNAs released at all stages of protein synthesis [103]. *Andalucia* encodes four PTHs: two belonging to class Pth1 (Pfam domain Pept_tRNA_hydro) and two members of class Pth2 (Pfam domain PTH2). All lack a MTS, including AgPth2_1 (ANDGO_07382), which in BLASTp searches retrieves homologs annotated as “mitochondrial.” Orthologs of AgPth2_1 are widespread in Archaea and Eucarya but are not found in bacteria [104]. Like AgPth2_1, none of the eukaryotic orthologs (including human and yeast) has a MTS according to our targeting criteria; nevertheless, there is direct experimental evidence that the human ortholog localizes to and functions in mitochondria [105]. On that basis, we conclude that AgPth2_1 is likely a mitochondrial enzyme.

In bacteria, several systems function in quality control by monitoring protein synthesis and recycling stalled translation complexes. These systems include the tmRNA-SmpB complex as well as ArfA and ArfB [106, 107]. The *Andalucia* mitochondrial genome encodes a structurally reduced tmRNA species [30, 108], and we retrieved the corresponding partner protein, an alphaproteobacteria-affiliated SmpB (Additional file 3: Figure S1 (C)). The peptide release factor C12orf65, noted above, is probably derived from eukaryotic release factor ICT1, which in turn is a homolog of bacterial ArfB [109, 110].

A mtDNA-encoded tmRNA has also been reported in oomycetes [111] and in *Palpitomonas bilix*, a heterotrophic microeukaryote related to cryptophytes [112]. We have identified unannotated genes for a mtDNA-encoded tmRNA in a few other eukaryotes and, where genome sequence data are also available for these species or close relatives, have been able to identify a co-occurring nucleus-encoded SmpB (Fig. 4; Additional file 4: Table S3 (B)). These observations indicate that a bacterial-type *trans*-translation rescue system acting in conjunction with mitochondrial ribosomes (mitoribosomes) has been lost several times independently across eukaryotes.

#### Aminoacyl-tRNA synthetases

We identified 19 mitochondrial aminoacyl-tRNA synthetases in *Andalucia*, one for each amino acid except glutamine. We also identified the three subunits of Glu-tRNA(Gln) amidotransferase. This pathway is the likely route to Gln-tRNA in *Andalucia* mitochondria, as it is also in human mitochondria [113], although the B subunit, GatB/YqeY, lacks a predicted MTS.

We found three mitochondrion-targeted enzymes that together are involved in formation and turnover of the N-terminal formyl-Met residue that begins protein synthesis in mitochondria: a Met-tRNA formyltransferase, a peptide deformylase, and a methionyl aminopeptidase.

#### Small ribosomal subunit (SSU)

**Fig. 5:**
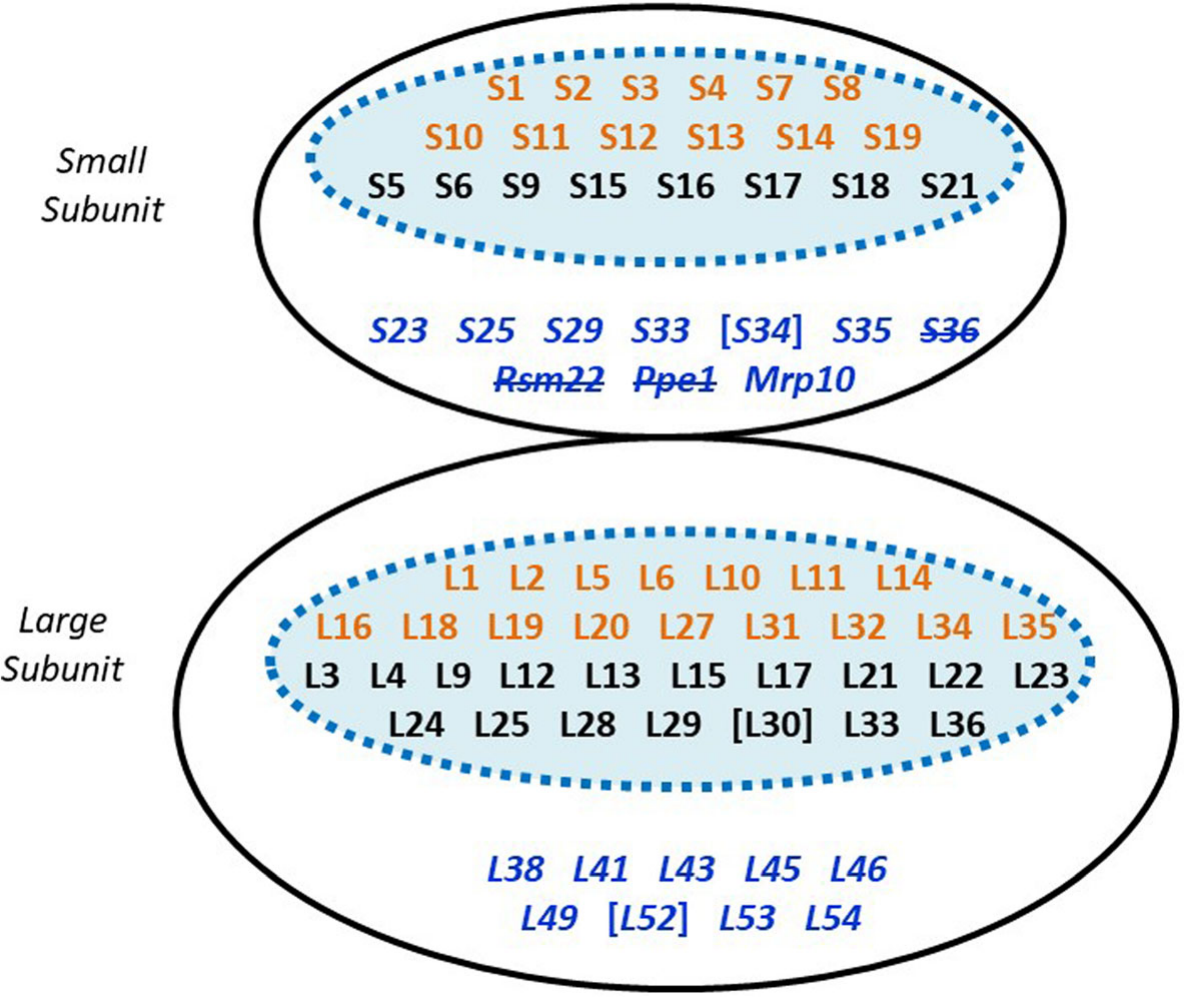
Composition of the *A. godoyi* mitochondrial ribosome. The figure lists the mtRPs inferred to have been present in the mitoribosome of LECA [114]. The blue-shaded ovals encompass those mtRPs directly inherited from the alphaproteobacterial ancestor of mitochondria (those shown in orange are mtDNA-encoded in *A. godoyi*, black are nuclear DNA-encoded). Proteins shown in blue italics are eukaryote-specific additions. Proteins set in square brackets are present in both *A. castellanii* and *N. gruberi* but were not retrieved from *A. godoyi* genomic or transcriptomic data, despite extensive searches. Three components (strikethrough) have subsequently been shown to be absent in the cryo-EM structure of the yeast mitoribosome [115], with S36 having another, non-ribosomal function [116]. We identified *Andalucia* homologs of these three proteins, with S26 and Ppe1 predicted to be mitochondrion-targeted

*Andalucia* encodes all 20 SSU mitochondrial ribosomal proteins (mtRPs) of alphaproteobacterial origin that Desmond et al. [114] inferred were present in LECA. Twelve of these proteins (S1, S2, S3, S4, S7, S8, S10, S11, S12, S13, S14, and S19) are still encoded in *Andalucia* mtDNA (Fig. 5). A homolog of bacterial S20 has not been identified in any eukaryote and is therefore presumed to have been lost at a very early stage in eukaryote evolution.

Desmond et al. [114] inferred the presence of 10 additional, eukaryote-specific SSU mtRPs in LECA. With one exception (S34), we identified orthologs of these proteins in *Andalucia*. A candidate S34 sequence has so far not been retrieved from either the *Andalucia* genome or transcriptome data, despite exhaustive BLAST and HMM-based searches with a variety of queries. A candidate S34 ortholog was identified in a proteomic analysis of *A. castellanii* mitochondria (supported by MS data [10]) and could be retrieved by BLAST in the case of *N. gruberi*. Conversely, we identified candidate *Andalucia* mS26 and mS38 orthologs, which were not among the list of proteins inferred by Desmond et al. [114] to have been present in the mitoribosome of LECA. Desai et al. [115] recently demonstrated that yeast Cox24 is a component of the yeast mitoribosome and corresponds to mS38. These findings indicate that mS38 was most likely present in the LECA mitoribosome.

Notably, in the complete yeast mitoribosome structure determined by Desai et al. [115], no S36 component could be identified; rather, the corresponding protein has been renamed Kgd4 and shown to recruit the E3 subunit to the E1-E2 core of the mitochondrial α-ketoglutarate dehydrogenase [116]. Desai et al. [115] also failed to identify Rsm22 and Ppe1 (Fig. 5) in their structure, suggesting that these components may be only transiently associated with the yeast mitoribosome and not bona fide mtRPs.

#### Large ribosomal subunit (LSU)

Desmond et al. [114] inferred 33 LSU mtRPs of alphaproteobacterial origin in LECA, 16 of which are encoded in *Andalucia* mtDNA (L1, L2, L5, L6, L10, L11, L14. L16, L18, L19, L20, L27, L31, L32, L34, L35). With the exception of L30, genes for orthologs of all remaining proteins have been identified in the *Andalucia* nuclear genome (Fig. 5). Queries with a variety of L30 sequences failed to retrieve a homolog from the *Andalucia* data, although we identified one in both *A. castellanii* and *N. gruberi*. Jakobid mitochondrial genomes encode a bacterial-type 5S rRNA [30], and it is notable that the inferred *Andalucia* mitoribosome has orthologs of all three mtRPs (L5, L18, L25) essential for incorporation of 5S rRNA into the bacterial large ribosomal subunit [117].

Of 9 additional eukaryote-specific LSU mtRPs inferred by Desmond et al. [114] to have been in LECA, we retrieved candidate sequences for all but L52 (present in both *A. castellanii* and *N. gruberi*). We also found orthologs of mL40 and mL59 (= Mrpl25), considered by Desmond et al. [114] to be restricted to opisthokonts and fungi, respectively.

Three ribosomal proteins that are nucleus-encoded in *Andalucia* are mtDNA-encoded in some other eukaryotes: Rps16 is mtDNA-encoded in *A. castellanii* [10] and other amoebozoans, malawimonads, and apusomonads [85]; Rpl23 is mtDNA-encoded in *Diphylleia* [118]; and Rpl36 is mtDNA-encoded in malawimonads [118]. To date, these are the only three mtDNA-encoded ribosomal proteins whose genes have not been identified in any jakobid mtDNA [30].

#### RNA modification enzymes

Including the SSU rRNA DMT discussed earlier, we have identified more than 20 enzymes involved in post-transcriptional modification of *Andalucia* mitochondrial rRNA and tRNA. In addition to a variety of methyltransferases and pseudouridine synthases, these enzymes include several activities involved in formation of hypermodified residues located in the anticodon loop in tRNA (e.g., mnm^5^U, m^6^t^6^A, i^6^A). We identified orthologs of seven human genes (GTPBP, MTO1, NSUN3, TRIT1, TRMT5, TRMU, TRNT) encoding mitochondrial rRNA or tRNA modification enzymes that are linked to mitochondrial energy generation disorders [119].

#### Other ribosome-associated proteins

Assembly of bacterial ribosomes is facilitated by a variety of GTPases [120]. We identified mitochondrion-targeted *Andalucia* homologs of seven bacterial ribosome assembly GTPases: Era, TypA/BipA, Obg/CgtA, Der/EngA, RbgA, YqeH, and YlgF. We also retrieved YchF (an ATPase) and RbFA, two other ribosome-binding proteins that are implicated in ribosome assembly and/or regulation. Finally, we found an endonuclease L-PSP ortholog, a translation inhibitor protein that is thought to act by degrading mRNA.

### Membrane structure and function (E)

We identified several proteins known to be integral mitochondrial membrane proteins in other organisms, including three Band-7 proteins [121]—prohibitins 1 and 2 (Phb1, Phb2) and a stomatin-like protein—as well as transmembrane protein 14C-like and 53-like orthologs and two Mpv17/PMP22 superfamily members. Sym1, the yeast ortholog of the latter protein, is a stress-induced bioenergetic and morphogenetic protein localized in the MIM [122]. Mpv17 was recently shown to prevent uracil accumulation in DNA, possibly by acting as a transporter that transfers dTMP from the cytosol to mitochondria to sustain mtDNA synthesis during replication [123].

Three human mitochondrial NEET family proteins, HsCISD1 (mitoNEET), HsCISD2 (NAF-1), and HsCISD3 (MiNT/Miner2), contain a signature zf-CDGSH consensus sequence as part of a [2Fe-2S]-binding domain. This protein family is highly conserved evolutionarily, throughout bacteria as well as eukaryotes, and is thought to have a role in the regulation of various iron, Fe-S, and reactive oxygen/redox reactions in cells [124]. We retrieved three *Andalucia* CDGSH Fe-S domain-containing proteins, named here AgCISD_1, AgCISD_2, and AgCISD_3. In sequence similarity and domain structure, AgCISD_1 and AgCISD_2 are most closely related to HsCISD3: all three proteins exhibit tandem zf-CDGSH domains and lack the N-terminal MitoNEET_N (transmembrane) domain present in HsCISD1 and HsCISD2. However, HsCISD3 also contains a MTS (directing it to the mitochondrial matrix), whereas the *Andalucia* homologs do not. On the other hand, AgCISD_3, which has a single degenerate CDGSH Fe-S domain, is strongly predicted to be targeted to mitochondria. Whether AgCISD_1 and AgCISD_2 are actually mitochondrial proteins in *Andalucia* is questionable, and the evolutionary relationships between the human CDGSH proteins and their *Andalucia* counterparts remain to be determined.

Two mitochondrial outer membrane (MOM) proteins are Msp1 and monoamine oxidase (MAO), orthologs of which we identified in this study. Msp1 is an AAA+ superfamily ATPase that clears mistargeted proteins by facilitating their transfer from mitochondria to the endoplasmic reticulum [125]. MAOs are characterized by a globular water-soluble main body that is anchored to the mitochondrial membrane via a C-terminal α-helix [126]. Mammalian MAO comes in two forms, A and B, and their activity has been implicated in oxidative stress [127]. *Andalucia* also encodes two distinct MAO forms, but both are specifically related to the MAO A type.

Finally, we identified a predicted mitochondrion-targeted ortholog of MICS1, a mitochondrial protein involved in maintenance of mitochondrial morphology and apoptotic release of cytochrome *c* [128].

#### ERMES (ER-mitochondria encounter structure)

**Fig. 6:**
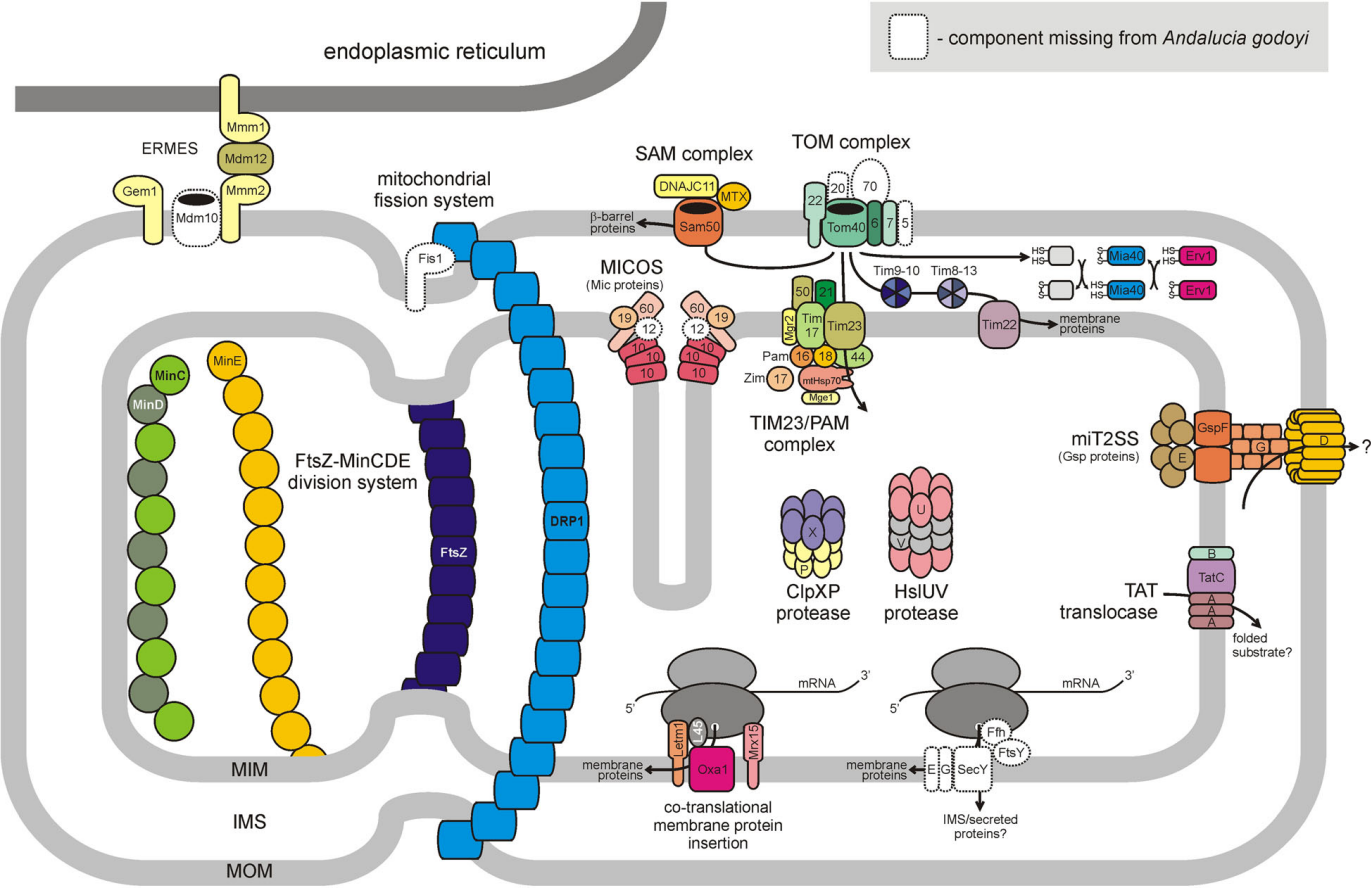
Schematic view of the *A. godoyi* mitochondrion showing selected components related to organelle biogenesis and protein trafficking/turnover. Individual proteins are drawn as shapes that do not necessarily represent accurately the actual structure of the protein, and the spatial arrangement of the proteins does not necessarily reflect the actual interactions of different subunits within protein complexes. For simplicity, interactions between complexes (e.g., MICOS-SAM, SAM-TOM) are not shown. Arrows indicate pathways of protein trafficking. The proteins in white and with a dashed outline were not identified in *Andalucia* (only components whose absence is of particular significance are included). Details of the figure are drawn from various sources. MIM, mitochondrial inner membrane; IMS, intermembrane space; MOM, mitochondrial outer membrane

ERMES, a protein complex that physically links the ER (endoplasmic reticulum) membrane to the MOM, is an ancient and widespread mitochondrial feature [129]. Originally identified in fungi, yeast ERMES comprises four mitochondrial distribution and morphology (Mdm) proteins: a MOM β-barrel protein Mdm10 and three SMPs (synaptagamin-like, mitochondrial, lipid-binding protein domain-containing proteins): ER-localized Mmm1; Mmm2 (Mdm34); and Mdm12, which forms a cytosolic bridge. In *Andalucia*, we identified orthologs of Mmm1, Mmm2/Mdm34, and Mdm12 but not Mdm10 (Fig. 6). The latter protein has been reported to be absent from a number of discoban lineages except *Naegleria* [129]. We also identified an ortholog of the MOM-anchored GTPase Gem1 (Miro) that may regulate ERMES activity, although that inference has been challenged [130]. Finally, we did not identify homologs of Mdm31 and Mdm32, inner membrane proteins required for maintenance of mitochondrial shape and stability of mitochondrial DNA nucleoids in yeast [131].

#### MICOS (mitochondrial contact site and cristae-organizing system)

Recent work proposes that MICOS, which controls the formation and maintenance of mitochondrial cristae, evolved directly from intracytoplasmic membranes specific to *Alphaproteobacteria* [132–135]. The MICOS complex of yeast comprises six proteins: Mic10, Mic12, Mic19, Mic26, Mic28, and Mic60. Mic60 is the most ancient component, apparently originating within *Alphaproteobacteria*, whereas Mic10 is the most widespread of the MICOS subunits among eukaryotes [132, 133]. Mic26 and Mic28 are found only within opisthokonts, whereas Mic10, Mic12, and Mic19 (Mic25) are considered to trace back to LECA [134] *Andalucia* was previously reported to encode Mic60, Mic10, and Mic19 [133] (Fig. 6). We confirm the identification of these orthologs and add that no *Andalucia* Mic12 candidate could be found even by recapitulating the highly sensitive search procedure that enabled identification of putative plant Mic12 proteins [134].

Muñoz-Gómez et al. [132] examined the phylogenetic distribution of proteins reported to interact with MICOS and identified three (DNAJC11, Sam50, Mia40) in *Andalucia*. We additionally found a Cox17 ortholog, a CIV assembly protein (see Table 3) missed by Muñoz-Gómez et al. [132]. The ATPase assembly factor Nca2 (see Table 3) may also be functionally linked to MICOS in *Andalucia*, analogously to the role of its plant ortholog [136].

#### Mitochondrial fusion/fission

An ancestral mitochondrial division system, inherited from the alphaproteobacterial progenitor of mitochondria, is widespread among eukaryotes, including *A. godoyi* [137] (Fig. 6). This system comprises the cell division protein FtsZ (AgFtsZ1 and AgFtsZ2 in *Andalucia*) and the septum-site determining factors MinC, MinD, and MinE. We identified, in addition, two dynamin-family proteins, AgDFP1 and AgDFP2 (Dmn1/Drp1 homologs). AgDFP1 appears to be an authentic Dmn1/Drp1 ortholog that may be implicated in mitochondrial fission; however, *Andalucia* appears to lack homologs of all the proteins known to recruit DRP1 to the MOM in metazoans, yeasts, or plants [138], including the broadly conserved Fis1. On the other hand, *Andalucia* does have an ortholog of the MIM protein MTFP1 (MTP18), which contributes to mitochondrial fission in mammalian cells by a poorly understood mechanism involving DRP1 [139, 140].

AgDFP2 is a novel dynamin-related protein that may be specific to *Andalucia* and may be localized to the mitochondrion owing to a weakly predicted MTS. It is not directly related to other intramitochondrial dynamin-related proteins, e.g., OPA1, Mgm1, and MidX, of some other eukaryotes [133] and its function remains unclear.

Somewhat curiously, we did not identify *Andalucia* homologs of proteins, notably mitofusin, known to be involved in mitochondrial fusion in a variety of other eukaryotes. The fact that *A. godoyi* appears to have a single mitochondrion [26] may account for this absence. We did find an ortholog of mitochondrial transmembrane protein 135 (TMEM135), which appears to be involved in regulating the balance between mitochondrial fusion and fission [141]. The *Andalucia* ortholog is bipartite, with TMEM135_A and TMEM135_B corresponding, respectively, to the N-terminal half (TMEM135_C_rich domain; PF15982) and C-terminal half (Tim17/Tim22/Tim23/Pmp24 family member; PF02466) of human TMEM135. Curiously, the order of the two domain variants differs between metazoan and plant TMEM135 proteins, leading Žárský and Doležal [142] to conclude that the two domains have been swapped during evolution. Notably, genes for the A and B portions of *Andalucia* TMEM135 are not physically linked in the genome.

*Andalucia* also encodes an ortholog of human MSTO1 (misato), a cytoplasmic protein implicated in mitochondrial distribution and morphology [143, 144]. However, this protein has essential roles beyond mitochondria, notably in the assembly of spindle microtubules [145].

### Protein and metabolite trafficking (F)

#### Protein trafficking

As discussed by Wiedemann and Pfanner [146], five major mitochondrial protein import pathways have been described so far in model systems such as yeast and human, each one characterized by a different type of targeting signal. Figure 6 presents a schematic summary of identified and absent *Andalucia* components of the various pathways and complexes discussed below.

##### Presequence (classical) pathway (Table 4) *Translocase of the outer mitochondrial membrane (TOM)*

In human and yeast mitochondria, TOM consists of three receptor proteins (Tom20, Tom22, and Tom70), the channel-forming protein Tom40, and three small Tom proteins (Tom5, Tom6, and Tom7). Tom20 and Tom22 preferentially recognize preproteins that contain an N-terminal MTS, whereas Tom70 plays a major role in the import of non-cleavable hydrophobic proteins such as MC proteins involved in metabolite transport. Movement of receptor-bound proteins through the Tom40 channel results in translocation through the OM. The primordial TOM complex is thought to have consisted of Tom40, Tom22, and Tom7 [147, 148].

**Table 4:**
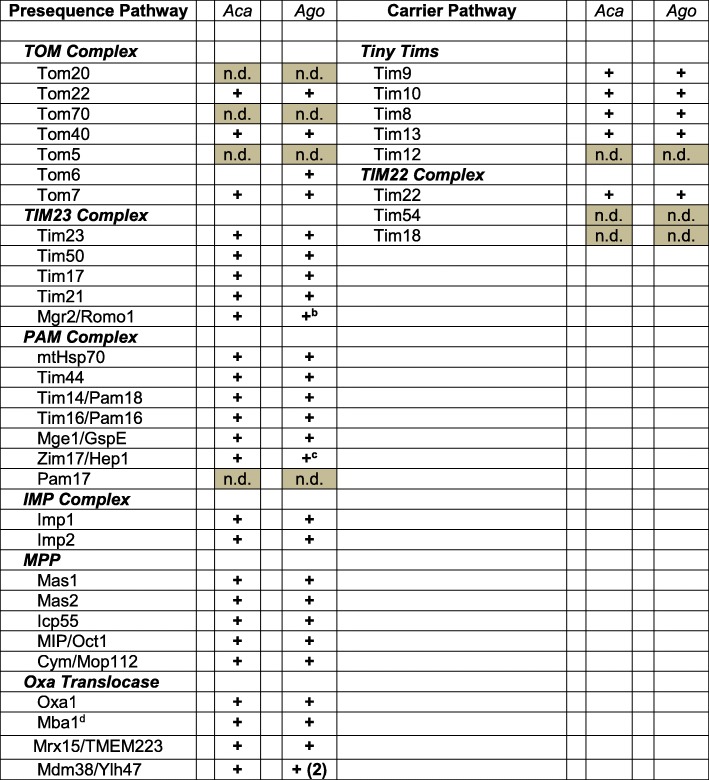
Composition of presequence and carrier pathways for mitochondrial protein import

The table lists components typically found in the various complexes in opisthokont (animal and fungal) mitochondria [146]. A plus sign (+) indicates the presence of a homologous protein in *A. godoyi* (*Ago*) and/or *A. castellanii* (*Aca*); a gray rectangle with n.d. (not detected) denotes absence of a component. Related proteins retrieved for *A. godoyi* include a Tim17/Tim22/Tim23 family protein and an additional mitochondrial intermediate peptidase (see text)

^b^XP_004333275.1

^c^XP_004335035.1

^d^mtRP L45 homolog

We identified *Andalucia* orthologs of Tom40, Tom22, Tom6, and Tom7, but did not retrieve Tom20, Tom5, or Tom70 (Table 4). A recent study by Mani et al. [149] failed to find these three proteins in other discobans (kinetoplastids and *N. gruberi*), and they were also not identified in an MS analysis of *A. castellanii* mitochondria [10] (Table 4). These observations suggest that some of the “missing” Tom proteins may be later clade-specific additions to a simpler ancestral TOM complex [149]. On the other hand, our finding of the *Andalucia* Tom6 ortholog—the first outside opisthokonts and plants—reinforces the notion that it was part of the TOM complex in LECA.

##### *Translocase of the inner mitochondrial membrane TIM23*

*Presequence translocase:* The TIM23 complex comprises four core subunits: Tim50, the MIM presequence receptor; Tim23, which forms the MIM import channel; Tim17, closely associated with Tim23 and involved in regulating the Tim23 channel and in preprotein sorting at the MIM; and a small protein, Mgr2 [150], which serves as a lateral gatekeeper for preproteins that are sorted into the lipid phase of the MIM [151] and has recently been implicated in regulation of the gating behavior of the TIM23 channel [152]. The human Mgr2 ortholog is Romo1 (reactive oxygen species modulator 1), a protein involved in reactive oxygen species (ROS) production [153]. We identified orthologs of all four of these TIM23 proteins in *Andalucia* (Table 4). We also retrieved what appears to be a highly divergent homolog of regulatory subunit Tim21, which links TIM23 to the respiratory chain III-IV supercomplex, promoting insertion of proteins into the MIM.

*Presequence translocase-assisted motor (PAM complex)*: The ATP-driven chaperone mitochondrial heat-shock protein 70 (mtHsp70) forms the core of PAM, which is crucial for the import of matrix proteins. Tim44 couples mtHsp70 to the TIM23 channel; the membrane-bound co-chaperones Tim14 (Pam18) and Tim16 (Pam16) regulate mtHsp70 ATPase activity; nucleotide exchange factor Mge1 (co-chaperone GspE) promotes ADP release from mtHsp70; and chaperone Zim17 (Hep1) prevents aggregation of mtHsp70. We identified orthologs of all six of these proteins in *Andalucia* (Table 4).

We were not able to retrieve Pam17, an additional regulatory component of the fungal TIM23 complex; this component was also not found during proteomic analysis of *Acanthamoeba* mitochondria [10].

*Inner membrane peptidase (IMP) complex*: IMP is responsible for removing the hydrophobic sorting signal from MIM proteins, resulting in their release into the intermembrane space (IMS). Of the three subunits that make up the IMP complex in yeast [154], we retrieved *Andalucia* orthologs of Imp1 and Imp2 but not Som1.

*Matrix presequence processing*: In the matrix, the dimeric matrix processing peptidase (MPP)—an integral part of respiratory CIII—removes N-terminal targeting presequences, which are degraded by the metallopeptidase Cym1 (presequence protease, PreP). Icp55 (intermediate cleaving peptidase 55) and mitochondrial intermediate peptidase MIP1 (Oct1) remove N-terminal destabilizing residues from imported, MPP-cleaved proteins. We identified all five of these proteins, as well as an additional MIP-like protein.

*Oxidase assembly (OXA) translocase*: The OXA translocase functions in the insertion of proteins synthesized by mitoribosomes into the MIM. We identified Oxa1, the main component of this translocase. Three additional components, Mba1, Mdm38, and Ylh47, serve as ribosome receptors in yeast, assisting in co-translational protein insertion, with paralogous Mdm38 and Ylh47 proteins having overlapping functions [155]. We identified a LETM1 and EF-hand domain-containing protein as a putative Mdm38/Yih47 ortholog. Mba1 is considered to be the fungal version of mammalian mtRP L45 [114, 156], an *Andalucia* ortholog of which we identified here (Fig. 5). The fact that mitochondrial mtRP L45 is a Tim44 superfamily protein is consistent with a proposed role for this protein as a MIM receptor for the mitoribosome.

In *S. cerevisiae*, Mba1 and another ribosome receptor, Mrx15, cooperate in organizing mitochondrial cotranslational insertion and protein biogenesis [157]. Yeast Mrx15 is homologous to human transmembrane protein 223 (TMEM223) (unpublished observations). *Andalucia* encodes an Mrx15/TMEM223 ortholog, weakly predicted to be targeted to mitochondria.

##### Carrier pathway

After synthesis on cytosolic ribosomes, MC proteins (described in more detail below), which lack a cleavable presequence, are bound to cytosolic Hsp70 and Hsp90 class chaperones and delivered to the MOM Tom70 receptor, which possesses binding sites for both carrier protein and chaperone. From there, the carrier protein is transferred to the central Tom22 receptor before being passed through the Tom40 channel of the TOM translocase complex. Because *Andalucia* lacks a recognizable Tom70 component, it is not clear how carrier proteins would be transported to and imported through the Tom40 channel. Evolutionarily unrelated alternative receptors to Tom70 have been identified in plants [158] and trypanosomes [159], so it is possible that the corresponding component in *Andalucia* remains to be identified.

##### *Tiny Tims*

Once through the Tom40 channel, carrier proteins are handed directly to soluble heterohexameric chaperone complexes, Tim9-Tim10 or Tim8-Tim13. We identified orthologs of all four of these small chaperones (“Tiny Tims”) in *Andalucia*. All of them contain a twin Cx_3_C motif, one of the two motifs characteristic of substrates of the MIA intermembrane import pathway [160] (see below).

##### *Translocase of the inner mitochondrial membrane TIM22*

In fungi, the TIM22 complex comprises Tim22, the channel-forming protein; Tim54, a receptor-like protein; and Tim18, which like Tim22 is imbedded in the MIM. Of these, we detected only Tim22 in *Andalucia*, consistent with recent work [161] showing that Tim54 and Tim18 are fungal-specific components. As expected, *Andalucia* also lacks a homolog of the mammalian-specific Tim29 subunit [162].

Where present, Tim54 recruits a Tim9-Tim10-Tim12 complex into the TIM22 complex; however, as noted above, formation of the former complex is evidently precluded in *Andalucia* by the apparent absence of a Tim12 homolog, which might explain the concurrent absence of Tim54. This raises the question of how carrier proteins chaperoned by Tim9-Tim10 or Tim8-Tim13 are delivered to and enter the Tim22 channel in *Andalucia* mitochondria.

Tim18 is responsible for incorporating Sdh3, which is a subunit of respiratory Complex II, into a Tim18-Sdh3 module that forms part of TIM22, the Sdh3 protein having a dual role in both mitochondrial bioenergetics and protein transport [163]. The apparent absence of Tim18 in *Andalucia* would preclude formation of a Tim18-Sdh3 module. Notably, the three TIM22 proteins that we were not able to retrieve from our *Andalucia* data (Tim12, Tim18, and Tim54) were also not identified in an MS/MS-based proteomic analysis of *A. castellanii* mitochondria (Table 4), suggesting either that the corresponding proteins remain to be identified or that TIM22 might have a simpler structure in jakobids and amoebozoans than in animals and fungi.

##### Sorting and assembly (SAM) machinery

The SAM complex, which mediates insertion of β-barrel proteins into the MOM, comprises a central membrane-integrated protein, Sam50, and associated proteins exposed to the cytosol: Sam35 and Sam37/Tom37 in yeast and their orthologs metaxins in metazoans. Sam37/Tom37 promotes β-barrel biogenesis by interacting with the cytosolic receptor domain of Tom22, thereby forming a transient TOM–SAM supercomplex [164].

We identified an *Andalucia* Sam50 ortholog and a single protein (AgMTX_1) clearly related to Sam35/Sam37/metaxins, in agreement with the general presence of a single metaxin gene in eukaryotes outside opisthokonts [134]. Interestingly, *Andalucia* possesses another, more distant metaxin relative (AgMTX_2) that displays highest similarity to bacterial, especially gammaproteobacterial, glutathione-S-transferases, suggesting the possibility of LGT in this case. The protein DNAJC11, which is also present in *Andalucia* (see above), associates with Sam50 and metaxins as part of the mitochondrial IMS bridging complex [134].

The morphology proteins Mdm12 and Mmm1, discussed earlier in the context of the ERMES complex and present in *Andalucia*, also function in the major β-barrel assembly pathway of mitochondria [165]. Mdm10, which is also a component of the ERMES complex, associates with the SAM core to form a SAM-Mdm10 complex that in turn interacts with the precursor of Tom22, providing an assembly platform for the TOM complex. Because Mdm10 appears to be absent in *Andalucia* mitochondria, such a TOM-SAM interaction would presumably not be possible. It has been suggested that Mdm10 and the TOM subunit Tom7 are part of a regulatory network that controls assembly of the TOM complex and links it to ERMES [146]. In this context, it is notable that Tom7 but not Mdm10 appears to be present in *Andalucia*, which might suggest that Tom7 does not have the dual role in *Andalucia* that it has in yeast [166].

##### Mitochondrial intermembrane space import and assembly (MIA) machinery: disulphide relay

The IMS houses an oxidative protein-folding system that catalyzes the formation of disulphide bonds in imported proteins, which promotes their conformational stabilization and assembly. This machinery operates as a disulphide relay in which the oxidoreductase Mia40 cooperates with the sulfhydryl reductase Erv1. This system evidently operates in *Andalucia* as we have identified Mia40 and two forms of Erv1, although we have not found a counterpart of Hot13, a yeast zinc-binding protein that promotes the oxidation of Mia40 by keeping it in a zinc-free state [167].

In addition to the twin Cx_3_C MIA import motif mentioned above, a twin Cx_9_C motif is also characteristic of substrates imported via the MIA pathway [160]. In addition to Mia40 itself, *Andalucia* proteins containing this motif include subunits of ETC complexes CI (NDUFS5, NDUFA8, NDUFB7) and III (Qcr6) as well as a number of CIV assembly proteins (Cmc1, Cmc2, Coa4, Cox5/Pet151, Coa6, Cox17, Cox19, and Cox21).

##### Preprotein mitochondrial import machinery of the outer membrane

The preprotein import machinery acts as an insertase for certain α-helical MOM proteins, specifically signal-anchored proteins, which have an α-helical transmembrane segment at the N-terminus, and polytypic ones, which contain multiple transmembrane segments [146]. This particular import complex comprises two subunits, Mim1 and Mim2, whose phylogenetic distribution is almost exclusively fungal. Not surprisingly, we failed to find homologs of these two proteins in *Andalucia*.

##### Additional protein translocation systems

*Andalucia* contains genes encoding two additional protein-conducting pathways typically found in bacteria. The Twin-Arginine Translocation (TAT) pathway is localized to the bacterial cytoplasmic membrane and allows the transmembrane passage of fully folded proteins. In proteobacteria, the minimal active TAT system consists of three proteins, TatA, TatB, and TatC [168]. Orthologs of TatA and TatC, encoded by mtDNA, have a scattered distribution among eukaryotes, but whether these proteins constitute an active system in the organelle is not clear [169]. In plants, whose mitochondrial genomes encode only TatC, a TatB but apparently not TatA ortholog is encoded in the nuclear genome, and a functional TatB-TatC complex has been proposed in this case [170]. In *Andalucia*, we identified a nucleus-encoded TatB in addition to mitochondrion-encoded TatA and TatC (Fig. 6), which is also the case in other jakobids (unpublished results). This represents the only eukaryotic system described so far in which all three minimal TAT subunits have been identified, strongly suggesting that a functional TAT pathway exists in *Andalucia* mitochondria.

The second bacterial-type protein translocation pathway found in *Andalucia* is characterized in detail in a separate study [171]. Briefly, jakobids (including *A. godoyi*) and representatives of several other eukaryotic lineages (heteroloboseans, malawimonads, hemimastigophorans) possess homologs of the core components of the Type II Secretion System (T2SS) seemingly localized to the mitochondrial envelope. This system, denoted miT2SS, is presumed to mediate secretion of an unknown substrate from the mitochondrion (Fig. 6). Several additional proteins, some of them possibly mitochondrial, exhibit precisely the same phylogenetic distribution as miT2SS, suggesting the existence of a more complex miT2SS-linked functional system.

In bacteria, most proteins cross the cytoplasmic membrane via the Sec translocase, which comprises a protein-conducting channel SecYEG and an ATP-dependent motor protein SecA [172]. A SecY homolog is encoded in the mtDNA of all sequenced jakobid mtDNAs with the exception of *A. godoyi* [30]. HMMER searches of *A. godoyi* genome and transcriptome data failed to retrieve a candidate protein, leading us to conclude that SecY has not been transferred to the nuclear genome, but has indeed been lost. Furthermore, neither SecE nor SecG homologs could be identified. It will be interesting to analyze nuclear genomes of other jakobids to determine whether they might specify a complete SecYEG complex. It is also noteworthy that *Andalucia* lacks homologs of bacterial Ffh (the protein component of signal recognition particle, SRP) and FtsY (SRP receptor), which in bacteria cooperate with the SecYEG complex to mediate co-translational membrane protein insertion [173], and which have recently been identified as putative mitochondrial proteins in a few eukaryotic lineages (unpublished observations).

#### Metabolite trafficking

##### Mitochondrial carrier (MC) system

The MC system, whose integration into the MIM was described above, comprises a large number of related proteins that function in the transport of a variety of small metabolites from the cytosol into the mitochondrion. MC proteins (also referred to as the SLC25 family) contain characteristic *trans* membrane-spanning alpha helices (MC_carr domains [174]). More than 50 members of this family have been reported in mammalian mitochondria [175]. In *Andalucia*, we identified 36 MC homologs, comprising 17 different KEGG orthology (KO) groups predicted to transport a variety of metabolites (Table 5). More than 80% of these MC proteins exhibit a tripartite Mito_carr domain structure. We found multiple carriers having predicted substrate specificities for phosphate (4), adenine nucleotide (2), aspartate/glutamate (5), carnitine/acylcarnitine (7), iron (2), and folate (3). Overall, in terms of number and predicted metabolite specificity, the *Andalucia* MC family parallels that of animals, fungi, and land plants.

**Table 5:** Mitochondrial carrier (MC) proteins identified in *A. godoyi*

AgMC^a^	KO entry	KEGG member	Predicted substrate specificity	No. of Mito_carr domains^b^
1	K15102	3	Phosphate	3
2	“	3	Phosphate	2
3	K05863	4/5/6/31	Adenine nucleotide	1
4	“	4/5/6/31	Adenine nucleotide	1
5	K13577	10	Dicarboxylate	3
6	“	10	Dicarboxylate	3
7	K15104	11	Oxoglutarate	3
8	K15105	12/13	Aspartate/glutamate	3
9	“	12/13	Aspartate/glutamate	3
10	“	12/13	Aspartate/glutamate	3
11	“	12/13	Aspartate/glutamate	3
12	“	12/13	Aspartate/glutamate	3
13	K15106	14/30		3
14	K15084	16		3
15	K15109	20/29	Carnitine/acylcarnitine	3
16	“	20/29	Carnitine/acylcarnitine	3
17	“	20/29	Carnitine/acylcarnitine	3
18	“	20/29	Carnitine/acylcarnitine	3
19	“	20/29	Carnitine/acylcarnitine	3
20	“	20/29	Carnitine/acylcarnitine	2
21	“	20/29	Carnitine/acylcarnitine	3
22	K15110	21	2-Oxodicarboxylate	3
23	K14684	23/24/25/41	Phosphate	3
24	“	23/24/25/41	Phosphate	3
25	K15111	26	S-Adenosylmethionine	3
26	K15113	28/37	Iron	3
27	“	28/37	Iron	3
28	K15115	32	Folate	3
29	“	32	Folate	3
30	“	32	Folate	2
31	K15116	33/36		3
32	“	33/36		1
33	K15118	38		3
34	K15119	39/40		3
35	K15120	43		3
36				3

^a^See Additional file 1: Table S1 (F) PROTEIN & METABOLITE TRAFFICKING, for sequences of individual MC proteins, which were annotated via the KEGG Automatic Annotation Service (KAAS) [176] using default parameters (BLAST; BBH: bidirectional best hit). All except AgMC36 (which was not annotated by KAAS) are predicted to be solute carrier family 25 (SLC25) members

^b^Determined by BLASTp at NCBI

Several of the *Andalucia* MC proteins appear to be specifically related to functionally well-characterized MC proteins in other eukaryotic systems. For example, AgMC1 is an ortholog of mammalian phosphate carrier SLC25A3, a mitochondrial copper transporter required for cytochrome *c* oxidase biogenesis [177]. AgMC5 and AgM7 are orthologs, respectively, of *Arabidopsis thaliana* UCP1 and UCP2, originally characterized as uncoupling proteins but more recently shown to be transporters of asparatate, glutamate, and dicarboxylates [178]. Both AgMC11 and AgMC23 have N-terminal calcium-binding domains in addition to C-terminal Mito_carr domains; in contrast to almost all other MC proteins, these variants have an N-terminal MTS, with AgMC11 in particular being strongly predicted to be targeted to mitochondria. AgMC23 appears to be the ortholog of the mammalian ATP-Mg/P_i_ carrier responsible for the calcium-dependent regulation of adenosine nucleotide concentrations in the mitochondrial matrix [179].

##### Non-MC transporters

In addition to MC proteins, we identified 14 candidate *Andalucia* proteins having a putative role in metabolite transport into mitochondria. These include the MOM protein porin (VDAC); three proteins involved in calcium uptake, including orthologs of the human calcium uniporter protein MCU and calcium uptake protein 1 (MICU1) [180]; an ortholog of human SFXN1 (sideroflexin), a serine transporter required for 1-C metabolism [181]; two putative pyruvate transporters; a CorA-like Mg^2+^ transporter; and four ABC transporters.

### General metabolism (G)

*Andalucia* mitochondria carry out a complex metabolism, exhibiting complete pathways for expected highly conserved and ubiquitously distributed functions such as Fe-S cluster biosynthesis (including Bol1, a specific mitochondrial Fe-S cluster assembly factor that facilitates [4Fe-4S] cluster insertion into a subset of mitochondrial proteins [182]), branched chain amino acid degradation, ubiquinone biosynthesis, and glycine cleavage (the latter system including all four catalytic proteins but with the apparent absence of the regulatory R protein, which is also absent in *N. gruberi*). We also identified four enzymes in the porphyrin biosynthesis pathway although two of them lack an N-terminal MTS, as do their MS-authenticated orthologs in *Acanthamoeba*. A complete pathway for fatty acid oxidation is also present, including alpha and beta subunits of the trifunctional enzyme (hydroxyacyl-CoA dehydrogenase/3-ketoacyl-CoA thiolase/enoyl-CoA hydratase), orthologs of the human HADHA and HADHB proteins, respectively.

We retrieved orthologs of the four central enzymes in the cardiolipin biosynthesis pathway: CDP-DAG synthase, PGP synthase, a dual specificity protein phosphatase, and cardiolipin synthase. The CDP-DAG synthase used in this pathway has been identified in yeast and mammals as the mitochondrial matrix protein Mmp37/Tam41 [183, 184]. We also identified mitochondrial distribution and morphology protein 35 (Mdm35), which contains two characteristic Cx_9_C motifs. Mdm35 mediates the import into the IMS of Ups1 and Ups2, two small atypical IMS proteins that lack conventional IMS-targeting signals [185]. In turn, an Mdm35:Ups1 complex functions to transport phosphatidic acid (PA), used in cardiolipin biosynthesis, from the MOM to the MIM [186]. Finally, we recovered orthologs of two enzymes that function in cardiolipin remodeling: tafazzin (phospholipid lysophospholipid transacylase) and monolysocardiolipin acyltransferase, MLCL AT-1 [187].

In addition to the 57 proteins comprising the specific pathways mentioned above, we identified 102 potential mitochondrial metabolic proteins on the basis of predicted N-terminal targeting. These proteins are involved in a wide array of other pathways, including metabolism of fatty acids, cholesterol, amino acids, nucleotides, coenzymes, and one-carbon fragments, although for some of them specific biochemical activities cannot be assigned and their physiological functions remain unknown. One such protein, a predicted aminotransferase of the DegT/DnrJ/EryC1/StrS family, is particularly interesting as it is a member of a small group of predicted mitochondrial enzymes of bacterial origin with an extremely patchy distribution, being additionally found only in apusomonads and an amoebozoan (Fig. 4; Additional file 5: Figure S2). This distribution is suggestive of dissemination via eukaryote-to-eukaryote LGT, although it is unclear whether the *Andalucia* lineage was a recipient or a donor.

To shed more light on the 102 mitochondrial candidates we identified in the general metabolism category (EC 1 to EC 6), we screened them against a data set of previously identified *Acanthamoeba* mitochondrial proteins [10]. Orthologs of 45 *Acanthamoeba* proteins were retrieved (Additional file 6: Table S4), 42 of which are predicted by our criteria to be mitochondrion-targeted, 89% strongly (29) or moderately (11) so. Of these 45 *Acanthamoeba* proteins, 37 were previously confirmed as mitochondrial by MS data [10]. We infer that these proteins were in place in the mitochondrion of the last common discoban-amoebozoan ancestor and that they are strong candidates to have been present in the LECA mitochondrion.

A further 16 of the 102 *Andalucia* proteins (16%) have an *Acanthamoeba* ortholog for which there are no supporting mitochondrial MS data (Additional file 6: Table S4); also, most of the proteins in this group (13/16) are predicted not to be targeted to mitochondria. In these cases, the mitochondrion-predicted *Andalucia* orthologs might be false positives; alternatively, at least some might represent cases in which the orthologous protein is mitochondrion-localized in *Andalucia* but cytosol-localized in *Acanthamoeba*. Finally, 41 of the 102 *Andalucia* proteins (40%) did not retrieve an evident *Acanthamoeba* ortholog.

Conversely, we encountered a number of cases in which an MS-verified *Acanthamoeba* mitochondrial protein has an *Andalucia* ortholog that is predicted not to be mitochondrion-localized (data not shown). Broadly based comparative analyses of mitochondrial protein composition by MS and other approaches will be necessary to determine to what extent flexibility in subcellular localization of particular metabolic enzymes and other proteins plays a role in mitochondrial and cellular function and evolution.

### Protein folding, processing, and degradation (H)

#### Protein folding

In addition to mtHsp70 and mtHsp90 (human TRAP1 ortholog [188]), previously discussed in connection with protein import, we identified orthologs of a number of chaperones known to play a role in protein folding in other mitochondrial systems, including Hsp20, Hsp60 (also known as chaperonin 60, cpn60), DnaJ (yeast Mdj1p ortholog [189]), and ClpB. We also identified an Hsp10 (cpn10) ortholog, expected since this protein functions together with Hsp60 to promote protein folding in the mitochondrial matrix [146]. Although the Hsp10 ortholog that we retrieved, the only one encoded by the *Andalucia* genome, is not predicted to be targeted to mitochondria, it almost certainly is imported into the organelle as there is no known example where it localizes to, or functions in, any other cellular compartment.

#### Protein processing and degradation

*Andalucia* encodes a variety of mitochondrion-targeted proteins that are presumably involved in protein processing and/or turnover in the organelle. In yeast, two AAA+ proteases, m-AAA+ (Yta10/Agf3, Yta12/Rca1), and i-AAA+ (Yme1), whose catalytic sites are on opposite surfaces of the MIM (facing the matrix and IMS, respectively), function in the selective degradation of misfolded and excess polypeptides [190]. We identified an *Andalucia* ortholog of Yme1 and two highly similar Yta12-like paralogs, as well as two prohibitins (mentioned earlier), which modulate the activity of these proteases. Misfolded proteins in the mitochondrial matrix are also degraded by AAA+ protease Lon, identified here. Additionally, we retrieved an ortholog of AFG1, another AAA+ protease localized to the matrix side of the MIM. Its mammalian homolog, LACE1, mediates turnover of nucleus-encoded CIV subunits COX4, COX5A, and COX6A and is required for normal activity of CIII and CIV [191].

We identified two mitochondrion-targeted, bacterial-type ATP-dependent proteases, HslUV and ClpXP (Fig. 6). HslUV is composed of two subunits, both identified here: HslU (ClpY), an AAA+ ATPase, and HslV (ClpQ), a peptidase homologous to the β subunit of the eukaryotic 26S proteasome [192]. Neither component of the HslUV complex is encoded by animals, fungi, or plants, but both are present in *Acanthamoeba* [10]. In addition to the catalytic ClpP protease, we identified an ortholog of ClpX, an ATP-dependent specificity component of the ClpXP protease complex. We found orthologs of several additional mitochondrion-targeted proteases/peptidases, including a DegP serine endopeptidase, a peptidase S9 family protein (oligopeptidase), an Oma1 zinc metallopeptidase, and an Xaa-Pro dipeptidase. Finally, we identified a homolog of a bacterial-type signal peptidase SppA (protease IV). Although an SppA homolog is known to function in chloroplasts [193], we are aware of only one other reported mitochondrial localization of SppA (verified by MS data), in *A. castellanii* [10].

### Reactive oxygen species (ROS) metabolism, regulation (I)

#### ROS metabolism

Peroxiredoxin (Prx), a thioredoxin-dependent peroxidase, plays a major role in metabolizing hydrogen peroxide generated in the mitochondrial matrix [194]. *Andalucia* contains a PRX5-type Prx with high similarity to alphaproteobacterial homologs, as well as a cytochrome *c* peroxidase (Ccp1). We found a thiol reductase thioredoxin and Grx4 family (GRXS15 type) glutaredoxin; the latter protein participates in the Fe-S cluster biosynthesis pathway in *Arabidopsis thaliana* [195].

**Fig. 7:**
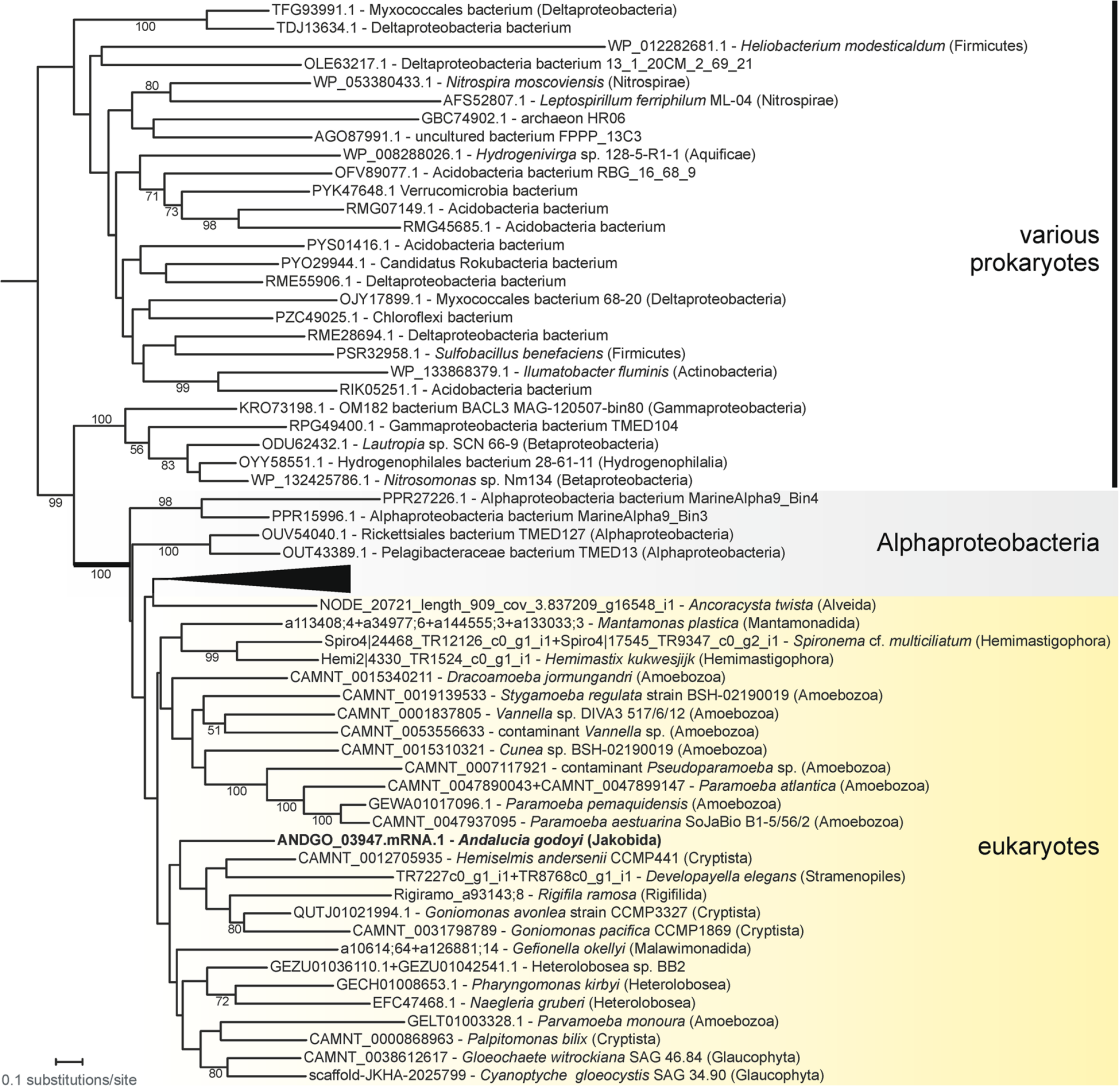
Phylogenetic analysis of RFO proteins. The arbitrarily rooted ML tree was inferred using RAxML (PROTGAMMALG model) based on an alignment of 365 amino acid positions. Branch support values (rapid bootstraps) ≥ 51% are shown. Note the maximal support for the grouping of alphaproteobacterial and eukaryotic sequences. The black triangle represents a large cluster of alphaproteobacterial sequences that was collapsed for simplicity (the full version of the tree is provided as Additional file 7: Figure S3 (A)). Sequences included in the analysis are described in Additional file 4: Table S3 (C)

We identified superoxide dismutase, an expected mitochondrial enzyme and, unexpectedly, a rubrerythrin (RBR) homolog. RBR is a non-heme iron protein implicated in oxidative stress tolerance in anaerobic bacteria and archaea [196]. Among eukaryotes, it has only previously been reported in plastids of glaucophyte algae [197, 198] and in anaerobic taxa including *Entamoeba* and *Mastigamoeba* [199], trichomonads and diplonemids [200], and the amitochondriate oxymonad *Monocercomonoides exilis* [201]. However, the RBR we retrieved here belongs to the three-component “aerobic-type” lineage [202] that comprises, in addition to RBR itself, a predicted Fe-S oxidoreductase (RFO) and a conserved protein of unknown function (DUF3501). Genes encoding these three proteins are physically linked in most bacterial species in which they occur, suggesting the proteins form a functional module. We found *Andalucia* homologs of all three proteins, all with predicted mitochondrial targeting, although their genes are not physically linked. By scanning eukaryotic genome and transcriptome data, we found the aerobic-type RBR in a number of additional lineages, typically together with RFO and DUF3501 homologs. Phylogenetic analyses suggest that the eukaryotic RFO and DUF3501 are of alphaproteobacterial origin (Fig. 7; Additional file 7: Figure S3 (A, B)), which likely holds true also for the aerobic-type RBR (based on the identity of best BLASTp hits; phylogenetic analysis is not feasible because of the limited length of the protein). Furthermore, the mitochondrial localization of all three proteins seems to be conserved beyond *Andalucia* (Additional file 4: Table S3 (C)). The broad although patchy taxonomic distribution of the protein trio (Fig. 4) suggests that the whole system was part of the mitochondrion of LECA. Further work will be required to illuminate the function of the system and to explain why it was lost multiple times during eukaryote evolution.

#### Regulation

We identified several putative mitochondrial proteins that may have regulatory functions in mitochondria. These candidates include aarF domain-containing and serine/arginine-rich protein kinases (SRPK) as well as a serine/threonine phosphatase 2C. We retrieved an apoptosis-inducing factor (AIF) ortholog, which in mammalian cells acts both as a type 2 NADH oxidoreductase and as a regulator of apoptosis [203]. Finally, we found three RCC1 (regulator of chromosome condensation 1) domain-containing proteins, which we designate AgRCC1_1, AgRCC1_2, and AgRCC1_3. These proteins and the aarF domain-containing protein kinase (above) have homologs in *Acanthamoeba*, strongly predicted to be targeted to mitochondria and identified as mitochondrial by MS [10].

### Unknown function (J)

In other protists, a substantial proportion of mitochondrial proteins verified by MS data have no known function: e.g., ~ 20% in *A. castellanii* [10] vs. ~ 45% in *T. thermophila* [9]. In the present study, about 30% (261) of bioinformatically identified candidate mitochondrial proteins are in the “unknown function” class. About 40% (106) of these predicted proteins contain one or more conserved domains, but a specific function cannot be assigned. The other 60% (155) contain no putative conserved domains, with more than half of this group retrieving no significant hits at all by BLASTp against the non-redundant protein sequences at NCBI. However, their genes are transcribed, so are presumably functional. Direct analysis (e.g., by MS) will obviously be required to assess how many of these candidates, particularly those having a moderate or low probability of mitochondrial targeting, are truly mitochondrial in location in *Andalucia.*

In BLASTp searches, 41 of the *Andalucia* proteins of unknown function retrieve conserved homologs of similarly undefined function at an *E*-value < 10^− 20^. To the extent that proteins in this category can be definitively shown to be localized to mitochondria in *Andalucia* and other eukaryotes, their function (presumably conserved) will be of particular interest.

Finally, three of the putative mitochondrial proteins of unknown function in *Andalucia* (ANDGO_07227, _07397 and _07436) proved to be closely related paralogs and to belong to a seven-member gene family. At the amino acid level, members of this family are > 80% identical to one another but contain no evident conserved domain(s) that might hint at function. Nor do they retrieve any potential homologs in BLASTp searches, even at high *E*-values (10^2^).

## Discussion

### *Andalucia godoyi*: a pivotal reference point for comparative mitoproteome research

*Andalucia godoyi* is part of a comparative jakobid genome project (details to be published elsewhere) and the genes and proteins characterized here—the first for the nuclear genome of a jakobid—will serve as a starting point for comparative genome analyses across this clade as well as with other early diverging protists. The mitoproteome data reported here represent the first comprehensive analysis for a non-parasitic eukaryote phylogenetically distant from species whose mitoproteomes have been analyzed in depth to date. As such, our study provides a basis for comparative analysis of mitochondrial proteome structure, function and evolution within Discoba, and more broadly.

Currently, the most detailed mitochondrial proteomes are available from animals [12–15], fungi [16–18], and plants [19–22]. Among protists, such studies have concentrated on animal parasites such as apicomplexans [204] and kinetoplastids [24] or have involved other eukaryotic microbes, such as *T. thermophila* [9] and *C. reinhardtii* [23], which have highly derived and fast evolving mitochondrial genomes [205]. Another focus has been on anaerobic protists containing mitochondrion-related organelles (MROs), in which the mitochondrial genome has been substantially truncated or eliminated altogether, and in which the organellar proteome is similarly drastically reduced [41, 206–212]. Our detailed analysis of the mitoproteome of *A. godoyi*, like that of *A. castellanii* [10], is one of the few to date bearing on a protist that is a free-living heterotrophic aerobe whose mitochondrial genome is “ancestral,” i.e., minimally derived [213].

## Inferences about the LECA mitoproteome

In its essential features, the inferred *A. godoyi* mitochondrial proteome is as complex as that in multicellular animals, plants, and fungi: almost all of the expected elements that constitute the conserved core of mitochondrial structure and function are present. *Andalucia* encodes orthologs of at least 145 proteins involved in energy metabolism (PDH, TCA cycle, ETC-OxPhos), including at least 41 proteins involved in the assembly of complexes CI to CV. Some 31 of these proteins (21%) are encoded by the *Andalucia* mitochondrial genome. At least 39 proteins (4 encoded by *Andalucia* mtDNA) are implicated in various aspects of DNA replication, repair, transcription, and RNA processing. The *Andalucia* mitochondrial translation system comprises at least 149 proteins (29—mostly mtRPs—encoded in mtDNA) with a full complement of initiation, elongation, and termination factors, aminoacyl-tRNA synthetases, SSU and LSU ribosomal proteins, as well as > 20 tRNA and rRNA modification enzymes and a number of ribosome assembly proteins. Membrane-associated complexes such as ERMES and MICOS are present, as are various conserved protein import systems elucidated in other organisms (e.g., TOM, TIM22, TIM23, SAM, MIA). *Andalucia* encodes full pathways for highly conserved mitochondrial metabolic functions such as Fe-S cluster biosynthesis, glycine cleavage, branched chain amino acid degradation, ubiquinone biosynthesis, fatty acid oxidation, and cardiolipin biosynthesis, as well as > 100 assorted proteins involved in various other amino acid, nucleotide, lipid, and coenzyme metabolic pathways. Finally, *Andalucia* encodes at least 32 orthologs of mitochondrial proteins involved in protein folding, processing, and degradation as well as ROS metabolism and regulation. The inventory of components making up certain complexes or pathways is virtually the same in *Andalucia* as in a mammalian mitochondrion: key components such as the ETC and the ribosome appear to have many or all of the additional proteins that distinguish a mitochondrial complex from its bacterial counterpart. The *Andalucia* mitoproteome is therefore decidedly not a simpler—i.e., transitional—type. These observations reinforce the view that LECA had a highly developed and broadly functional mitochondrion [11].

Nevertheless, certain complexes do appear to be simpler in *Andalucia* (e.g., MICOS, TOM, TIM22; Fig. 6), suggesting either that some components have been secondarily lost or that further subunits were incorporated into these complexes in a lineage-specific fashion later in evolution (see, e.g., [149, 161]). This apparent simplicity compared to animals, fungi, and land plants (primarily multicellular eukaryotes) is not unique to *Andalucia*, but applies to other protists, as well. For example, *A. godoyi* and *A. castellanii* appear to lack precisely the same TOM and TIM22 components (Table 4). Assembly pathways (e.g., for the respiratory complexes) may also be simpler, as not all of the factors identified in mammalian or fungal systems were found in *Andalucia*. In addition, several expected regulatory proteins appear to be missing (for PDH and glycine cleavage, e.g.), as do several mtRPs. Notably, we could not detect CV subunits e and g, important for ATP synthase dimerization. Further work will be needed to establish whether alternative proteins substitute for expected subunits and factors that appear to be missing from the *Andalucia* mitoproteome.

In contrast to all other eukaryotes (including other discobans), in which a nucleus-encoded, phage-like, single-subunit enzyme functions as the mitochondrial RNAP, mitochondrial transcription in jakobids is mediated by a mtDNA-encoded, bacterial-type, multi-subunit RNAP. We were therefore particularly interested in the possibility that the jakobid nuclear genome might also encode a phage-like RNAP in addition to the organelle-encoded RNAP, as in the case of chloroplast gene expression in land plants [32]. However, we found no evidence, in either the genome or the transcriptome data, of a phage-like RNAP sequence, which also applies to other jakobids (unpublished observations). While absence is always difficult to prove definitively, in this instance both the genome and transcriptome were deeply sequenced, and there is sufficient conservation in the phage-like mitochondrial RNAP sequence that it should have been readily retrieved, if present. This observation has implications for how we view the origin and subsequent evolution of the mitochondrial transcription system, and how and when the transition from mtDNA-encoded bacterial-type RNAP to nucleus-encoded phage-like RNAP occurred during mitochondrial evolution.

## Primitive features of the *Andalucia godoyi* mitoproteome

In 1975, in a paper in *Nature*, John and Whatley [214] demonstrated that the alphaproteobacterium, *Paracoccus denitrificans*, “resembles a mitochondrion more closely than do other bacteria, in that it effectively assembles in a single organism those features of the mitochondrial respiratory chain and oxidative phosphorylation which are otherwise distributed at random among most other aerobic bacteria.” In a similar fashion, *Andalucia godoyi* might be considered the eukaryote whose mitochondrion likely resembles the LECA mitochondrion more closely than does the mitochondrion in other eukaryotes studied to date, in that it retains a collection of ancestral bacterial features that have been lost or have a highly punctate distribution within eukaryotes. These features include (1) a bacterial-type GreA/GreB transcription elongation/transcript cleavage factor, complementing the bacterial-type mitochondrial RNAP, (2) RnpA, the protein component of bacterial RNase P, (3) a bacterial-type RecA, (4) an FtsZ-Min pathway, involved in cell division in bacteria [137], (5) a type 2 protein secretion system (T2SS) [171], (6) a complete twin-arginine translocation (TAT) pathway, (7) a Ccm cytochrome *c* biogenesis system, notably including the CcmD subunit, (8) bacterial-type tmRNA-SmpB, (9) a three-component aerobic-type rubrerythrin system, and (10) HslUV protease. An additional ancestral feature might be the bacterial-type Pol I mitochondrial DNAP that is unrelated to the common mitochondrial POP DNAP (Fig. 3); however, further analyses are needed to rule out the possibility that this protein represents a more recent acquisition in the *Andalucia* lineage via LGT from a bacterial source.

Despite the foundational bacterial character of such mitochondrial complexes as respiratory chain CI to CV and the mitoribosome, the accumulated data presented here and elsewhere increasingly draw attention to the substantial changes in the mitoproteome that were already in place by the time LECA emerged. The *Andalucia* mitoribosome highlights this point particularly well. The mtDNA-encoded *Andalucia* LSU, SSU, and 5S rRNA components are highly bacterial in sequence and secondary structure [30], and here we identified orthologs of all but one of the 21 proteins found in a typical proteobacterial SSU (S20 was inferred by Desmond et al. [114] to have been absent from the LECA mitoribosome), and all but one of 33 proteobacterial LSU ribosomal proteins (L30 missing). However, we also identified orthologs of an additional 8 SSU and 10 LSU mtRPs that were recruited during the transition from endosymbiont to LECA mitochondrion. Why these additional proteins are needed in the mitoribosome compared to its bacterial antecedent is not clear, but presumably has to do with requirements peculiar to the mitochondrial translation system (e.g., one LSU mtRP, Mba1/L45, has been identified as a receptor for binding to the MIM). Conversely, other bacterial complexes/processes have been considerably simplified in mitochondria, notably DNA replication, where a Pol A-family DNAP replaced the multi-subunit Pol C-family replisome and the Twinkle helicase-primase replaced the multi-subunit DnaABC initiation complex.

In considering the comparative mitoproteome data that are now available, some of the most striking departures from the ancestral LECA pattern have occurred in lineages leading specifically to or within opisthokonts. These departures include (1) replacement of the Pol A-family DNAP with a phage-like DNAP γ in animals and fungi [78], (2) loss of the TAT protein transport system, (3) loss of the two γ-type carbonic anhydrase subunits of CI, (4) substitution of System I cytochrome *c* maturation proteins (Ccm) by a single holo cytochrome *c* synthase enzyme (HCCS [215]), and (5) replacement of the ancestral tRNA CCA nucleotidyltransferase (eCCA) enzyme by a LGT-acquired aCCA version [95].

Finally, in the context of mitochondrial genome and proteome evolution, it is striking that on the one hand Discoba contains the least derived, most bacteria-like mitochondrial genomes yet described (within jakobids), and on the other hand, some of the most highly derived, least bacteria-like mitochondrial genomes known (within euglenozoans [216, 217]. Comparison of jakobid and euglenozoan mitochondrial proteomes, as they become available, will be an important tool for tracing post-LECA mitochondrial evolution within and between these specific clades.

## Conclusions

Considering the relatively high number of ancestral bacteria-like characteristics—both mitochondrion- and nucleus-encoded—that the *A. godoyi* mitochondrion retains, juxtaposed with the fact that in its essential features the inferred mitochondrial proteome of this protist is as complex as that in multicellular animals, plants, and fungi, the *Andalucia* mitochondrion likely resembles the LECA mitochondrion more closely than does the mitochondrion in any other eukaryote studied to date. As genome sequence and mitochondrial proteome data have become more widely available, the strikingly punctuate phylogenetic distribution of different mitochondrial components has become increasingly evident, emphasizing that the pathways of mitoproteome evolution are likely complex and lineage-specific. Unraveling this complexity will require comprehensive comparative analyses of mitochondrial proteomes from a phylogenetically broad range of eukaryotes, especially protists. The systematic in silico approach described here offers a valuable adjunct to direct proteomic analysis (e.g., via MS), particularly in cases where the latter approach is constrained by sample limitation or purity (leading to false positive results) or other considerations, such as low-abundance mitochondrial proteins or mitochondrial proteins only expressed under certain growth conditions (false negative results).

## Methods

### Growth of *A. godoyi* and extraction and purification of DNA for sequencing

*A. godoyi* strain And28 [26] was kindly provided by A. Simpson (Dalhousie University). The large variety of food bacteria that is present in the original strain isolate was reduced by repeated dilution in growth medium plus live *Enterobacter aerogenes* (ATCC 13048) as a food source, so as to retain only a few jakobid cells. Isolates with few bacterial contaminants were identified and are being kept for long-term storage under liquid nitrogen. For DNA and RNA purification, aliquots of these isolates were added to fresh medium containing pre-cultured live *E. aerogenes* as a food source. Detailed recipes for the media are described at [218]. Cultures (500 mL) in 2.5-L Erlenmeyer flasks were gently shaken at 22 °C and daily supplemented with live bacteria. Cells were harvested by centrifugation in the early stationary growth phase (after 2-5 days), at a point when most food bacteria have been consumed.

### DNA purification

Harvested cells were lysed in a Tris-EDTA buffer containing 0.2% SDS plus 100 μg/ml proteinase K, dialyzed for 24 h against the same buffer, and then further purified by CsCl-*bis*benzimide equilibrium gradient centrifugation [219].

### DNA sequencing, assembly of reads

Several shotgun and paired-end fragment libraries (3 kb and 8 kb, respectively) were prepared using the Rapid Library Preparation Method and Paired End Rapid Library Preparation (Roche). Purified genomic DNA was sheared using either by nebulization (for the shotgun library) or using the Digilab Hydroshear device set up for 20 cycles at a calibrated speed 12 with standard shearing. Shotgun fragment libraries with an average fragment length of 500–1200 bp were subsequently ligated to adapters and amplified by emulsion PCR on beads using CPB ratio 7 (DNA copy number per bead). The enriched beads were recovered from the emulsion, applied to a large-region version of PicoTiter Plate and run on the 454 GS FLX+ sequencer using XL+ chemistry to generate 1.35 Gb of sequencing data. GS Run Processor 2.6 (Roche) was used for standard image and signal processing. Roughly 3 million reads, amounting to 1.16 Gb of shotgun sequences and 185 Mb of paired-end sequences, were generated and assembled using the Newbler v2.6 assembler (Roche). Scaffolds were filtered to remove sequences representing obvious bacterial contaminants (BLASTn against NCBI bacterial genomes), and contiguity of the assembly was improved by manual joining of scaffolds in cases with evident read pairing and sequence overlap that were missed by Newbler. Illumina reads (~ 20 million 94-bp paired-end reads) were generated and used for correcting errors that are known to occur with 454 sequences (single-nucleotide indels and substitution; difficulties in identifying the exact number of nucleotides in homopolymer stretches). Correction was achieved by mapping the Illumina reads onto the 454-based assembly with the aid of the Burroughs-Wheeler Alignment tool [220]. The main assembly used in this study comprises 66 nuclear genome scaffolds (20.088 Mb, including 150 gaps), omitting several short poorly assembled subtelomeric scaffolds containing primarily transposon/viral-like sequences.

Benchmarking universal single-copy orthologs (BUSCO [221]) reveals that the *Andalucia godoyi* genome assembly contains most (88.8%) of the 303 eukaryotic orthologous genes in OrthoDB version 9.1 [222]. Ten gene models were reported as fragmented; however, the high RNA-Seq read coverage in all cases (minimum 170) suggests that their simple structures (only one model has a single intron) are complete. The remaining 24 of the 303 orthologous genes are apparently missing, which may be partly due to the score cutoffs imposed by the BUSCO tool based on a taxonomic sampling that is evolutionarily distant from *A. godoyi*. A profile HMM search (HMMER version 3.2.1 [223]) of the missing 24 orthologous genes reveals that 11 are likely present (*E*-values ranging from 1.6e−79 to 5.5e−5). The remaining 13 potentially missing orthologs are on average shorter (mean 210 amino acids, S.D. 120) than the other 290 (mean 294 amino acids, S.D. 212). A fraction of the 13 may not have been identified owing to the greater difficulty in inferring shorter genes [224], whereas others may have been lost during the evolution of *A. godoyi*.

### Extraction and purification of RNA for preparation of an RNA-Seq library

*A. godoyi* was cultured non-axenically in partly filled 50-mL Falcon tubes at 18 °C, in Page’s Amoeba Saline (2.37 mM Na_2_HPO_4_, 2 mM KH_2_PO_4_, 0.03 mM MgSO_4_•7H_2_O, 0.05 mM CaCl_2_•2H_2_O, 4.1 mM NaCl) supplemented with 1/300 v/v lysogeny broth medium (10 g/L tryptone, 10 g/L NaCl, 5 g/L yeast extract). Total RNA was extracted from high-density cultures using TRI Reagent® (Molecular Research Centre, Inc.) following the manufacturer’s instructions for cells grown in suspension.

An aliquot of high-quality total RNA was sent to the National Center for Genome Resources (NCGR, Santa Fe, NM, U.S.A.) for standard Illumina RNA-Seq library preparation and Illumina HiSeq2000 paired-end sequencing. This sequencing was funded by the Moore Foundation as part of the Marine Microbial Eukaryote Transcriptome Sequencing Project (sample ID MMETSP0130), resulting in 14,240,900 reads in total.

### Transcriptome assembly

Initially, reads from two separate runs were assembled separately de novo using Trinity v. r2011-07-13 [225], with a default k-mer size of 25 and minimum contig length of 200 bp. In a second round, adapters in RNA-Seq reads were removed with Trimmomatic [226], then quality-clipped and error-corrected using Rcorrector [227]. Transcripts were again assembled from RNA-Seq reads using Trinity and aligned to the genome assembly using PASA [228] following the instructions listed on the respective GitHub wiki pages [229, 230].

### Structural and functional genome annotation

Design of the structural genome annotation pipeline employed here was based on the one outlined by [231], with a major difference in the order of operations and minor differences in the gene prediction software used. Rather than performing various steps of gene modeling in parallel, the repeat discovery step was performed first. Further, Spaln [232] rather than GeneWise plus tBLASTn was used for protein sequence similarity searches in a local version of the UniProtKB and Protozoan RefSeq data sets (downloaded November 8, 2017) against the genome. The ab initio predictors employed were Augustus [233], Snap [234], Genemark [235], and CodingQuarry [236]. For the functional gene annotation, we followed the procedure outlined in [231]. Expert mitoproteome curations were applied to the respective gene models in the current version of the genome annotation (see below).

### In silico approach to identification and manual curation of candidate mitochondrial proteins

BLASTp searches were carried out against protein sequences predicted from *A. godoyi* genome sequence data, with tBLASTn searches conducted against assembled RNA-Seq data. The primary query set was a manually curated, comprehensive set of mitochondrial proteins from *A. castellanii*, inferred from a combination of direct MS and in silico analysis [10, 25]. In a number of cases, heterologous searches were supplemented with authenticated mitochondrial proteins from other organisms. For certain small, highly divergent proteins that were not identified by the above protocol, HMM searches were carried out with HMMER v3.1b2 [237], typically utilizing profile HMMs built from seed alignments provided by the Pfam database [238]. Programs used to further examine candidate proteins of unknown function included HHpred [239] and Phyre2 [240]. In HHpred searches, default settings were employed for building a multiple sequence alignment of the query and homologs (the latter identified by the iterative HHblits-based algorithm implemented in the search procedure). Four HMM databases were searched in parallel: PDB_mmCIF70, COG_KOG_v1.0, Pfam-A_v32.0, and NCBI_Conserved_Domains (CD)_v3.16. Phyre2 was run in the “normal” modeling mode.

Various mitochondrial-targeting prediction programs were used to infer the presence of an N-terminal MTS. Primary screens were carried out with TargetP 1.1 [241]; secondary screens used MitoProt [242], Predotar 1.04 [243], and PSORT II [244]. Tertiary screens employed PredSL [245] and MitoFates [246].

To assess the overall likelihood of mitochondrial targeting, a probability metric (P) was defined as the average of TargetP, MitoProt, Predotar, and PSORT probabilities. In contrast to PredSL and MitoFates, these four programs consistently predicted mitochondrial targeting for a set of proteins in three ubiquitous matrix pathways: TCA cycle, Fe-S cluster biosynthesis, and branched chain amino acid degradation (Additional file 2: Table S2). The results were sorted into three bins, reflecting the relative targeting strengths: strong (P = 75–95), moderate (P = 50–74), and weak (P = 30–49). We consider the last two bins to comprise “probable” and “possible” mitochondrial candidates, respectively. To be considered a mitochondrial candidate in our analysis, we required that a protein have P ≥ 30 *and* be predicted to be mitochondrially targeted by at least three out of the six targeting prediction programs used. Regardless of P value, candidates were inferred to be mitochondrial if identified orthologs in other eukaryotes are known to localize exclusively to and function in this organelle. Certain proteins that appear to lack a MTS (P < 30) were nevertheless considered to be candidates on other grounds, e.g., mitochondrial membrane proteins and MC proteins, which are known to lack an N-terminal MTS in other eukaryotes [146].

For each candidate protein, the predicted gene model was verified or corrected, as necessary; introns, if present, were identified and sized; and the N-terminus was assigned, all by comparison of genomic and RNA-Seq data. Correspondence between genomic and RNA-Seq data was excellent throughout, with only a very small number of single nucleotide polymorphisms encountered, most of which were silent third-position codon changes (indicating either that the *A. godoyi* sample used in this study comprised a non-clonal haploid population, or was a diploid that originated from two genetically very close mating partners). In the overwhelming number of cases, RNA-Seq data covered the complete coding sequence; however, an in-frame upstream termination codon was not always present in assembled transcripts and in these instances was instead inferred from genomic sequence.

In a small number of cases, the reading frame remained open upstream of the originally assigned initiation codon but the corresponding RNA-Seq data did not extend 5′ to the beginning of the putative N-terminal extension. In these cases, the upstream initiation codon was assigned as the protein start where this resulted in the prediction of an N-terminal MTS.

A number of proteins not known to have a mitochondrial localization but functioning instead in the nucleus or cytosol nevertheless were predicted to have strong MTSs. Examples include cytosolic ribosomal proteins, histones, and spliceosomal proteins. These presumptive false positives (10–15% of the total retrieved by TargetP screening) are generally RNA-binding proteins, so it is likely that their particular amino acid composition spuriously predicts mitochondrial targeting. These proteins were excluded from the list of mitochondrial candidates, as were any other candidates known to function exclusively elsewhere in the cell.

### Phylogenetic analyses

Sets of sequences were aligned using Mafft v.7.4 [247] with default parameters and subsequently trimmed using trimAl v1.4 [248] by removing positions with more than 50% of missing characters. Trimmed alignments were analyzed using RAxML v.8.2 [249] in a combined rapid bootstrap analysis and search for best-scoring maximum likelihood (ML) tree (“-f a” option; 100 bootstrap replicates) under the PROTGAMMALG model. With alignments that included a large number of sequences, tree inference was performed using FastTree [250] with default parameters.

## Supplementary information

**Additional file 1:Table S1.** Annotated categories of predicted *A. godoyi* mitochondrial proteins. Excel file listing candidate mitochondrial proteins from *A. godoyi* and relevant information about them, including both amino acid and coding sequences. The Key tab defines various aspects of the presentation (calculation of probability of mitochondrial targeting, color coding, etc.). Functional categories as defined in Fig. 1 are organized in subsequent tabs (A) through (J), with relevant statistical data compiled in the final (Statistics) tab. (XLSX 808 kb)

**Additional file 2: Table S2.** Assessment of mitochondrial targeting. Excel file listing predicted mitochondrial targeting probabilities for three classes of mitochondrial matrix proteins assessed with six targeting programs. (XLSX 15 kb)

**Additional file 3: Figure S1.** Phylogenetic analysis of selected *A. godoyi* mitochondrial protein sequences: (A) pyruvate kinase; (B) RecA; (C) SmpB. See Methods section for details of the analysis. The best ML trees are shown without support values. *A. godoyi* sequences and other eukaryotic sequences are coloured red and grey, respectively. Cyanobacteria and Alpha and Beta-Gamma Proteobacteria are coloured green and blue, respectively. Other bacterial sequences are coloured black. (PDF 307 kb)

**Additional file 4: Table S3.** Mitochondrial proteins in eukaryotes. Excel file listing selected *A. godoyi* mitochondrial proteins and their orthologs in other eukaryotes. (A) mitochondrial RecA; (B) mitochondrial SmpB (nucleus-encoded) and tmRNA (specified by mtDNA-encoded *ssrA* gene); (C) aerobic-type rubrerythrin and associated proteins (DUF3501, RFO). (XLSX 23 kb)

**Additional file 5: Figure S2.** A novel patchily distributed mitochondrial protein of the DegT/DnrJ/EryC1/StrS aminotransferase family. (A) Phylogenetic analysis of a selection of protein sequences of the DegT/DnrJ/EryC1/StrS aminotransferase family including the *Andalucia godoyi* protein ANDGO_00275.mRNA.1 and its relatives from other eukaryotes (identified by a near-exhaustive searching of available eukaryotic genomic and transcriptomic data). The tree was inferred using FastTree (default parameters) from an alignment of 372 amino acid positions. Black dots denote bootstrap support ≥91%. Branches in black correspond to sequences from prokaryotes, those in red are eukaryotic (a detailed view of the eukaryotic clade is provided at the top). (B) ANDGO_00275.mRNA.1 and its three eukaryotic relatives exhibit an N-terminal extension compared to their prokaryotic homologs (top) and the four proteins are predicted as mitochondrial by most tools employed (bottom; scores of four prediction tools are provided, with only PSORT II prediction for the *T. trahens* protein favouring cytosolic localization over the mitochondrial one). (PDF 439 kb)

**Additional file 6: Table S4.** Analysis of presumptive orthologs in the ‘General Metabolism’ category (G), *A. godoyi* vs. *A. castellanii.* Excel file listing 61 *A. godoyi* proteins predicted to be targeted to mitochondria [Ago(+)] and functioning in various metabolic pathways, compared to their orthologs from *A. castellanii* [Aca(MS+): direct identification (by MS); Aca(Bio): in silico identification (no supporting MS data); Aca(MS-): no MS data supporting a mitochondrial localization]. (XLSX 19 kb)

**Additional file 7: Figure S3.** Phylogenetic analysis of (A) RFO and (B) DUF3501 proteins. ML trees (RAxML, PROTGAMMALG) are shown, with branch support values (rapid bootstraps) indicated when ≥51%. The root is placed arbitrarily. The RFO tree (A) is based on an alignment of 365 amino acid positions. The sequence from *Ancoracysta twista* is only partial, possibly explaining why it does not cluster together with sequences from other eukaryotes. Note the sequence from the gammaproteobacterium *Arhodomonas aquaeolei* nested among alphaproteobacterial sequences, suggesting a possible HGT event. The DUF3501 tree (B) is based on an alignment of 128 amino acid positions. (PDF 864 kb)

## Abbreviations

DMT: Dimethyltransferase
DNAP: DNA polymerase
EGT: Endosymbiotic gene transfer
ETC: Electron transport chain
Fe-S: Iron-sulfur
GPDH: Glycerol-3-phosphate dehydrogenase
HMM: Hidden Markov model
IMS: Intermembrane space
LECA: Last eukaryotic common ancestor
LGT: Lateral gene transfer
LSU: Large ribosomal subunit
MAO: Monoamine oxidase
MC: Mitochondrial carrier
MIM: Mitochondrial inner membrane
Mitoproteome: Mitochondrial proteome
Mitoribosome: Mitochondrial ribosome
ML: Maximum likelihood
MOM: Mitochondrial outer membrane
MS: Mass spectrometry, mass spectrometric
mtDNA: Mitochondrial DNA
mtRP: Mitochondrial ribosomal protein
MTS: Mitochondrial targeting sequence
OxPhos: Oxidative phosphorylation
P: Probability metric
PDH: Pyruvate dehydrogenase
PDP: Pyruvate dehydrogenase phosphatase
PTH: Peptidyl-tRNA hydrolase
RBR: Rubrerythrin
RFO: Fe-S oxidoreductase
RNAP: RNA polymerase
ROS: Reactive oxygen species
SSU: Small ribosomal subunit
TCA: Tricarboxylic acid
TIM: Translocase of the inner mitochondrial membrane
TOM: Translocase of the outer mitochondrial membrane

## References

[1] Roger AJ, Muñoz-Gómez SA, Kamikawa R (2017). The origin and diversification of mitochondria. Curr Biol.

[2] Gray MW (2015). Mosaic nature of the mitochondrial proteome: implications for the origin and evolution of mitochondria. Proc Natl Acad Sci U S A.

[3] Gabaldón T, Huynen MA (2003). Reconstruction of the proto-mitochondrial metabolism. Science..

[4] Gabaldón T, Huynen MA (2007). From endosymbiont to host-controlled organelle: the hijacking of mitochondrial protein synthesis and metabolism. PLoS Comput Biol.

[5] Wang Z, Wu M (2014). Phylogenomic reconstruction indicates mitochondrial ancestor was an energy parasite. PLoS One.

[6] Martijn J, Vosseberg J, Guy L, Offre P, Ettema TJG (2018). Deep mitochondrial origin outside the sampled alphaproteobacteria. Nature..

[7] Esser C, Martin W, Dagan T (2007). The origin of mitochondria in light of a fluid prokaryotic chromosome model. Biol Lett.

[8] Ku C, Nelson-Sathi S, Roettger M, Garg S, Hazkani-Covo E, Martin WF (2015). Endosymbiotic gene transfer from prokaryotic pangenomes: inherited chimerism in eukaryotes. Proc Natl Acad Sci U S A.

[9] Smith DGS, Gawryluk RMR, Spencer DF, Pearlman RE, Siu KWM, Gray MW (2007). Exploring the mitochondrial proteome of the ciliate protozoon *Tetrahymena thermophila*: direct analysis by tandem mass spectrometry. J Mol Biol.

[10] Gawryluk RMR, Chisholm KA, Pinto DM, Gray MW (2014). Compositional complexity of the mitochondrial proteome of a unicellular eukaryote (*Acanthamoeba castellanii*, supergroup Amoebozoa) rivals that of animals, fungi, and plants. J Proteome.

[11] Koumandou VL, Wickstead B, Ginger ML, van der Giezen M, Dacks JB, Field MC (2013). Molecular paleontology and complexity in the last eukaryotic common ancestor. Crit Rev Biochem Mol Biol.

[12] Taylor SW, Fahy E, Zhang B, Glenn GM, Warnock DE, Wiley S, Murphy AN, Gaucher SP, Capaldi RA, Gibson BW (2003). Characterization of the human heart mitochondrial proteome. Nat Biotechnol.

[13] Pagliarini DJ, Calvo SE, Chang B, Sheth SA, Vafai SB, Ong S-E, Walford GA, Sugiana C, Boneh A, Chen WK (2008). A mitochondrial protein compendium elucidates complex I disease biology. Cell..

[14] Li J, Cai T, Wu P, Cui Z, Chen X, Hou J, Xie Z, Xue P, Shi L, Liu P (2009). Proteomic analysis of mitochondria from *Caenorhabditis elegans*. Proteomics..

[15] Muthye V, Lavrov DV (2018). Characterization of mitochondrial proteomes of nonbilaterian animals. IUBMB Life.

[16] Sickmann A, Reinders J, Wagner Y, Joppich C, Zahedi R, Meyer HE, Schönfisch B, Perschil I, Chacinska A, Guiard B (2003). The proteome of *Saccharomyces cerevisiae* mitochondria. Proc Natl Acad Sci U S A.

[17] Reinders J, Zahedi RP, Pfanner N, Meisinger C, Sickmann A (2006). Toward the complete yeast mitochondrial proteome: multidimensional separation techniques for mitochondrial proteomics. J Proteome Res.

[18] Vögtle FN, Wortelkamp S, Zahedi RP, Becker D, Leidhold C, Gevaert K, Kellermann J, Voos W, Sickmann A, Pfanner N (2009). Global analysis of the mitochondrial N-proteome identifies a processing peptidase critical for protein stability. Cell..

[19] Heazlewood JL, Howell KA, Whelan J, Millar AH (2003). Towards an analysis of the rice mitochondrial proteome. Plant Physiol.

[20] Heazlewood JL, Tonti-Filippini JS, Gout AM, Day DA, Whelan J, Millar AH (2004). Experimental analysis of the Arabidopsis mitochondrial proteome highlights signaling and regulatory components, provides assessment of targeting prediction programs, and indicates plant-specific mitochondrial proteins. Plant Cell.

[21] Huang S, Taylor NL, Narsai R, Eubel H, Whelan J, Millar AH (2009). Experimental analysis of the rice mitochondrial proteome, its biogenesis, and heterogeneity. Plant Physiol.

[22] Huang S, Shingaki-Wells RN, Taylor NL, Millar H (2013). The rice mitochondria proteome and its response during development and to the environment. Front Plant Sci.

[23] Atteia A, Adrait A, Brugière S, Tardif M, van Lis R, Deusch O, Dagan T, Kuhn L, Gontero B, Martin W (2009). A proteomic survey of *Chlamydomonas reinhardtii* mitochondria sheds new light on the metabolic plasticity of the organelle and on the nature of the α-proteobacterial mitochondrial ancestor. Mol Biol Evol.

[24] Panigrahi AK, Ogata Y, Zíková A, Anupama A, Dalley RA, Acestor N, Myler PJ, Stuart KD (2009). A comprehensive analysis of *Trypanosoma brucei* mitochondrial proteome. Proteomics..

[25] Gawryluk RMR, Chisholm KA, Pinto DM, Gray MW (1817). Composition of the mitochondrial electron transport chain in *Acanthamoeba castellanii*: structural and evolutionary insights. Biochim Biophys Acta.

[26] Lara E, Chatzinotas A, Simpson AGB (2006). *Andalucia* (n. gen.)—the deepest branch within jakobids (Jakobida; Excavata), based on morphological and molecular study of a new flagellate from soil. J Eukaryot Microbiol.

[27] Hampl V, Hug L, Leigh JW, Dacks JB, Lang BF, Simpson AGB, Roger AJ (2009). Phylogenomic analyses support the monophyly of Excavata and resolve relationships among eukaryotic “supergroups”. Proc Natl Acad Sci U S A.

[28] Derelle R, Torruella G, Klimeš V, Brinkmann H, Kim E, Vlček Č, Lang BF, Eliáš M (2015). Bacterial proteins pinpoint a single eukaryotic root. Proc Natl Acad Sci U S A.

[29] Lang BF, Burger G, O'Kelly CJ, Cedergren R, Golding GB, Lemieux C, Sankoff D, Turmel M, Gray MW (1997). An ancestral mitochondrial DNA resembling a eubacterial genome in miniature. Nature..

[30] Burger G, Gray MW, Forget L, Lang BF (2013). Strikingly bacteria-like and gene-rich mitochondrial genomes throughout jakobid protists. Genome Biol Evol..

[31] Yabuki A, Gyaltshen Y, Heiss AA, Fujikura K, Kim E (2018). *Ophirina amphinema* n. gen., n. sp., a new deeply branching discobid with phylogenetic affinity to jakobids. Sci Rep.

[32] Gray MW, Lang BF (1998). Transcription in chloroplasts and mitochondria: a tale of two polymerases. Trends Microbiol.

[33] Shutt TE, Gray MW (2006). Bacteriophage origins of mitochondrial replication and transcription proteins. Trends Genet.

[34] Karlberg O, Canbäck B, Kurland CG, Andersson SGE (2000). The dual origin of the yeast mitochondrial proteome. Yeast..

[35] Lander N, Chiurillo MA, Bertolini MS, Storey M, Vercesi AE, Docampo R (2018). Calcium-sensitive pyruvate dehydrogenase phosphatase is required for energy metabolism, growth, differentiation, and infectivity of *Trypanosoma cruzi*. J Biol Chem.

[36] Pizzuto R, Paventi G, Atlante A, Passarella S (2010). Pyruvate kinase in pig liver mitochondria. Arch Biochem Biophys.

[37] Saito T, Nishi M, Lim MI, Wu B, Maeda T, Hashimoto H, Takeuchi T, Roos DS, Asai T (2008). A novel GDP-dependent pyruvate kinase isozyme from *Toxoplasma gondii* localizes to both the apicoplast and the mitochondrion. J Biol Chem.

[38] Nakayama T (2012). Ishida K-i, Archibald JM: broad distribution of TPI-GAPDH fusion proteins among eukaryotes: evidence for glycolytic reactions in the mitochondrion?. PLoS One.

[39] Abrahamian M, Kagda M, Ah-Fong AMV, Judelson HS (2017). Rethinking the evolution of eukaryotic metabolism: novel cellular partitioning of enzymes in stramenopiles links serine biosynthesis to glycolysis in mitochondria. BMC Evol Biol.

[40] Río Bártulos C, Rogers MB, Williams TA, Gentekaki E, Brinkmann H, Cerff R, Liaud M-F, Hehl AB, Yarlett NR, Gruber A (2018). Mitochondrial glycolysis in a major lineage of eukaryotes. Genome Biol Evol.

[41] Leger MM, Eme L, Hug LA, Roger AJ (2016). Novel hydrogenosomes in the microaerophilic jakobid *Stygiella incarcerata*. Mol Biol Evol.

[42] Müller M, Mentel M, van Hellemond JJ, Henze K, Woehle C, Gould SB, Yu R-Y, van der Giezen M, Tielens AGM, Martin WF (2012). Biochemistry and evolution of anaerobic energy metabolism in eukaryotes. Microbiol Mol Biol Rev.

[43] Carroll J, Fearnley IM, Skehel JM, Shannon RJ, Hirst J, Walker JE (2006). Bovine complex I is a complex of 45 different subunits. J Biol Chem.

[44] Brandt U (2006). Energy converting NADH:quinone oxidoreductase (complex I). Annu Rev Biochem.

[45] Hirst J, Carroll J, Fearnley IM, Shannon RJ, Walker JE (1604). The nuclear encoded subunits of complex I from bovine heart mitochondria. Biochim Biophys Acta.

[46] Balsa E, Marco R, Perales-Clemente E, Szklarczyk R, Calvo E, Landázuri Manuel O, Enríquez José A (2012). NDUFA4 is a subunit of complex IV of the mammalian electron transport chain. Cell Metab.

[47] Zong S, Wu M, Gu J, Liu T, Guo R, Yang M (2018). Structure of the intact 14-subunit human cytochrome *c* oxidase. Cell Res.

[48] Cardol P (1807). Mitochondrial NADH:ubiquinone oxidoreductase (complex I) in eukaryotes: a highly conserved subunit composition highlighted by mining of protein databases. Biochim Biophys Acta.

[49] Ligas J, Pineau E, Bock R, Huynen MA, Meyer EH (2019). The assembly pathway of complex I in *Arabidopsis thaliana*. Plant J.

[50] Nakagawa T, Maeshima M, Nakamura K, Asahi T (1990). Molecular cloning of a cDNA for the smallest nuclear-encoded subunit of sweet potato cytochrome *c* oxidase. Eur J Biochem.

[51] Pitceathly RDS, Taanman J-W (2018). NDUFA4 (renamed COXFA4) is a cytochrome-*c* oxidase subunit. Trends Endocrinol Metab.

[52] Yip Chui-ying, Harbour Michael E., Jayawardena Kamburapola, Fearnley Ian M., Sazanov Leonid A. (2010). Evolution of Respiratory Complex I. Journal of Biological Chemistry.

[53] Gabaldón T, Rainey D, Huynen MA (2005). Tracing the evolution of a large protein complex in the eukaryotes, NADH:ubiquinone oxidoreductase (complex I). J Mol Biol.

[54] Gawryluk RMR, Gray MW (2010). Evidence for an early evolutionary emergence of γ-type carbonic anhydrases as components of mitochondrial respiratory complex I. BMC Evol Biol.

[55] Valach M, Léveillé-Kunst A, Gray MW, Burger G (2018). Respiratory chain complex I of unparalleled divergence in diplonemids. J Biol Chem.

[56] Guerrero-Castillo S, Baertling F, Kownatzki D, Wessels HJ, Arnold S, Brandt U, Nijtmans L (2017). The assembly pathway of mitochondrial respiratory chain complex I. Cell Metab.

[57] Formosa LE, Dibley MG, Stroud DA, Ryan MT (2018). Building a complex complex: assembly of mitochondrial respiratory chain complex I. Sem Cell Dev Biol.

[58] Signes A, Fernandez-Vizarra E (2018). Assembly of mammalian oxidative phosphorylation complexes I–V and supercomplexes. Essays Biochem.

[59] HGNC. Mitochondrial respiratory chain complex assembly factors. [https://www.genenames.org/cgi-bin/genefamilies/set/645].

[60] HGNC. Mitochondrial complex I assembly complex. [https://www.genenames.org/cgi-bin/genefamilies/set/1387].

[61] Gawryluk RMR, Gray MW (2009). A split and rearranged nuclear gene encoding the iron-sulfur subunit of mitochondrial succinate dehydrogenase in Euglenozoa. BMC Res Notes.

[62] Morales J, Mogi T, Mineki S, Takashima E, Mineki R, Hirawake H, Sakamoto K, Ōmura S, Kita K (2009). Novel mitochondrial complex II isolated from *Trypanosoma cruzi* is composed of 12 peptides including a heterodimeric Ip subunit. J Biol Chem.

[63] Eubel H, Jänsch L, Braun H-P (2003). New insights into the respiratory chain of plant mitochondria. Supercomplexes and a unique composition of complex II. Plant Physiol.

[64] Millar A, Eubel H, Jänsch L, Kruft V, Heazlewood J, Braun H (2004). Mitochondrial cytochrome *c* oxidase and succinate dehydrogenase complexes contain plant specific subunits. Plant Mol Biol.

[65] Huang S, Braun H-P, Gawryluk RMR, Millar AH (2019). Mitochondrial complex II of plants: subunit composition, assembly, and function in respiration and signaling. Plant J.

[66] Marx S, Baumgärtner M, Kannan S, Braun H-P, Lang BF, Burger G (2003). Structure of the bc_1_ complex from *Seculamonas ecuadoriensis*, a jakobid flagellate with an ancestral mitochondrial genome. Mol Biol Evol.

[67] Burke PV, Raitt DC, Allen LA, Kellogg EA, Poyton RO (1997). Effects of oxygen concentration on the expression of cytochrome *c* and cytochrome *c* oxidase genes in yeast. J Biol Chem.

[68] Kwast KE, Burke PV, Poyton RO (1998). Oxygen sensing and the transcriptional regulation of oxygen-responsive genes in yeast. J Exp Biol.

[69] Sanders C, Turkarslan S, Lee D-W, Daldal F (2010). Cytochrome *c* biogenesis: the Ccm system. Trends Microbiol.

[70] He D, Fu C-J, Baldauf SL (2016). Multiple origins of eukaryotic *cox15* suggest horizontal gene transfer from bacteria to jakobid mitochondrial DNA. Mol Biol Evol.

[71] Herwaldt EJ, Rivett ED, White AJ, Hegg EL (2018). Cox15 interacts with the cytochrome *bc*_1_ dimer within respiratory supercomplexes as well as in the absence of cytochrome *c* oxidase. J Biol Chem.

[72] Jett KA, Leary SC (2018). Building the Cu_A_ site of cytochrome *c* oxidase: a complicated, redox-dependent process driven by a surprisingly large complement of accessory proteins. J Biol Chem.

[73] Strogolova V, Hoang NH, Hosler J, Stuart RA (2019). The yeast mitochondrial proteins Rcf1 and Rcf2 support the enzymology of the cytochrome *c* oxidase complex and generation of the proton motive force. J Biol Chem.

[74] Jonckheere AI, Smeitink JAM, Rodenburg RJT (2012). Mitochondrial ATP synthase: architecture, function and pathology. J Inherit Met Dis.

[75] Junge W, Nelson N (2015). ATP synthase. Annu Rev Biochem.

[76] Hahn A, Parey K, Bublitz M, Mills Deryck J, Zickermann V, Vonck J, Kühlbrandt W, Meier T (2016). Structure of a complete ATP synthase dimer reveals the molecular basis of inner mitochondrial membrane morphology. Mol Cell.

[77] Mráček T, Drahota Z, Houštěk J (1827). The function and the role of the mitochondrial glycerol-3-phosphate dehydrogenase in mammalian tissues. Biochim Biophys Acta.

[78] Kaguni LS (2004). DNA polymerase γ, the mitochondrial replicase. Annu Rev Biochem.

[79] Moriyama T, Terasawa K, Fujiwara M, Sato N (2008). Purification and characterization of organellar DNA polymerases in the red alga *Cyanidioschyzon merolae*. FEBS J.

[80] Moriyama T, Terasawa K, Sato N (2011). Conservation of POPs, the plant organellar DNA polymerases, in eukaryotes. Protist..

[81] Hirakawa Y, Watanabe A (2019). Organellar DNA polymerases in complex plastid-bearing algae. Biomolecules..

[82] Xu Z-Q, Dixon NE (2018). Bacterial replisomes. Curr Opin Struct Biol.

[83] Shutt TE, Gray MW (2006). Twinkle, the mitochondrial replicative DNA helicase, is widespread in the eukaryotic radiation and may also be the mitochondrial DNA primase in most eukaryotes. J Mol Evol.

[84] Hofstatter PG, Tice AK, Kang S, Brown MW, Lahr DJG (2016). Evolution of bacterial recombinase a (*recA*) in eukaryotes explained by addition of genomic data of key microbial lineages. Proc Biol Sci.

[85] Janouškovec J, Tikhonenkov DV, Burki F, Howe AT, Rohwer FL, Mylnikov AP, Keeling PJ (2017). A new lineage of eukaryotes illuminates early mitochondrial genome reduction. Curr Biol.

[86] Brown MW, Heiss AA, Kamikawa R, Inagaki Y, Yabuki A, Tice AK, Shiratori T, Ishida K-I, Hashimoto T, Simpson AGB (2018). Phylogenomics places orphan protistan lineages in a novel eukaryotic super-group. Genome Biol Evol..

[87] Lax G, Eglit Y, Eme L, Bertrand EM, Roger AJ, Simpson AGB (2018). Hemimastigophora is a novel supra-kingdom-level lineage of eukaryotes. Nature..

[88] Strassert JFH, Jamy M, Mylnikov AP, Tikhonenkov DV, Burki F (2019). New phylogenomic analysis of the enigmatic phylum Telonemia further resolves the eukaryote tree of life. Mol Biol Evol.

[89] Orlova M, Newlands J, Das A, Goldfarb A, Borukhov S (1995). Intrinsic transcript cleavage activity of RNA polymerase. Proc Natl Acad Sci U S A.

[90] Hillen HS, Temiakov D, Cramer P (2018). Structural basis of mitochondrial transcription. Nat Struct Mol Biol.

[91] Schubot FD, Chen C-J, Rose JP, Dailey TA, Dailey HA, Wang B-C (2001). Crystal structure of the transcription factor sc-mtTFB offers insights into mitochondrial transcription. Protein Sci.

[92] Falkenberg M, Gaspari M, Rantanen A, Trifunovic A, Larsson N-G, Gustafsson CM (2002). Mitochondrial transcription factors B1 and B2 activate transcription of human mtDNA. Nature Genet.

[93] Harris AJ, Goldman AD (2018). Phylogenetic reconstruction shows independent evolutionary origins of mitochondrial transcription factors from an ancient family of RNA methyltransferase proteins. J Mol Evol.

[94] Záhonová K, Füssy Z, Birčák E, Novák Vanclová AMG, Klimeš V, Vesteg M, Krajčovič J, Oborník M, Eliáš M (2018). Peculiar features of the plastids of the colourless alga *Euglena longa* and photosynthetic euglenophytes unveiled by transcriptome analyses. Sci Rep.

[95] Betat H, Mede T, Tretbar S, Steiner L, Stadler PF, Mörl M, Prohaska SJ (2015). The ancestor of modern Holozoa acquired the CCA-adding enzyme from Alphaproteobacteria by horizontal gene transfer. Nucleic Acids Res.

[96] Jovanovic M, Sanchez R, Altman S, Gopalan V (2002). Elucidation of structure–function relationships in the protein subunit of bacterial RNase P using a genetic complementation approach. Nucleic Acids Res.

[97] Lai L, Bernal-Bayard P, Mohannath G, Lai S, Gopalan V, Vioque A (2011). A functional RNase P protein subunit of bacterial origin in some eukaryotes. Mol Gen Genomics.

[98] Small ID, Peeters N (2000). The PPR motif - a TPR-related motif prevalent in plant organellar proteins. Trends Biochem Sci.

[99] Schmitz-Linneweber C, Small I (2008). Pentatricopeptide repeat proteins: a socket set for organelle gene expression. Trends Plant Sci.

[100] Schallenberg-Rüdinger M, Lenz H, Polsakiewicz M, Gott JM, Knoop V (2013). A survey of PPR proteins identifies DYW domains like those of land plant RNA editing factors in diverse eukaryotes. RNA Biol.

[101] Manna S (2015). An overview of pentatricopeptide repeat proteins and their applications. Biochimie..

[102] Tsuboi M, Morita H, Nozaki Y, Akama K, Ueda T, Ito K, Nierhaus KH, Takeuchi N (2009). EF-G2mt is an exclusive recycling factor in mammalian mitochondrial protein synthesis. Mol Cell.

[103] Das G, Varshney U (2006). Peptidyl-tRNA hydrolase and its critical role in protein biosynthesis. Microbiology..

[104] Rosas-Sandoval G, Ambrogelly A, Rinehart J, Wei D, Cruz-Vera LR, Graham DE, Stetter KO, Guarneros G, Söll D (2002). Orthologs of a novel archaeal and of the bacterial peptidyl–tRNA hydrolase are nonessential in yeast. Proc Natl Acad Sci U S A.

[105] Jan Y, Matter M, Pai J-T, Chen Y-L, Pilch J, Komatsu M, Ong E, Fukuda M, Ruoslahti E (2004). A mitochondrial protein, Bit1, mediates apoptosis regulated by integrins and Groucho/TLE corepressors. Cell..

[106] Janssen BD, Hayes CS (2012). The tmRNA ribosome-rescue system. Adv Protein Chem Struct Biol.

[107] Huter P, Müller C, Arenz S, Beckert B, Wilson DN (2017). Structural basis for ribosome rescue in bacteria. Trends Biochem Sci.

[108] Jacob Y, Seif E, Paquet P-O, Lang BF (2004). Loss of the mRNA-like region in mitochondrial tmRNAs of jakobids. RNA..

[109] Duarte I, Nabuurs SB, Magno R, Huynen M (2012). Evolution and diversification of the organellar release factor family. Mol Biol Evol.

[110] García-Guerrero AE, Zamudio-Ochoa A, Camacho-Villasana Y, García-Villegas R, Reyes-Prieto A, Pérez-Martínez X, Hernández G, Jagus R (2016). Evolution of translation in mitochondria. Evolution of the protein synthesis machinery and its regulation.

[111] Hafez M, Burger G, Steinberg SV, Lang F (2013). A second eukaryotic group with mitochondrion-encoded tmRNA. RNA Biol.

[112] Nishimura Y, Tanifuji G, Kamikawa R, Yabuki A, Hashimoto T, Inagaki Y (2016). Mitochondrial genome of *Palpitomonas bilix*: derived genome structure and ancestral system for cytochrome *c* maturation. Genome Biol Evol..

[113] Nagao A, Suzuki T, Katoh T, Sakaguchi Y, Suzuki T (2009). Biogenesis of glutaminyl-mt tRNA^Gln^ in human mitochondria. Proc Natl Acad Sci U S A.

[114] Desmond E, Brochier-Armanet C, Forterre P, Gribaldo S (2011). On the last common ancestor and early evolution of eukaryotes: reconstructing the history of mitochondrial ribosomes. Res Microbiol.

[115] Desai N, Brown A, Amunts A, Ramakrishnan V (2017). The structure of the yeast mitochondrial ribosome. Science..

[116] Heublein M, Burguillos MA, Vögtle FN, Teixeira PF, Imhof A, Meisinger C, Ott M, Fox TD (2014). The novel component Kgd4 recruits the E3 subunit to the mitochondrial α-ketoglutarate dehydrogenase. Mol Biol Cell.

[117] Korepanov AP, Gongadze GM, Garber MB, Court DL, Bubunenko MG (2007). Importance of the 5 S rRNA-binding ribosomal proteins for cell viability and translation in *Escherichia coli*. J Mol Biol.

[118] Kamikawa R, Shiratori T, Ishida K-I, Miyashita H, Roger AJ (2016). Group II intron-mediated *trans* -splicing in the gene-rich mitochondrial genome of an enigmatic eukaryote, *Diphylleia rotans*. Genome Biol Evol..

[119] Frazier AE, Thorburn DR, Compton AG (2019). Mitochondrial energy generation disorders: genes, mechanisms, and clues to pathology. J Biol Chem.

[120] Britton RA (2009). Role of GTPases in bacterial ribosome assembly. Annu Rev Microbiol.

[121] Gehl B, Sweetlove LJ (2014). Mitochondrial Band-7 family proteins: scaffolds for respiratory chain assembly?. Front Plant Sci.

[122] Dallabona C, Marsano RM, Arzuffi P, Ghezzi D, Mancini P, Zeviani M, Ferrero I, Donnini C (2010). Sym1, the yeast ortholog of the MPV17 human disease protein, is a stress-induced bioenergetic and morphogenetic mitochondrial modulator. Hum Mol Genet.

[123] Alonzo JR, Venkataraman C, Field MS, Stover PJ (2018). The mitochondrial inner membrane protein MPV17 prevents uracil accumulation in mitochondrial DNA. J Biol Chem.

[124] Lipper CH, Karmi O, Sohn YS, Darash-Yahana M, Lammert H, Song L, Liu A, Mittler R, Nechushtai R, Onuchic JN (2018). Structure of the human monomeric NEET protein MiNT and its role in regulating iron and reactive oxygen species in cancer cells. Proc Natl Acad Sci U S A.

[125] Matsumoto S, Nakatsukasa K, Kakuta C, Tamura Y, Esaki M, Endo T (2019). Msp1 clears mistargeted proteins by facilitating their transfer from mitochondria to the ER. Mol Cell.

[126] Edmondson DE, Binda C (2018). Monoamine oxidases. Subcell Biochem.

[127] Maggiorani D, Manzella N, Edmondson DE, Mattevi A, Parini A, Binda C, Mialet-Perez J (2017). Monoamine oxidases, oxidative stress, and altered mitochondrial dynamics in cardiac ageing. Oxidative Med Cell Longev.

[128] Oka T, Sayano T, Tamai S, Yokota S, Kato H, Fujii G, Mihara K (2008). Identification of a novel protein MICS1 that is involved in maintenance of mitochondrial morphology and apoptotic release of cytochrome c. Mol Biol Cell.

[129] Wideman JG, Gawryluk RMR, Gray MW, Dacks JB (2013). The ancient and widespread nature of the ER–mitochondria encounter structure. Mol Biol Evol.

[130] Nguyen TT, Lewandowska A, Choi J-Y, Markgraf DF, Junker M, Bilgin M, Ejsing CS, Voelker DR, Rapoport TA, Shaw JM (2012). Gem1 and ERMES do not directly affect phosphatidylserine transport from ER to mitochondria or mitochondrial inheritance. Traffic..

[131] Dimmer KS, Jakobs S, Vogel F, Altmann K, Westermann B (2005). Mdm31 and Mdm32 are inner membrane proteins required for maintenance of mitochondrial shape and stability of mitochondrial DNA nucleoids in yeast. J Cell Biol.

[132] Muñoz-Gómez SA, Slamovits CH, Dacks JB, Wideman JG (2015). The evolution of MICOS: ancestral and derived functions and interactions. Commun Integr Biol.

[133] Muñoz-Gómez Sergio A, Slamovits Claudio H, Dacks Joel B, Baier Kaitlyn A, Spencer Katelyn D, Wideman Jeremy G (2015). Ancient homology of the mitochondrial contact site and cristae organizing system points to an endosymbiotic origin of mitochondrial cristae. Curr Biol.

[134] Huynen MA, Mühlmeister M, Gotthardt K, Guerrero-Castillo S, Brandt U (1863). Evolution and structural organization of the mitochondrial contact site (MICOS) complex and the mitochondrial intermembrane space bridging (MIB) complex. Biochim Biophys Acta.

[135] Muñoz-Gómez SA, Wideman JG, Roger AJ, Slamovits CH (2017). The origin of mitochondrial cristae from Alphaproteobacteria. Mol Biol Evol.

[136] Li L, Lavell A, Meng X, Berkowitz O, Selinski J, van de Meene A, Carrie C, Benning C, Whelan J, De Clercq I (2019). Arabidopsis DGD1 SUPPRESSOR1 is a subunit of the mitochondrial contact site and cristae organizing system and affects mitochondrial biogenesis. Plant Cell.

[137] Leger MM, Petrů M, Žárský V, Eme L, Vlček Č, Harding T, Lang BF, Eliáš M, Doležal P, Roger AJ (2015). An ancestral bacterial division system is widespread in eukaryotic mitochondria. Proc Natl Acad Sci U S A.

[138] Elgass K, Pakay J, Ryan MT, Palmer CS (2013). Recent advances into the understanding of mitochondrial fission. Biochim Biophys Acta.

[139] Tondera D, Czauderna F, Paulick K, Schwarzer R, Kaufmann J, Santel A (2005). The mitochondrial protein MTP18 contributes to mitochondrial fission in mammalian cells. J Cell Sci.

[140] Morita M, Prudent J, Basu K, Goyon V, Katsumura S, Hulea L, Pearl D, Siddiqui N, Strack S, McGuirk S (2017). mTOR controls mitochondrial dynamics and cell survival via MTFP1. Mol Cell.

[141] Lee W-H, Higuchi H, Ikeda S, Macke EL, Takimoto T, Pattnaik BR, Liu C, Chu L-F, Siepka SM, Krentz KJ (2016). Mouse Tmem135 mutation reveals a mechanism involving mitochondrial dynamics that leads to age-dependent retinal pathologies. eLife..

[142] Žárský V, Doležal P (2016). Evolution of the Tim17 protein family. Biol Direct.

[143] Kimura M, Okano Y (2007). Human Misato regulates mitochondrial distribution and morphology. Exp Cell Res.

[144] Gal A, Balicza P, Weaver D, Naghdi S, Joseph SK, Várnai P, Gyuris T, Horváth A, Nagy L, Seifert EL (2017). MSTO1 is a cytoplasmic pro-mitochondrial fusion protein, whose mutation induces myopathy and ataxia in humans. EMBO Mol Med.

[145] Palumbo V, Pellacani C, Heesom KJ, Rogala KB, Deane CM, Mottier-Pavie V, Gatti M, Bonaccorsi S, Wakefield JG (2015). Misato controls mitotic microtubule generation by stabilizing the TCP-1 tubulin chaperone complex [corrected]. Curr Biol.

[146] Wiedemann N, Pfanner N (2017). Mitochondrial machineries for protein import and assembly. Annu Rev Biochem.

[147] Maćašev D, Whelan J, Newbigin E, Silva-Filho MC, Mulhern TD, Lithgow T (2004). Tom22′, an 8-kDa *trans*-site receptor in plants and protozoans, is a conserved feature of the TOM complex that appeared early in the evolution of eukaryotes. Mol Biol Evol.

[148] Dolezal P, Likic V, Tachezy J, Lithgow T (2006). Evolution of the molecular machines for protein import into mitochondria. Science..

[149] Mani J, Meisinger C, Schneider A (2016). Peeping at TOMs—diverse entry gates to mitochondria provide insights into the evolution of eukaryotes. Mol Biol Evol.

[150] Gebert M, Schrempp SG, Mehnert CS, Heißwolf AK, Oeljeklaus S, Ieva R, Bohnert M, von der Malsburg K, Wiese S, Kleinschroth T (2012). Mgr2 promotes coupling of the mitochondrial presequence translocase to partner complexes. J Cell Biol.

[151] Ieva R, Schrempp Sandra G, Opaliński Ł, Wollweber F, Höß P, Heißwolf Anna K, Gebert M, Zhang Y, Guiard B, Rospert S (2014). Mgr2 functions as lateral gatekeeper for preprotein sorting in the mitochondrial inner membrane. Mol Cell.

[152] Mirzalieva O, Jeon S, Damri K, Hartke R, Drwesh L, Demishtein-Zohary K, Azem A, Dunn CD, Peixoto PM (2019). Deletion of Mgr2p affects the gating behavior of the TIM23 complex. Front Physiol.

[153] Chung YM, Kim JS, Yoo YD (2006). A novel protein, Romo1, induces ROS production in the mitochondria. Biochem Biophys Res Commun.

[154] Jan P-S, Esser K, Pratje E, Michaelis G (2000). Som1, a third component of the yeast mitochondrial inner membrane peptidase complex that contains Imp1 and Imp2. Mol Gen Genet.

[155] Bauerschmitt H, Mick DU, Deckers M, Vollmer C, Funes S, Kehrein K, Ott M, Rehling P, Herrmann JM, Fox TD (2010). Ribosome-binding proteins Mdm38 and Mba1 display overlapping functions for regulation of mitochondrial translation. Mol Biol Cell.

[156] Greber BJ, Boehringer D, Leitner A, Bieri P, Voigts-Hoffmann F, Erzberger JP, Leibundgut M, Aebersold R, Ban N (2014). Architecture of the large subunit of the mammalian mitochondrial ribosome. Nature..

[157] Möller-Hergt BV, Carlström A, Stephan K, Imhof A, Ott M (2018). The ribosome receptors Mrx15 and Mba1 jointly organize cotranslational insertion and protein biogenesis in mitochondria. Mol Biol Cell.

[158] Duncan O, Murcha MW, Whelan J (2013). Unique components of the plant mitochondrial protein import apparatus. Biochim Biophys Acta.

[159] Schneider A (2018). Mitochondrial protein import in trypanosomatids: variations on a theme or fundamentally different?. PLoS Pathog.

[160] Longen S, Bien M, Bihlmaier K, Kloeppel C, Kauff F, Hammermeister M, Westermann B, Herrmann JM, Riemer J (2009). Systematic analysis of the twin Cx_9_C protein family. J Mol Biol.

[161] Fukasawa Y, Oda T, Tomii K, Imai K (2017). Origin and evolutionary alteration of the mitochondrial import system in eukaryotic lineages. Mol Biol Evol.

[162] Kang Y, Baker MJ, Liem M, Louber J, McKenzie M, Atukorala I, Ang C-S, Keerthikumar S, Mathivanan S, Stojanovski D (2016). Tim29 is a novel subunit of the human TIM22 translocase and is involved in complex assembly and stability. eLife..

[163] Gebert N, Gebert M, Oeljeklaus S, von der Malsburg K, Stroud David A, Kulawiak B, Wirth C, Zahedi René P, Dolezal P, Wiese S (2011). Dual function of Sdh3 in the respiratory chain and TIM22 protein translocase of the mitochondrial inner membrane. Mol Cell.

[164] Wenz L-S, Ellenrieder L, Qiu J, Bohnert M, Zufall N, van der Laan M, Pfanner N, Wiedemann N, Becker T (2015). Sam37 is crucial for formation of the mitochondrial TOM–SAM supercomplex, thereby promoting β-barrel biogenesis. J Cell Biol.

[165] Meisinger C, Pfannschmidt S, Rissler M, Milenkovic D, Becker T, Stojanovski D, Youngman MJ, Jensen RE, Chacinska A, Guiard B (2007). The morphology proteins Mdm12/Mmm1 function in the major β-barrel assembly pathway of mitochondria. EMBO J.

[166] Meisinger C, Wiedemann N, Rissler M, Strub A, Milenkovic D, Schönfisch B, Müller H, Kozjak V, Pfanner N (2006). Mitochondrial protein sorting: differentiation of β-barrel assembly by Tom7-mediated segregation of Mdm10. J Biol Chem.

[167] Mesecke N, Bihlmaier K, Grumbt B, Longen S, Terziyska N, Hell K, Herrmann JM (2008). The zinc-binding protein Hot13 promotes oxidation of the mitochondrial import receptor Mia40. EMBO Rep.

[168] Palmer T, Berks BC (2012). The twin-arginine translocation (tat) protein export pathway. Nat Rev Microbiol.

[169] Petrů M, Wideman J, Moore K, Alcock F, Palmer T, Doležal P (2018). Evolution of mitochondrial TAT translocases illustrates the loss of bacterial protein transport machines in mitochondria. BMC Biol.

[170] Carrie C, Weißenberger S, Soll J (2016). Plant mitochondria contain the protein translocase subunits TatB and TatC. J Cell Sci.

[171] Horváthová L, Žárský V, Pánek T, Derelle R, Pyrih J, Krupičková A, Klápšťová V, Klimeš V, Petrů M, Vaitová Z *et al*: Ancestral mitochondrial protein secretion machinery. bioRxiv. 2019;10.1101/790865.

[172] Lycklama A, Nijeholt AJ, Jelger A, Driessen AJ (2012). The bacterial Sec-translocase: structure and mechanism. Philos Trans R Soc Lond Ser B Biol Sci.

[173] Steinberg R, Knüpffer L, Origi A, Asti R, Koch H-G: Co-translational protein targeting in bacteria. FEMS Microbiol Lett. 2018;365:10.1093/femsle/fny095. 10.1093/femsle/fny095 29790984

[174] Palmieri F (2013). The mitochondrial transporter family SLC25: identification, properties and physiopathology. Mol Asp Med.

[175] Taylor EB (2017). Functional properties of the mitochondrial carrier system. Trends Cell Biol.

[176] KEGG Automatic Annotation Service (KAAS). [https://www.genome.jp/kaas-bin/kaas_main].

[177] Boulet A, Vest KE, Maynard MK, Gammon MG, Russell AC, Mathews AT, Cole SE, Zhu X, Phillips CB, Kwong JQ (2018). The mammalian phosphate carrier SLC25A3 is a mitochondrial copper transporter required for cytochrome *c* oxidase biogenesis. J Biol Chem.

[178] Monné M, Daddabbo L, Gagneul D, Obata T, Hielscher B, Palmieri L, Miniero DV, Fernie AR, Weber APM, Palmieri F (2018). Uncoupling proteins 1 and 2 (UCP1 and UCP2) from *Arabidopsis thaliana* are mitochondrial transporters of aspartate, glutamate, and dicarboxylates. J Biol Chem.

[179] Harborne SPD, Kunji ERS (2018). Calcium-regulated mitochondrial ATP-mg/P_i_ carriers evolved from a fusion of an EF-hand regulatory domain with a mitochondrial ADP/ATP carrier-like domain. IUBMB Life.

[180] Chaudhuri D, Sancak Y, Mootha VK, Clapham DE (2013). MCU encodes the pore conducting mitochondrial calcium currents. eLife..

[181] Kory Nora, Wyant Gregory A., Prakash Gyan, uit de Bos Jelmi, Bottanelli Francesca, Pacold Michael E., Chan Sze Ham, Lewis Caroline A., Wang Tim, Keys Heather R., Guo Yang Eric, Sabatini David M. (2018). SFXN1 is a mitochondrial serine transporter required for one-carbon metabolism. Science.

[182] Uzarska MA, Nasta V, Weiler BD, Spantgar F, Ciofi-Baffoni S, Saviello MR, Gonnelli L, Mühlenhoff U, Banci L, Lill R (2016). Mitochondrial Bol1 and Bol3 function as assembly factors for specific iron-sulfur proteins. eLife..

[183] Tamura Y, Harada Y, Nishikawa S-I, Yamano K, Kamiya M, Shiota T, Kuroda T, Kuge O, Sesaki H, Imai K (2013). Tam41 is a CDP-diacylglycerol synthase required for cardiolipin biosynthesis in mitochondria. Cell Metab.

[184] Blunsom NJ, Gomez-Espinosa E, Ashlin TG, Cockcroft S (1863). Mitochondrial CDP-diacylglycerol synthase activity is due to the peripheral protein, TAMM41 and not due to the integral membrane protein, CDP-diacylglycerol synthase 1. Biochim Biophys Acta.

[185] Tamura Y, Iijima M, Sesaki H (2010). Mdm35p imports ups proteins into the mitochondrial intermembrane space by functional complex formation. EMBO J.

[186] Yu F, He F, Yao H, Wang C, Wang J, Li J, Qi X, Xue H, Ding J, Zhang P (2015). Structural basis of intramitochondrial phosphatidic acid transport mediated by Ups1-Mdm35 complex. EMBO Rep.

[187] Taylor WA, Hatch GM (2009). Identification of the human mitochondrial linoleoyl-coenzyme a monolysocardiolipin acyltransferase (MLCL AT-1). J Biol Chem.

[188] Felts SJ, Owen BAL, Nguyen P, Trepel J, Donner DB, Toft DO (2000). The hsp90-related protein TRAP1 is a mitochondrial protein with distinct functional properties. J Biol Chem.

[189] Westermann B, Gaume B, Herrmann JM, Neupert W, Schwarz E (1996). Role of the mitochondrial DnaJ homolog Mdj1p as a chaperone for mitochondrially synthesized and imported proteins. Mol Cell Biol.

[190] Leonhard K., Herrmann J. M., Stuart R. A., Mannhaupt G., Neupert W., Langer T. (1996). AAA proteases with catalytic sites on opposite membrane surfaces comprise a proteolytic system for the ATP-dependent degradation of inner membrane proteins in mitochondria. The EMBO Journal.

[191] Cesnekova J, Rodinova M, Hansikova H, Houstek J, Zeman J, Stiburek L (2016). The mammalian homologue of yeast Afg1 ATPase (lactation elevated 1) mediates degradation of nuclear-encoded complex IV subunits. Biochem J.

[192] Rohrwild M, Coux O, Huang HC, Moerschell RP, Yoo SJ, Seol JH, Chung CH, Goldberg AL (1996). HslV-HslU: a novel ATP-dependent protease complex in *Escherichia coli* related to the eukaryotic proteasome. Proc Natl Acad Sci U S A.

[193] Lensch M, Herrmann RG, Sokolenko A (2001). Identification and characterization of SppA, a novel light-inducible chloroplast protease complex associated with thylakoid membranes. J Biol Chem.

[194] Cox Andrew G, Winterbourn Christine C, Hampton Mark B (2010). Mitochondrial peroxiredoxin involvement in antioxidant defence and redox signalling. Biochem J.

[195] Moseler A, Aller I, Wagner S, Nietzel T, Przybyla-Toscano J, Mühlenhoff U, Lill R, Berndt C, Rouhier N, Schwarzländer M (2015). The mitochondrial monothiol glutaredoxin S15 is essential for iron-sulfur protein maturation in *Arabidopsis thaliana*. Proc Natl Acad Sci U S A.

[196] Sztukowska M, Bugno M, Potempa J, Travis J, Kurtz DM (2002). Role of rubrerythrin in the oxidative stress response of *Porphyromonas gingivalis*. Mol Microbiol.

[197] Cooley RB, Arp DJ, Karplus PA (2011). Symerythrin structures at atomic resolution and the origins of rubrerythrins and the ferritin-like superfamily. J Mol Biol.

[198] Figueroa-Martinez F, Jackson C, Reyes-Prieto A (2018). Plastid genomes from diverse glaucophyte genera reveal a largely conserved gene content and limited architectural diversity. Genome Biol Evol..

[199] Maralikova B, Ali V, Nakada-Tsukui K, Nozaki T, Giezen MVD, Henze K, Tovar J (2010). Bacterial-type oxygen detoxification and iron-sulfur cluster assembly in amoebal relict mitochondria. Cell Microbiol.

[200] Stairs CW, Kokla A, Ástvaldsson Á, Jerlström-Hultqvist J, Svärd S, Ettema TJG (2019). Oxygen induces the expression of invasion and stress response genes in the anaerobic salmon parasite *Spironucleus salmonicida*. BMC Biol.

[201] Karnkowska A, Vacek V, Zubáčová Z, Treitli SC, Petrželková R, Eme L, Novák L, Žárský V, Barlow LD, Herman EK (2016). A eukaryote without a mitochondrial organelle. Curr Biol.

[202] Cardenas JP, Quatrini R, Holmes DS (2016). Aerobic lineage of the oxidative stress response protein rubrerythrin emerged in an ancient microaerobic, (hyper)thermophilic environment. Front Microbiol.

[203] Elguindy MM, Nakamaru-Ogiso E (2015). Apoptosis-inducing factor (AIF) and its family member protein, AMID, are rotenone-sensitive NADH:ubiquinone oxidoreductases (NDH-2). J Biol Chem.

[204] Seidi A, Muellner-Wong LS, Rajendran E, Tjhin ET, Dagley LF, Aw VYT, Faou P, Webb AI, Tonkin CJ, van Dooren GG (2018). Elucidating the mitochondrial proteome of *Toxoplasma gondii* reveals the presence of a divergent cytochrome *c* oxidase. eLife..

[205] Gray MW, Lang BF, Burger G (2004). Mitochondria of protists. Annu Rev Genet.

[206] Van der Giezen M (2009). Hydrogenosomes and mitosomes: conservation and evolution of functions. J Eukaryot Microbiol.

[207] Barberà MJ, Ruiz-Trillo I, Leigh J, Hug LA, Roger AJ, Martin WF, Müller M (2007). The diversity of mitochondrion-related organelles amongst eukaryotic microbes. Origin of mitochondria and Hydrogenosomes.

[208] Jedelský PL, Doležal P, Rada P, Pyrih J, Šmíd O, Hrdý I, Šedinová M, Marcinčiková M, Voleman L, Perry AJ (2011). The minimal proteome in the reduced mitochondrion of the parasitic protist *Giardia intestinalis*. PLoS One.

[209] Schneider RE, Brown MT, Shiflett AM, Dyall SD, Hayes RD, Xie Y, Loo JA, Johnson PJ (2011). The *Trichomonas vaginalis* hydrogenosome proteome is highly reduced relative to mitochondria, yet complex compared with mitosomes. Int J Parasitol.

[210] Stairs Courtney W., Leger Michelle M., Roger Andrew J. (2015). Diversity and origins of anaerobic metabolism in mitochondria and related organelles. Philosophical Transactions of the Royal Society B: Biological Sciences.

[211] Noguchi F, Shimamura S, Nakayama T, Yazaki E, Yabuki A, Hashimoto T, Inagaki Y, Fujikura K, Takishita K (2015). Metabolic capacity of mitochondrion-related organelles in the free-living anaerobic stramenopile *Cantina marsupialis*. Protist..

[212] Gawryluk Ryan MR, Kamikawa R, Stairs Courtney W, Silberman Jeffrey D, Brown Matthew W, Roger Andrew J (2016). The earliest stages of mitochondrial adaptation to low oxygen revealed in a novel rhizarian. Curr Biol.

[213] Gray MW, Lang BF, Cedergren R, Golding GB, Lemieux C, Sankoff D, Turmel M, Brossard N, Delage E, Littlejohn TG (1998). Genome structure and gene content in protist mitochondrial DNAs. Nucleic Acids Res.

[214] John P, Whatley FR (1975). *Paracoccus denitrificans* and the evolutionary origin of the mitochondrion. Nature..

[215] Ferguson SJ (2018). *Paracoccus denitrificans* oxidative phosphorylation: retentions, gains, losses, and lessons *en route* to mitochondria. IUBMB Life.

[216] Flegontov P, Gray MW, Burger G, Lukeš J (2011). Gene fragmentation: a key to mitochondrial genome evolution in Euglenozoa?. Curr Genet.

[217] Burger G, Valach M (2018). Perfection of eccentricity: mitochondrial genomes of diplonemids. IUBMB Life.

[218] Lang BF: Fungal Mitochondrial Genome Project (FMGP). http://megasun.bch.umontreal.ca/People/lang/FMGP/methods.html.

[219] Lang BF, Burger G (2007). Purification of mitochondrial and plastid DNA. Nat Protocols.

[220] Li H, Durbin R (2009). Fast and accurate short read alignment with Burrows–Wheeler transform. Bioinformatics..

[221] Simão FA, Waterhouse RM, Ioannidis P, Kriventseva EV, Zdobnov EM (2015). BUSCO: assessing genome assembly and annotation completeness with single-copy orthologs. Bioinformatics..

[222] Zdobnov EM, Tegenfeldt F, Kuznetsov D, Waterhouse RM, Simão FA, Ioannidis P (2017). OrthoDB v9.1: cataloging evolutionary and functional annotations for animal, fungal, plant, archaeal, bacterial and viral orthologs. Nucleic Acids Res.

[223] Eddy S. HMMER. [cited 2019 Dec 11]. Available from: http://hmmer.org/.

[224] Besemer J, Borodovsky M (1999). Heuristic approach to deriving models for gene finding. Nucleic Acids Res.

[225] Grabherr MG, Haas BJ, Yassour M, Levin JZ, Thompson DA, Amit I, Adiconis X, Fan L, Raychowdhury R, Zeng Q (2011). Full-length transcriptome assembly from RNA-Seq data without a reference genome. Nat Biotechnol.

[226] Bolger AM, Usadel B, Lohse M (2014). Trimmomatic: a flexible trimmer for Illumina sequence data. Bioinformatics..

[227] Song L, Florea L (2015). Rcorrector: efficient and accurate error correction for Illumina RNA-seq reads. GigaScience..

[228] Haas BJ, Delcher AL, Mount SM, Wortman JR, Smith RK, Hannick LI, Maiti R, Ronning CM, Rusch DB, Town CD (2003). Improving the *Arabidopsis* genome annotation using maximal transcript alignment assemblies. Nucleic Acids Res.

[229] GitHub. trinityrnaseq. https://github.com/trinityrnaseq.

[230] GitHub. PASA pipeline. https://github.com/PASApipeline.

[231] Haas BJ, Zeng Q, Pearson MD, Cuomo CA, Wortman JR (2011). Approaches to fungal genome annotation. Mycology..

[232] Gotoh O (2008). A space-efficient and accurate method for mapping and aligning cDNA sequences onto genomic sequence. Nucleic Acids Res.

[233] Stanke M, Waack S (2003). Gene prediction with a hidden Markov model and a new intron submodel. Bioinformatics.

[234] Korf I (2004). Gene finding in novel genomes. BMC Bioinformatics.

[235] Lomsadze A, Burns PD, Borodovsky M (2014). Integration of mapped RNA-Seq reads into automatic training of eukaryotic gene finding algorithm. Nucleic Acids Res.

[236] Testa AC, Hane JK, Ellwood SR, Oliver RP (2015). CodingQuarry: highly accurate hidden Markov model gene prediction in fungal genomes using RNA-seq transcripts. BMC Genomics.

[237] Luciani A, Lopez R, Potter SC, Park Y, Finn RD, Eddy SR (2018). HMMER web server: 2018 update. Nucleic Acids Res.

[238] El-Gebali S, Mistry J, Bateman A, Eddy SR, Luciani A, Potter SC, Qureshi M, Richardson LJ, Salazar GA, Smart A (2018). The Pfam protein families database in 2019. Nucleic Acids Res.

[239] Zimmermann L, Stephens A, Nam S-Z, Rau D, Kübler J, Lozajic M, Gabler F, Söding J, Lupas AN, Alva V (2018). A completely reimplemented MPI bioinformatics toolkit with a new HHpred server at its core. J Mol Biol.

[240] Kelley LA, Mezulis S, Yates CM, Wass MN, Sternberg MJE (2015). The Phyre2 web portal for protein modeling, prediction and analysis. Nat Protoc.

[241] Emanuelsson O, Brunak S, von Heijne G, Nielsen H (2007). Locating proteins in the cell using TargetP, SignalP and related tools. Nature Protoc.

[242] Claros M, Vincens P (1996). Computational method to predict mitochondrially imported proteins and their targeting sequences. Eur J Biochem.

[243] Small I, Peeters N, Legeai F, Lurin C (2004). Predotar: a tool for rapidly screening proteomes for N-terminal targeting sequences. Proteomics..

[244] Nakai K, Horton P (1999). PSORT : a program for detecting sorting signals in proteins and predicting their subcellular localization. Trends Biochem Sci.

[245] Petsalaki EI, Bagos PG, Litou ZI, Hamodrakas SJ (2006). PredSL: a tool for the N-terminal sequence-based prediction of protein subcellular localization. Genomics Proteomics Bioinformatics.

[246] Fukasawa Y, Tsuji J, Fu S-C, Tomii K, Horton P, Imai K (2015). MitoFates: improved prediction of mitochondrial targeting sequences and their cleavage sites. Mol Cell Proteomics.

[247] Katoh K, Standley DM (2013). MAFFT multiple sequence alignment software version 7: improvements in performance and usability. Mol Biol Evol.

[248] Capella-Gutiérrez S, Silla-Martínez JM, Gabaldón T (2009). trimAl: a tool for automated alignment trimming in large-scale phylogenetic analyses. Bioinformatics..

[249] Stamatakis A (2014). RAxML version 8: a tool for phylogenetic analysis and post-analysis of large phylogenies. Bioinformatics..

[250] Price MN, Dehal PS, Arkin AP (2010). FastTree 2 – approximately maximum-likelihood trees for large alignments. PLoS One.

[251] Gray MW, Burger G, Derelle R, Klimes V, Leger MM, Sarrasin M. et al. Supplementary Datasets. 2020. *Andalucia godoyi* genome, transcriptome and proteome data. [http://megasun.bch.umontreal.ca/Andalucia_godoyi].

[252] Gray MW, Burger G, Derelle R, Klimes V, Leger MM, Sarrasin M. et al. Supplementary Datasets. 2020. NCBI BioProject accession: PRJNA559352 [https://www.ncbi.nlm.nih.gov/bioproject/PRJNA559352].

[253] Derelle R. Supplementary Datasets. 2020. *Andalucia godoyi* mitochondrial protein alignments and maximum lIkelihood trees. figshare. [https://figshare.com/authors/romain_derelle/6898412].

