# Supplementary material for: The draft nuclear genome sequence and predicted mitochondrial proteome of Andalucia godoyi, a protist with the most gene-rich and bacteria-like mitochondrial genome
Source: BMC Biol. 2020 Mar 2;18:22. doi: 10.1186/s12915-020-0741-6 (PMC7050145; doi:10.1186/s12915-020-0741-6)

(A) Pyruvate kinase (348 amino acid positions in alignment)

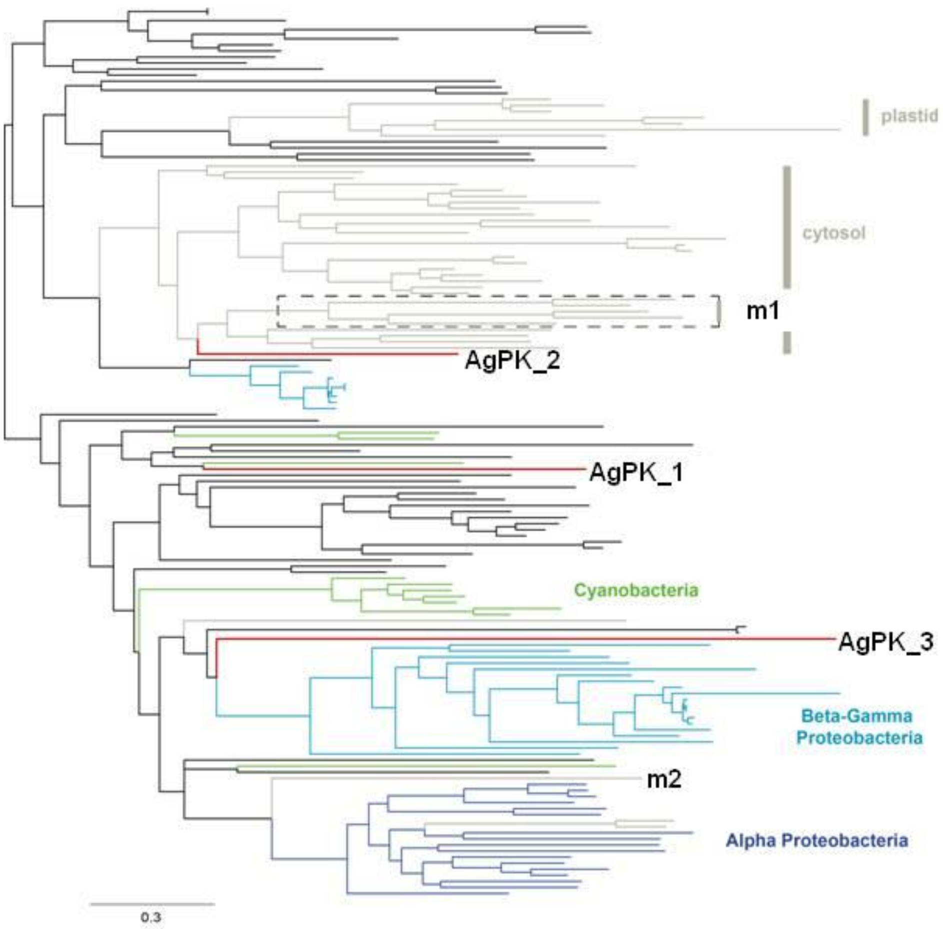

A. godoyi encodes three pyruvate kinases: AgPK_1 (ANDO_05259, mitochondrial) ; AgPK_2 (ANDGO_08019, cytosolic type); and AgPK_3 (ANDGO_06998, bacterial type). Positions of mitochondrion-targeted pyruvate kinases in other studies: m1, Abrahamian et al. [39]; m2, Saito et al. [37].

(B) **RecA** (155 amino acid positions in alignment)

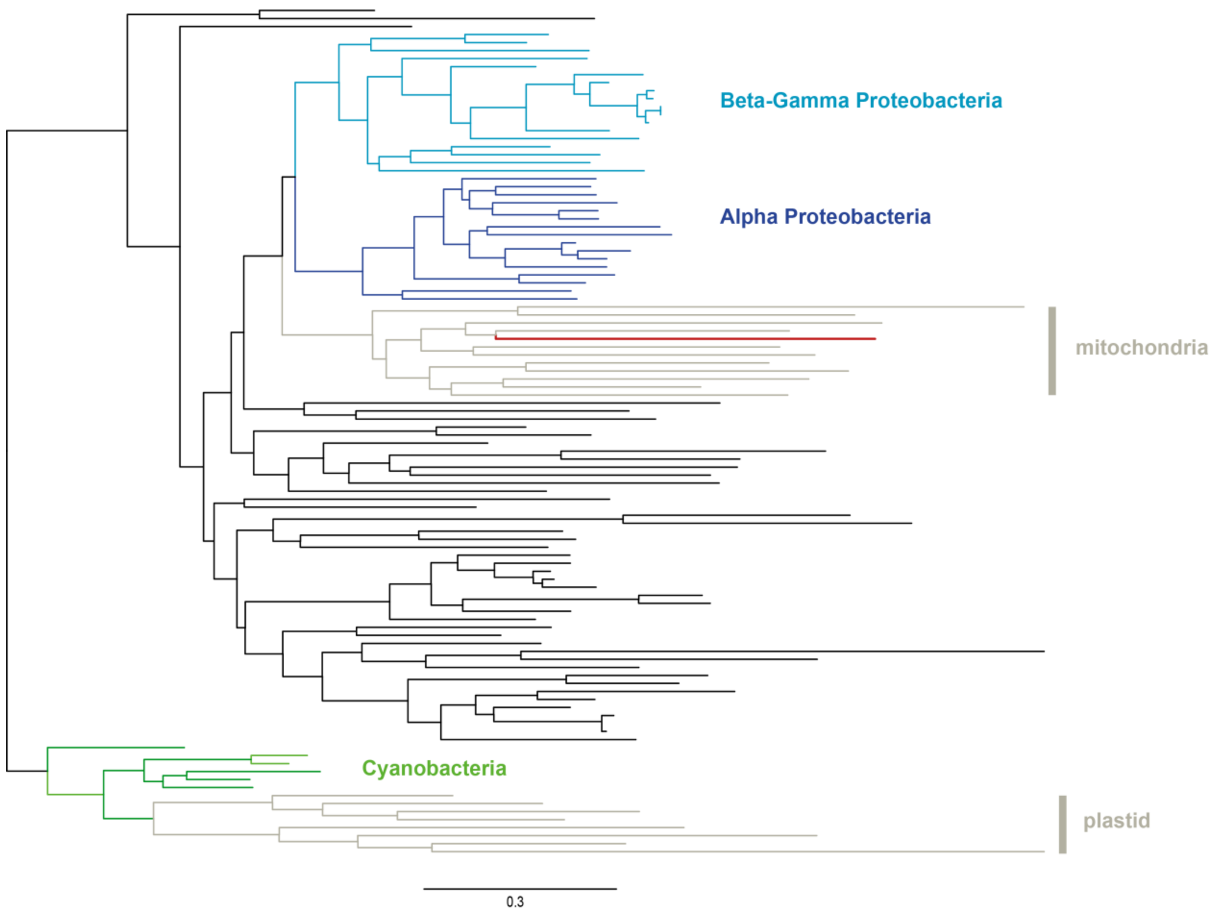

(C) **SmpB** (475 amino acid positions in alignment)

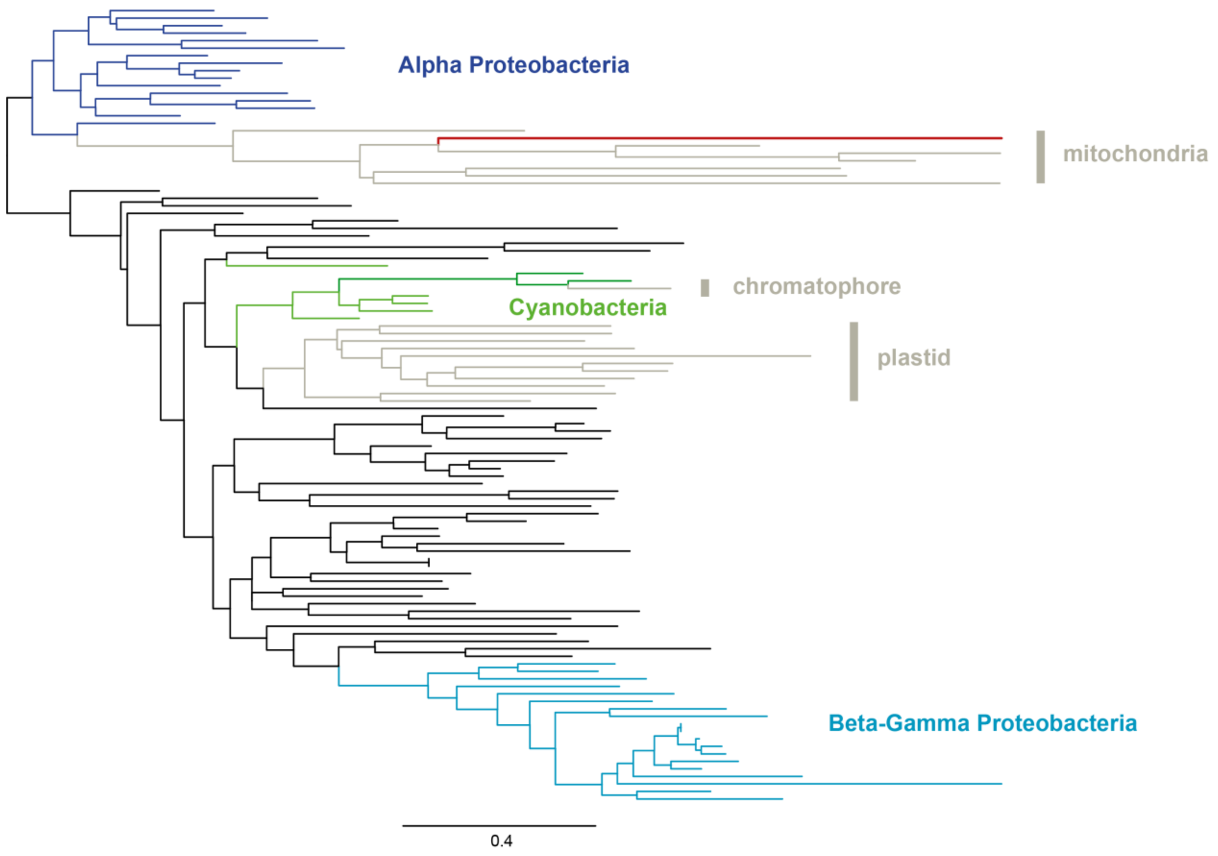

Supplement: Supplementary file 3 — Additional file 3: Figure S1. Phylogenetic analysis of selected A. godoyi mitochondrial protein sequences: (A) pyruvate kinase; (B) RecA; (C) SmpB. See Methods section for details of the analysis. The best ML trees are shown without support values. A. godoyi sequences and other eukaryotic sequences are coloured red and grey, respectively. Cyanobacteria and Alpha and Beta-Gamma Proteobacteria are coloured green and blue, respectively. Other bacterial sequences are coloured black. (PDF 307 kb) [file 12915_2020_741_MOESM3_ESM.pdf]
