# Supplementary material for: The draft nuclear genome sequence and predicted mitochondrial proteome of Andalucia godoyi, a protist with the most gene-rich and bacteria-like mitochondrial genome
Source: BMC Biol. 2020 Mar 2;18:22. doi: 10.1186/s12915-020-0741-6 (PMC7050145; doi:10.1186/s12915-020-0741-6)
Supplement: Supplementary file 5 — Additional file 5: Figure S2. A novel patchily distributed mitochondrial protein of the DegT/DnrJ/EryC1/StrS aminotransferase family. (A) Phylogenetic analysis of a selection of protein sequences of the DegT/DnrJ/EryC1/StrS aminotransferase family including the Andalucia godoyi protein ANDGO_00275.mRNA.1 and its relatives from other eukaryotes (identified by a near-exhaustive searching of available eukaryotic genomic and transcriptomic data). The tree was inferred using FastTree (default parameters) from an alignment of 372 amino acid positions. Black dots denote bootstrap support ≥91%. Branches in black correspond to sequences from prokaryotes, those in red are eukaryotic (a detailed view of the eukaryotic clade is provided at the top). (B) ANDGO_00275.mRNA.1 and its three eukaryotic relatives exhibit an N-terminal extension compared to their prokaryotic homologs (top) and the four proteins are predicted as mitochondrial by most tools employed (bottom; scores of four prediction tools are provided, with only PSORT II prediction for the T. trahens protein favouring cytosolic localization over the mitochondrial one). (PDF 439 kb) [file 12915_2020_741_MOESM5_ESM.pdf]

A

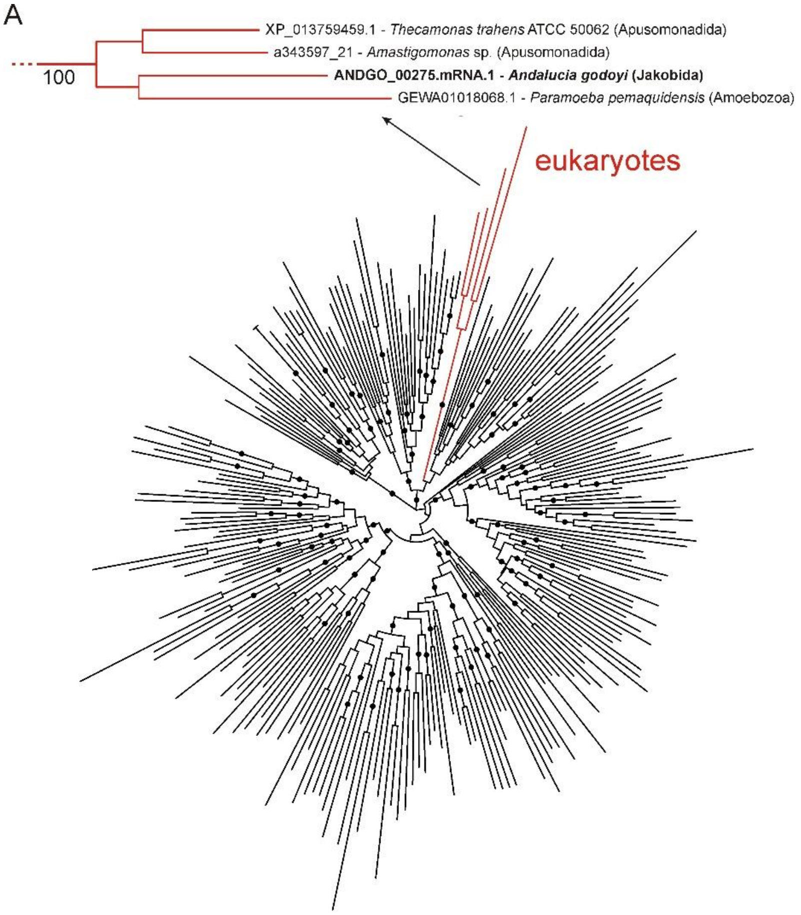

B

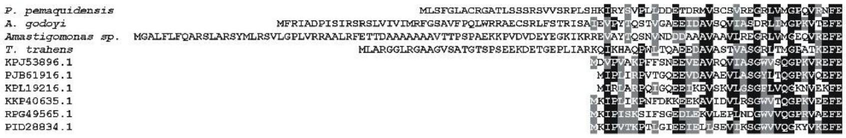

	MitoProt	TargetP	Predotar	PSORT II
Paramoeba pemaquidensis	0.9673	0.956	0.77	60.90%
Andalucia godoyi	0.7043	0.885	0.57	65.20%
Amastigomonas sp.	0.9993	0.974	0.52	69.60%
Thecamonas trahens	0.7165	0.526	0.57	34.80%
