# Supplementary figures and images for: The draft nuclear genome sequence and predicted mitochondrial proteome of Andalucia godoyi, a protist with the most gene-rich and bacteria-like mitochondrial genome
Source: BMC Biol. 2020 Mar 2;18:22. doi: 10.1186/s12915-020-0741-6 (PMC7050145; doi:10.1186/s12915-020-0741-6)

# (A) RFO (365 amino acid positions in alignment)

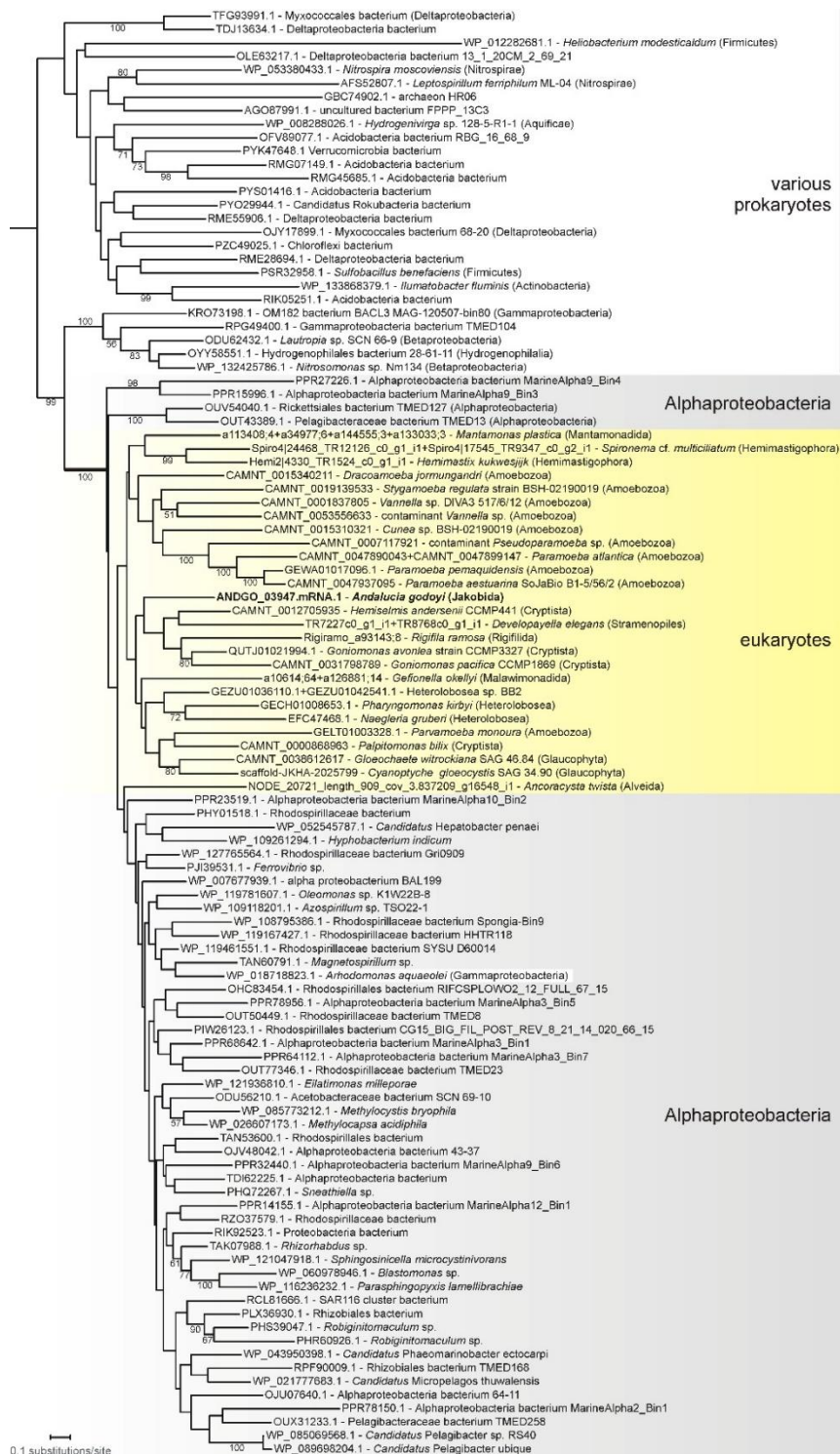

**(B) DUF3501 (128 amino acid positions in alignment)**

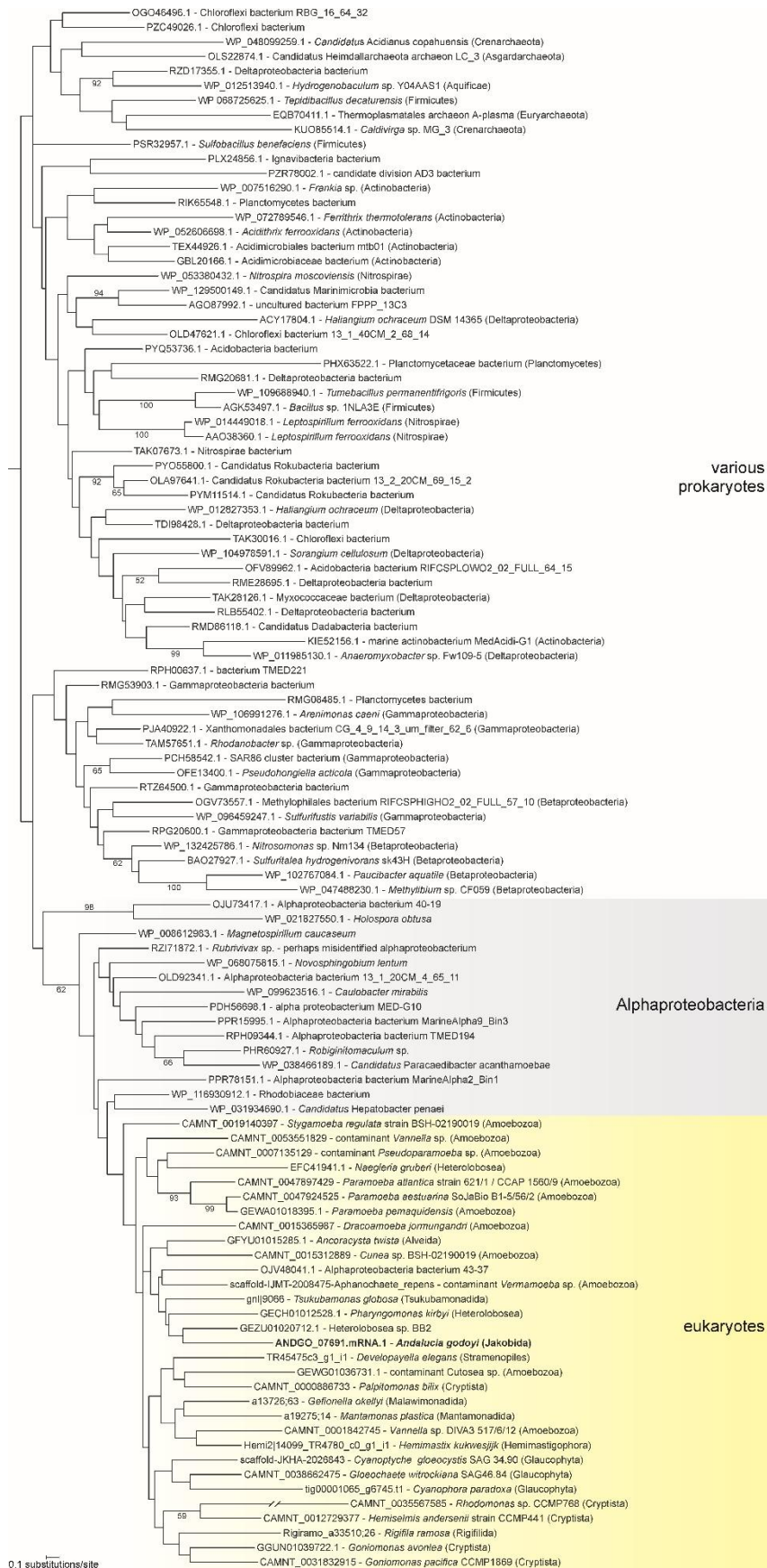

Supplement: Supplementary file 7 — Additional file 7: Figure S3. Phylogenetic analysis of (A) RFO and (B) DUF3501 proteins. ML trees (RAxML, PROTGAMMALG) are shown, with branch support values (rapid bootstraps) indicated when ≥51%. The root is placed arbitrarily. The RFO tree (A) is based on an alignment of 365 amino acid positions. The sequence from Ancoracysta twista is only partial, possibly explaining why it does not cluster together with sequences from other eukaryotes. Note the sequence from the gammaproteobacterium Arhodomonas aquaeolei nested among alphaproteobacterial sequences, suggesting a possible HGT event. The DUF3501 tree (B) is based on an alignment of 128 amino acid positions. (PDF 864 kb) [file 12915_2020_741_MOESM7_ESM.pdf]
